# Nickel-Catalyzed Cyclization/Carbonylation Reaction of *N*-Allylbromoacetamides with Arylboronic Acids toward 2-Pyrrolidinones

**DOI:** 10.1021/acs.orglett.5c00003

**Published:** 2025-01-24

**Authors:** Hucheng Ma, Chen-Yang Hou, Ruyi Zhao, Xinxin Qi, Xiao-Feng Wu

**Affiliations:** † School of Chemistry and Chemical Engineering, Key Laboratory of Surface & Interface Science of Polymer Materials of Zhejiang Province, Zhejiang Sci-Tech University, Hangzhou, Zhejiang 310018, People’s Republic of China; ‡ Dalian National Laboratory for Clean Energy, Dalian Institute of Chemical Physics, Chinese Academy of Sciences, 116023 Dalian, Liaoning, People’s Republic of China; § Leibniz-Institut für Katalyse e.V., Albert-Einstein-Straße 29a, Rostock 18059, Germany

## Abstract

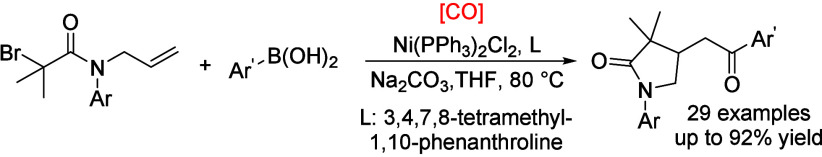

A straightforward and efficient nickel-catalyzed cyclization/carbonylation transformation of *N*-allylbromoacetamides toward the synthesis of 2-pyrrolidinone derivatives has been developed with arylboronic acids as the reaction partner. This transformation proceeds through a sequential single-electron-transfer pathway via 5-*exo*-*trig* cyclization and carbonyl insertion steps, furnishing a variety of 2-pyrrolidinone derivatives in good yields. Various useful functional groups were well tolerated. Moreover, formic acid is applied as the CO source here with nickel as the catalyst, which provides a good supplement for carbonylation chemistry and heterocycle synthesis.

2-Pyrrolidinones are prominent heterocycles which are found in numerous naturally occurring products, bioactive compounds, pharmaceutical chemicals,^1^ and have attracted a lot of attention for their outstanding biological activities.^2^ As shown in Figure 1, (−)-pramanicin is a natural product found from a fungus that exerts good antimicrobial activity.^3^ (−)-Clausenamide was active in the treatment of Alzheimer’s disease due to its good neuroprotective activity against Aβ_25–35_ which was originally isolated from *Clausena lansium* Skeels’ leaves.^4^ Brivaracetam is one of the main anticonvulsant drugs that has been used as an adjuvant therapy for the treatment of partial onset seizures.^5^ In addition, they have also been extensively used as synthetic intermediates in organic chemistry and drug design.^6,7^ In this regard, numerous efforts have been put forth for the synthesis of 2-pyrrolidinones,^8^ and among these, radical cyclization/coupling reactions have gradually emerged as one of the most useful and effective methods in recent years.^9^ Although these transformations have been intensively used in this field, a compelling need remains for the exploration of more efficient and straightforward strategies to access 2-pyrrolidinones with various functional groups.

**Figure 1:**
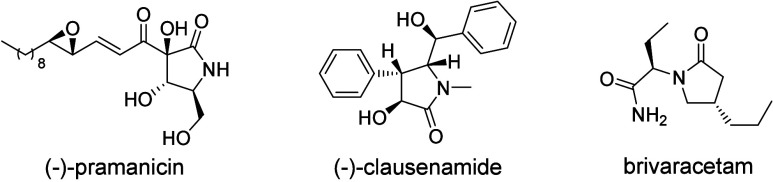
Selected examples of naturally occurring and bioactive 2-pyrrolidinones.

Transition metal-catalyzed carbonylative transformations have been well recognized due to their powerfulness in the synthesis of carbonylated chemicals and have gained much attention from both academic and industrial areas.^10^ In general, expensive metal catalysts, such as palladium, rhodium, and ruthenium, are commonly applied catalysts in this chemistry due to their outstanding reactivity. However, the high costs of noble metals usually restrict their applications in large scale usage. Therefore, the application of low-cost metals is highly desired. Nickel, as an abundant and low-price metal, was proven to be effective toward certain bonds and could be used in a variety of transformations.^11^ Nevertheless, the study of nickel catalysts in carbonylation reactions is still rare. Among these reasons, the formation of toxic and volatile Ni(CO)_4_ takes the responsibility, which is fully coordinated and will deactivate the reactivity of nickel catalysts.^12^ To circumvent this issue, decreasing the carbon monoxide pressure or using a CO source provides one good option. In 2019, Zhang’s group reported the first nickel-catalyzed radical carbonylation reaction of ethyl difluorobromoacetate with arylboronic acid under 1 atm CO atmosphere.^13^ After that, the same group and other research groups, such as Liang,^14^ Chen,^15^ Guo,^16^ etc.,^17^ developed a series of radical carbonylation reactions with nickel catalysis under low-pressure CO gas, or CO surrogates. Inspired by the aforementioned works, along with the distinct bioactivities of 2-pyrrolidinone skeletons and our continuous interest in nickel-catalyzed carbonylation reactions,^18^ we developed a nickel-catalyzed radical cyclization and carbonylation reaction of *N*-allylbromoacetamides with arylboronic acids for the synthesis of 2-pyrrolidinone derivatives.

Initially, for establishing reaction conditions, the tests were carried out with *N*-allyl-2-bromo-2-methyl-*N*-phenylpropanamide **1a** and *p*-tolylboronic acid **2a**. Unfortunately, the expected product **3a** was not detected with Ni(PPh_3_)_2_Cl_2_ as the precatalyst in the presence of dtbbpy ligand, and using K_2_CO_3_ as the base at 80 °C for 20 h in 1,4-dioxane (Table 1, entry 1). Then, various solvents were screened (Table 1, entries 2–5), and a trace amount of the targeted product was observed in THF (Table 1, entry 2). To our delight, except for Na_3_PO_4_, the yields of the target product could increase to 25–39% by using KHCO_3_, Na_2_CO_3_, Et_3_N, and DBU as the base (Table 1, entries 6–10). Subsequently, the effect of ligands was verified (Table 1, entries 11–15), resulting in product **3ab** in much higher yields when using L1 as the ligand (Table 1, entry 12). Different nickel precatalysts were tested, including NiCl_2_, NiBr_2_, Ni(OTf)_2_, and Ni(acac)_2_, but no further improvement was achieved (Table 1, entries 16–19). It was noteworthy that the reaction time has a substantial effect on the reaction outcome; the final product can be isolated in 84% yield after 16 h (Table 1, entry 20). Upon changing the amount of Na_2_CO_3_ to 1.5 equiv, an 86% yield of **3ab** was generated (Table 1, entry 21).

**Table 1:**
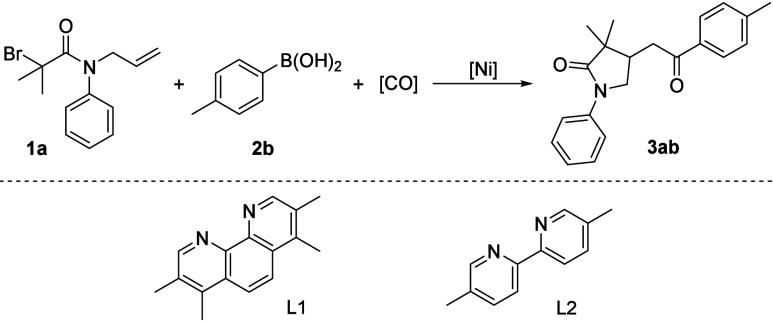
Screening of Reaction Conditions^a^

entry	catalyst	ligand	base	solvent	yield (%)
1	Ni(PPh_3_)_2_Cl_2_	dtbbpy	K_2_CO_3_	1,4-dioxane	0
2	Ni(PPh_3_)_2_Cl_2_	dtbbpy	K_2_CO_3_	THF	trace
3	Ni(PPh_3_)_2_Cl_2_	dtbbpy	K_2_CO_3_	CH_3_CN	0
4	Ni(PPh_3_)_2_Cl_2_	dtbbpy	K_2_CO_3_	DMF	0
5	Ni(PPh_3_)_2_Cl_2_	dtbbpy	K_2_CO_3_	toluene	0
6	Ni(PPh_3_)_2_Cl_2_	dtbbpy	KHCO_3_	THF	30
7	Ni(PPh_3_)_2_Cl_2_	dtbbpy	Na_2_CO_3_	THF	39
8	Ni(PPh_3_)_2_Cl_2_	dtbbpy	Na_3_PO_4_	THF	trace
9	Ni(PPh_3_)_2_Cl_2_	dtbbpy	Et_3_N	THF	25
10	Ni(PPh_3_)_2_Cl_2_	dtbbpy	DBU	THF	30
11	Ni(PPh_3_)_2_Cl_2_	bpy	Na_2_CO_3_	THF	20
12	Ni(PPh_3_)_2_Cl_2_	L1	Na_2_CO_3_	THF	75
13	Ni(PPh_3_)_2_Cl_2_	L2	Na_2_CO_3_	THF	30
14	Ni(PPh_3_)_2_Cl_2_	1,10-phen	Na_2_CO_3_	THF	71
15	Ni(PPh_3_)_2_Cl_2_	tpy	Na_2_CO_3_	THF	45
16	NiCl_2_	L1	Na_2_CO_3_	THF	73
17	NiBr_2_	L1	Na_2_CO_3_	THF	60
18	Ni(OTf)_2_	L1	Na_2_CO_3_	THF	60
19	Ni(acac)_2_	L1	Na_2_CO_3_	THF	59
20^b^	Ni(PPh_3_)_2_Cl_2_	L1	Na_2_CO_3_	THF	84
21^c^	Ni(PPh_3_)_2_Cl_2_	L1	Na_2_CO_3_	THF	86

a Reaction conditions: **1a** (0.2 mmol), **2b** (0.3 mmol), catalyst (10 mol %), ligand (10 mol %), [CO] (HCOOH + Ac_2_O, 2 mmol), base (2.0 equiv), solvent (2 mL), 80 °C, 20 h, isolated yields.

b 16 h.

c Na_2_CO_3_ (1.5 equiv).

After establishing the best reaction conditions, various arylboronic acids were applied to explore the generality of this transformation (Scheme 1). Phenylboronic acid gave the desired product in an excellent yield (**3aa**). Arylboronic acids substituted with electron-donating groups, including methyl, methoxy *tert*-butyl, and trifluoromethoxy could gave the target products in 35–85% yields (**3ab**–**3ag**). Substrates with *para-* and *meta-*substituents gave higher yields of the final products compared with *ortho*-substituted cases, which was probably due to the steric effect (**3ab**, **3ad** vs. **3ac**). Aryl boronic acids with electron-deficient groups, such as cyano, formyl, and trifluoro, were also tolerated to furnish the expected products in 40–76% yields (**3ah**–**3aj**). For halo groups, an 87% yield of product **3ak** was obtained with fluoro as the substituent, while low yields were observed with chloro and bromo as the substituents (**3al**, **3am**). Biphenyl and 2-naphthalenyl substrates were also checked, and the corresponding products were generated in 64% and 82% (**3an**, **3ao**). In addition, this reaction was carried out with thiophen-3-ylboronic acid as the starting material, and the targeted product was isolated in 46% yield (**3ap**). However, very low yield of the desired product was obtained when 4-pyridinylboronic acid, 3-pyridinylboronic acid or alkylboronic acid was tested as the substrate, and pyridine was detected as the byproduct in the cases with pyridine related substrates.

**Scheme 1:**
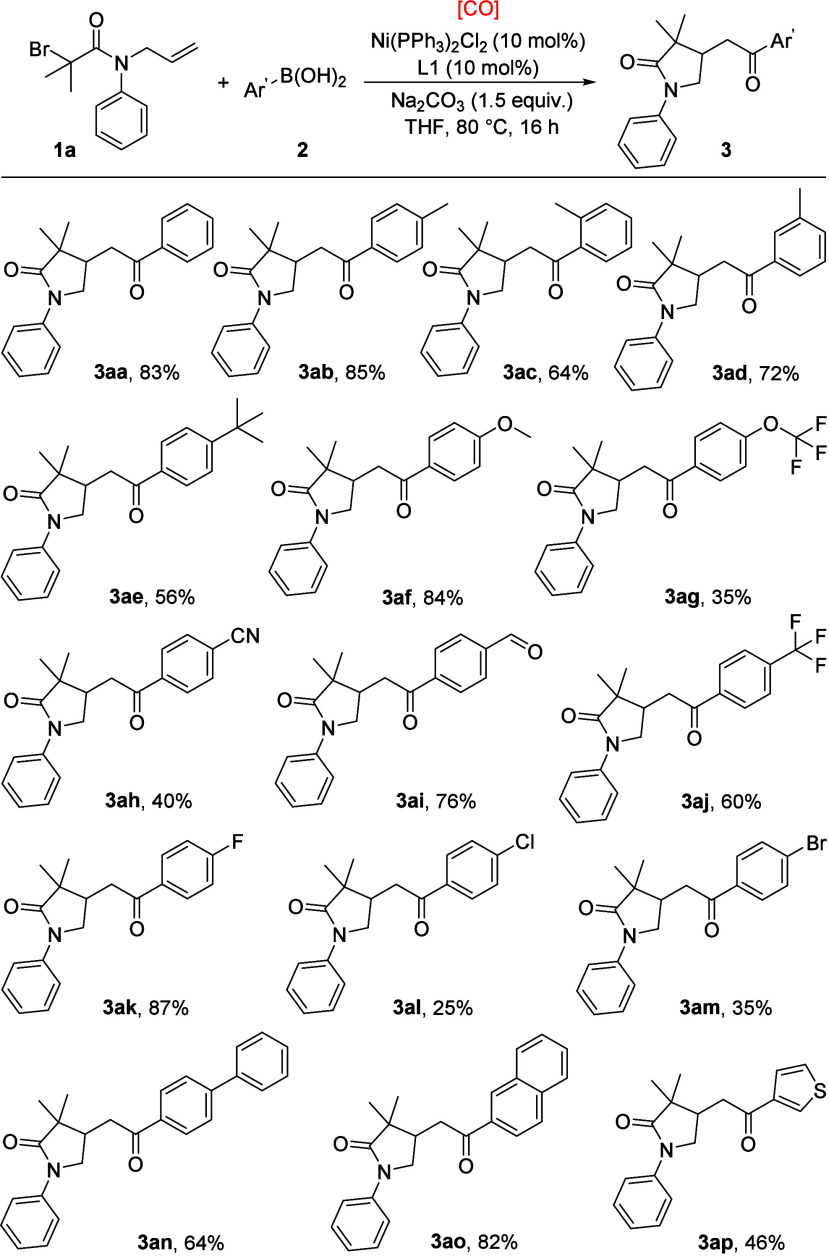
Substrate Scope of Arylboronic Acids Reaction conditions: *N*-allyl-2-bromo-2-methyl-*N*-phenylpropanamide **1a** (0.2 mmol), arylboronic acids **2** (0.3 mmol), Ni(PPh_3_)_2_Cl_2_ (10 mol %), L1 (10 mol %), [CO] (HCOOH + Ac_2_O, 2 mmol), Na_2_CO_3_ (1.5 equiv), THF (2 mL), 80 °C, 16 h. Isolated yield.

Various *N*-allylbromoacetamides were tested under our standard conditions subsequently, and a series of aryl substituents on *N*-allyl were screened and are summarized in Scheme 2. Aryl moieties with both electron-rich and electron-withdrawing groups were transformed into the targeted heterocycles in 55–92% yields (**3bb**–**3bg**). Halo groups, including fluoro, chloro, and bromo, could react with *p*-tolylboronic acid **2b** successfully to deliver the expected products in high yields (**3bh**–**3bj**). Biphenyl substrate was then examined, and product **3bk** was obtained in moderate yields. Notably, disubstituted groups, such as 3,5-dimethyl and 2,4-dimethyl could be efficiently converted to **3bl** and **3bm** in 78% and 85% yields. Additionally, a substrate bearing a benzo[*d*][1,3]dioxol-5-yl substituent was capable of affording the corresponding product in very good yield (**3bn**). The aryl group on nitrogen and the two-methyl group seems to be important, and no reaction occurred when it has been replaced with alkyl group or removed.

**Scheme 2:**
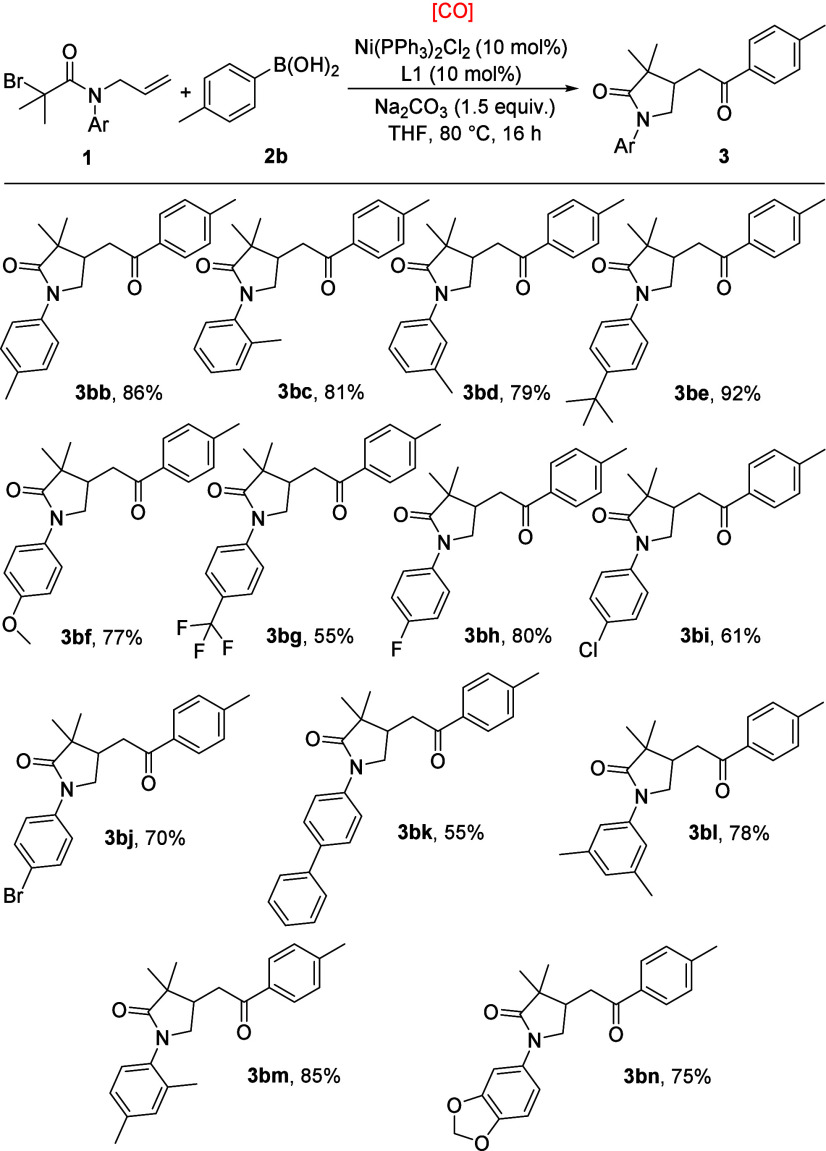
Substrate Scope of *N*-Allylbromoacetamides Reaction conditions: *N*-allylbromoacetamides **1** (0.2 mmol), *p*-tolylboronic acid **2b** (0.3 mmol), Ni(PPh_3_)_2_Cl_2_ (10 mol %), L1 (10 mol %), [CO] (HCOOH + Ac_2_O, 2 mmol), Na_2_CO_3_ (1.5 equiv), THF (2 mL), 80 °C, 16 h. Isolated yield.

Afterward, a control experiment was performed to get a better understanding of the reaction pathway (Scheme 3). No desired heterocyclic product could be observed when TEMPO as a radical scavenger was added. This result shows that radical intermediates were mostly involved during the transformation.

**Scheme 3:**
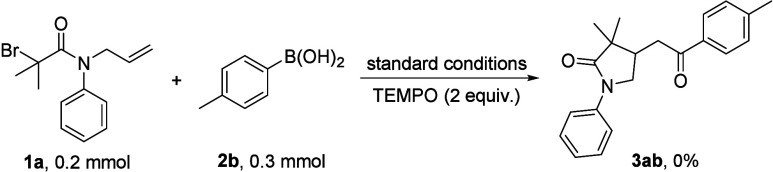
Control Experiment

Finally, a possible reaction mechanism is proposed based on our results (Scheme 4). First, transmetalation of arylboronic acids **2** with Ni(I) provides aryl Ni(I) complexes **I**, followed by a SET process with *N*-allylbromoacetamides **1** to give radicals **A** and aryl Ni(II) species **II**. Then a 5-*exo*-*trig* cyclization of **A** occurs to afford radicals **B**. Meanwhile, a coordination and insertion of CO to intermediates **II** gives acyl Ni(II) complexes **III**, which then undergo free radical addition with radicals **B** to furnish intermediates **IV**. Finally, reductive elimination of intermediates **IV** delivers target product **3** and regenerates Ni(I) to complete the catalytic cycle.

**Scheme 4:**
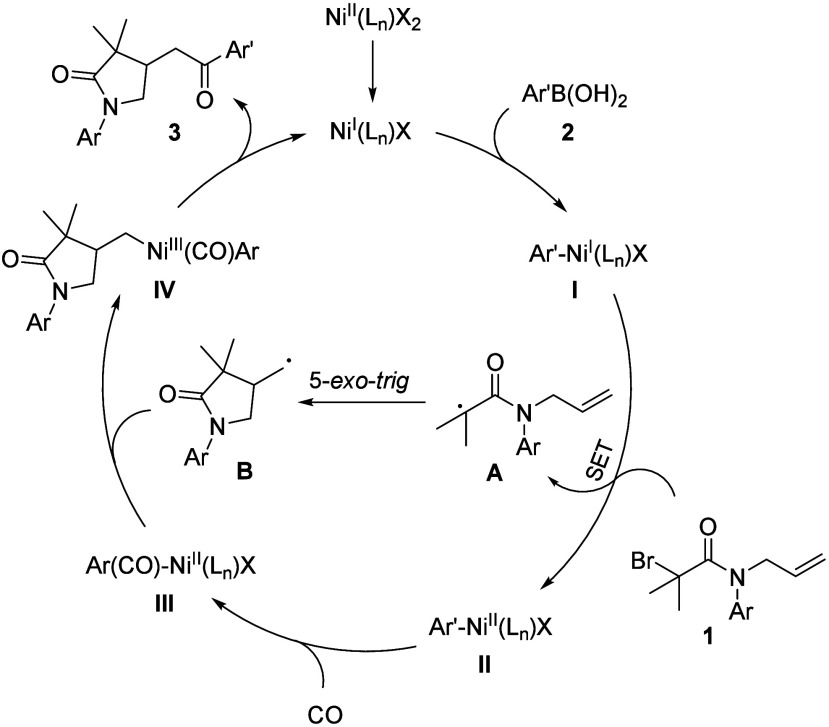
Plausible Reaction Mechanism

In summary, an efficient synthesis of 2*-*pyrrolidinone derivatives has been explored via a nickel-catalyzed radical cyclization/carbonylation reaction of *N*-allylbromoacetamides with arylboronic acids. By using formic acid as the CO surrogate, a diverse set of 2-pyrrolidinone derivatives were produced in good yields in general. This reaction features mild reaction conditions, high functional group compatibility, and no manipulation of carbon monoxide gas, which make it a good addition to access 2*-*pyrrolidinone derivatives through a nickel-catalyzed carbonylation reaction.

## Data Availability

The data underlying this study are available in the published article and its Supporting Information.

## Supporting Information Available

General comments, general procedure, analytic data and NMR spectra (PDF)
