# Supplementary material for: Nickel-Catalyzed Cyclization/Carbonylation Reaction of N-Allylbromoacetamides with Arylboronic Acids toward 2-Pyrrolidinones
Source: Org Lett. 2025 Jan 24;27(5):1299–303. doi: 10.1021/acs.orglett.5c00003 (PMC11812007; doi:10.1021/acs.orglett.5c00003)

Supporting Information

Nickel-Catalyzed Cyclization/Carbonylation Reaction of *N*-Allylbromoacetamides with Arylboronic Acids toward 2-Pyrrolidinones

Hucheng Ma,† Chen-Yang Hou,† Ruyi Zhao,† Xinxin Qi,*,† Xiao-Feng Wu*,‡

†School of Chemistry and Chemical Engineering, Key Laboratory of Surface & Interface Science of Polymer Materials of Zhejiang Province, Zhejiang Sci-Tech University, Hangzhou, Zhejiang 310018, People's Republic of China; E-mail: xinxinqi@zstu.edu.cn

‡Dalian National Laboratory for Clean Energy, Dalian Institute of Chemical Physics, Chinese Academy of Sciences, 116023, Dalian, Liaoning, China; Leibniz-Institut für Katalyse e.V., Albert-Einstein-Straße 29a, Rostock 18059, Germany; E-mail: xiao-feng.wu@catalysis.de

Table of Contents

1. General Information S2

2. General Procedures S3

2.1 General Procedure for the Synthesis of *N*-allylbromoacetamides S3

2.2 General Procedure for the Synthesis of Products S4

3. Characterization Data of Products S5

4. Reference S23

5. Copy of ^{1}H and ^{13}C NMR Spectra of Products S24

1. General Information

Unless otherwise noted, all reactions were carried out under N_2 atmosphere. All reagents were from commercial sources (Bidepharm, Energy Chemical, jkchemical) and used as received without further purification. All solvents were dry solvents. Column chromatography was performed on silica gel (200-300 meshes) using petroleum ether and ethyl acetate as eluent. NMR spectra were recorded on a Bruker Avance operating at for ^{1}H NMR at 400 MHz, ^{13}C NMR at 101 MHz and spectral data were reported in ppm relative to tetramethylsilane (TMS) as internal standard and $CDCl_3$ (^{1}H NMR δ 7.26, ^{13}C NMR δ 77.16) as solvent. All coupling constants (J) are reported in Hz. The following abbreviations were used to describe peak splitting patterns when appropriate: s = singlet, d = doublet, dd = double doublet, ddd = double doublet of doublets, t = triplet, dt = double triplet, q = quartet, m = multiplet, br = broad. Gas chromatography (GC) analyses were performed on a Shimadzu GC-2014C chromatograph equipped with a FID detector. Mass spectra (MS) were measured on spectrometer by direct inlet at 70 eV. Mass spectroscopy data of the products were collected on an HRMS-TOF instrument or Waters TOFMS GCT Premier using EI or ESI ionization. Melting points were measured with WRR digital point apparatus and not corrected.

2. General Procedures

2.1 General Procedure for the Synthesis of *N*-allylbromoacetamides

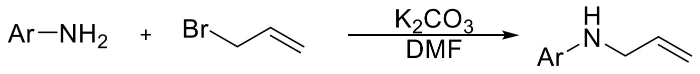

Step I: At 0°C in an ice bath, a solution of appropriate amine Arylamine (10 mmol, 1.0 equiv.) and K_2CO_3 (1.38 g, 10.0 mmol, 1.0 equiv.) in anhydrous DMF (30 ml) slowly added the bromo alkyl ester compounds Allyl bromide (1.50 mL, 11.0 mmol, 1.1 equiv.) dissolved in DMF over 1 hour. Warming the mixture to room temperature and gradually stride over 1 h. Quenched the reaction mixture by addition of saturated NH_4Cl, and the aqueous layer was extracted with EtOAc, dried using Na_2SO_4. Concentrated the organic layers under reduced pressure, and the product was purified by flash column chromatography (petroleum ether : ethyl acetate = 20 : 1 to 10 : 1) to afford the desired product *N*-allylarylamines.

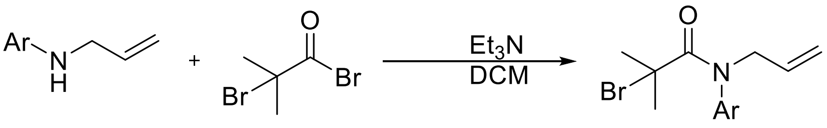

Step II: *N*-allylargonamine and Et_3N (1.40 mL, 10.0 mmol, 2.0 equiv.) were suspended in DCM (20 mL) and cooled to 0 °C, subsequently, dropwise added 2-bromo-2-methylpropanoyl bromide (0.67 mL, 5.5 mmol, 1.1 equiv.) and stirred the reaction system at room temperature for 12 h. After that, NH_4Cl aqueous solution was added, extracted with dichloromethane, washed with saturated sodium bicarbonate, dried with Na_2SO_4. Evaporated the organic layer and purified by column chromatography (petroleum ether : ethyl acetate = 10 : 1 to 5 : 1) gave the *N*-allylbromoacetamides.[1]

2.2 General Procedure for the Synthesis of Products

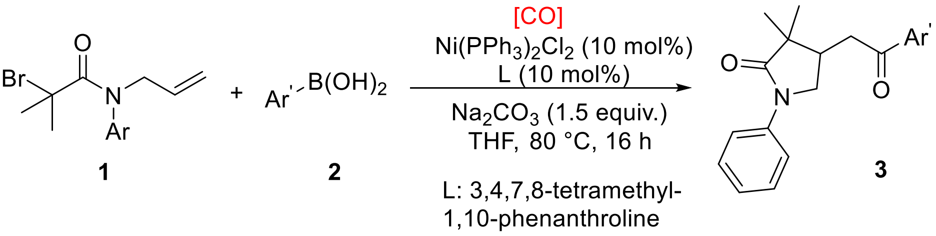

1 (0.2 mmol, 1.0 equiv.), **2** (0.3 mmol, 1.5 equiv.), $Ni(PPh_3)_2Cl_2$ (10 mol%), 2,3,8,9-tetramethyl-1,10-phenanthroline (10 mol%), and Na_2CO_3 (0.3 mmol, 1.5 equiv.) were added to an oven-dried tube (15 mL) which was then placed under vacuum and refilled with nitrogen for three times. Then dry THF (2.0 mL) was added into the tube via a syringe. A mixture of formic acid (2.0 mmol) and acetic anhydride (2.0 mmol) was stirred at 30 °C (oil bath) for 1.5 h, which was then added to a small inner tube with Et_3N (2.0 mmol). The tube was sealed and the mixture was stirred at 80 ℃ (oil bath) for 16 h. After the reaction was completed, the reaction mixture was filtered and concentrated under vacuum. The crude product was purified by column chromatography (petroleum ether : ethyl acetate = 100 : 1 to 5 : 1) on silica gel to afford the corresponding product **3**.

1 mmol scale: **1a** (1 mmol, 1.0 equiv.), **2b** (1.5 mmol, 1.5 equiv.), $Ni(PPh_3)_2Cl_2$ (10 mol%), 2,3,8,9-tetramethyl-1,10-phenanthroline (10 mol%), and Na_2CO_3 (1.5 mmol, 1.5 equiv.) were added to an oven-dried tube (25 mL) which was then placed under vacuum and refilled with nitrogen for three times. Then dry THF (10.0 mL) was added into the tube via a syringe. A mixture of formic acid (10.0 mmol) and acetic anhydride (10.0 mmol) was stirred at 30 °C (oil bath) for 1.5 h, which was then added to a small inner tube with Et_3N (10.0 mmol). The tube was sealed and the mixture was stirred at 80 ℃ (oil bath) for 16 h. After the reaction was completed, the reaction mixture was filtered and concentrated under vacuum. The crude product was purified by column chromatography (petroleum ether : ethyl acetate = 100 : 1 to 5 : 1) on silica gel to afford the corresponding product **3ab** (80% yield; 256.8 mg).

3. Characterization Data of Products

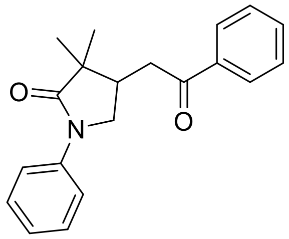

3,3-dimethyl-4-(2-oxo-2-phenylethyl)-1-phenylpyrrolidin-2-one (3aa)

Upon completion the mixture was concentrated and purified via flash column chromatography (petroleum ether : ethyl acetate = 10 : 1, Rf = 0.4) to give the titled product **3aa** as a yellow solid (51.1 mg, 83%).

^{1}H NMR (400 MHz, CDCl$_3$) δ 7.98 (t, 2H), 7.64 (t, *J* = 7.2 Hz, 2H), 7.60 (d, *J* = 7.4 Hz, 1H), 7.50 (t, *J* = 7.6 Hz, 2H), 7.35 (t, 2H), 7.12 (t, *J* = 7.4 Hz, 1H), 4.12 (dd, *J* = 9.8, 7.6 Hz, 1H), 3.44 (t, 1H), 3.29 (dd, *J* = 17.2, 3.5 Hz, 1H), 3.02 (dd, *J* = 17.2, 10.7 Hz, 1H), 2.82 – 2.75 (m, 1H), 1.30 (s, 3H), 1.16 (s, 3H).

^{13}C NMR (101 MHz, CDCl$_3$) δ 198.5, 178.4, 139.6, 136.7, 133.7, 128.9, 128.1, 124.4, 119.7, 51.1, 44.5, 38.7, 37.5, 29.8, 24.1, 19.5.

M.p. 117.6 – 118.3 °C

HRMS (ESI-TOF): m/z: $[M+H]^+$ Calcd. For $C_{20}H_{22}NO_2^+$ 308.1645; Found 308.1647

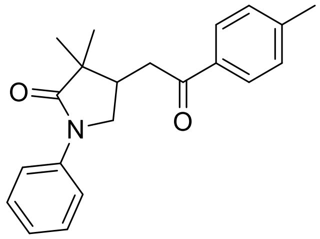

3,3-dimethyl-4-(2-oxo-2-(*p*-tolyl)ethyl)-1-phenylpyrrolidin-2-one (3ab)

Upon completion the mixture was concentrated and purified via flash column chromatography (petroleum ether : ethyl acetate = 10 : 1, Rf = 0.4) to give the titled product **3ab** as a white solid (54.7 mg, 85%).

^{1}H NMR (400 MHz, CDCl$_3$) δ 7.88 (d, *J* = 7.9 Hz, 2H), 7.65 (d, *J* = 8.8 Hz, 2H), 7.34 (t, *J* = 8.1 Hz, 2H), 7.29 (d, *J* = 7.9 Hz, 2H), 7.11 (t, 1H), 4.11 (dd, *J* = 9.8, 7.5 Hz, 1H),

3.44 (t, *J* = 9.3 Hz, 1H), 3.25 (dd, *J* = 17.0, 3.4 Hz, 1H), 2.99 (dd, *J* = 17.0, 10.7 Hz, 1H), 2.83 – 2.73 (m, 1H), 2.43 (s, 3H), 1.30 (s, 3H), 1.15 (s, 3H).

^{13}C NMR (101 MHz, $CDCl_3$) δ 198.0, 178.3, 144.4, 139.5, 134.1, 129.4, 128.7, 128.1, 124.3, 119.6, 50.9, 44.3, 38.6, 37.2, 23.9, 21.6, 19.3.

M. p. 99.0 – 99.6 °C

HRMS (ESI-TOF): m/z: $[M+H]^+$ Calcd. For $C_{21}H_{24}NO_2$ 322.1802; Found 322.1804.

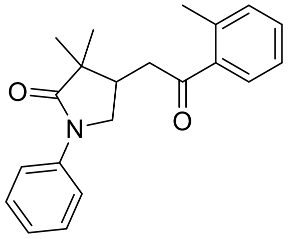

3,3-dimethyl-4-(2-oxo-2-(*o*-tolyl)ethyl)-1-phenylpyrrolidin-2-one (3ac)

Upon completion the mixture was concentrated and purified via flash column chromatography (petroleum ether : ethyl acetate = 10 : 1, Rf = 0.4) to give the titled product **3ac** as a yellow oil (41.2 mg, 64%).

^{1}H NMR (400 MHz, $CDCl_3$) δ 7.69 (d, *J* = 7.7 Hz, 1H), 7.65 (d, *J* = 7.8 Hz, 2H), 7.42 (t, *J* = 7.1 Hz, 1H), 7.36 (t, *J* = 8.0 Hz, 2H), 7.30 (t, *J* = 8.6 Hz, 2H), 7.13 (t, *J* = 7.4 Hz, 1H), 4.12 (t, 1H), 3.46 (t, *J* = 9.3 Hz, 1H), 3.21 (dd, *J* = 17.1, 3.5 Hz, 1H), 2.96 (dd, *J* = 17.1, 10.6 Hz, 1H), 2.79 – 2.71 (m, 1H), 2.53 (s, 3H), 1.30 (s, 3H), 1.13 (s, 3H).

^{13}C NMR (101 MHz, $CDCl_3$) δ 202.2, 178.2, 139.5, 138.4, 137.2, 132.2, 131.8, 128.8, 128.5, 125.8, 124.3, 119.7, 51.0, 44.3, 40.2, 38.8, 23.9, 21.5, 19.3.

HRMS (ESI-TOF): m/z: $[M+H]^+$ Calcd. For $C_{21}H_{24}NO_2$ 322.1802; Found 322.1804.

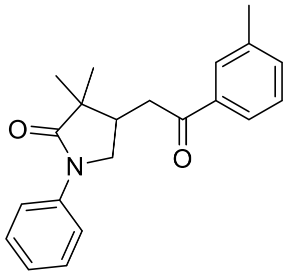

3,3-dimethyl-4-(2-oxo-2-(*m*-tolyl)ethyl)-1-phenylpyrrolidin-2-one (3ad)

Upon completion the mixture was concentrated and purified via flash column chromatography (petroleum ether : ethyl acetate = 10 : 1, Rf = 0.4) to give the titled product **3ad** as a yellow oil (46.3 mg, 72%).

^{1}H NMR (400 MHz, CDCl$_3$) δ 7.78 (d, J = 7.0 Hz, 2H), 7.65 (d, J = 8.5 Hz, 2H), 7.40 (dd, J = 11.3, 7.7 Hz, 2H), 7.34 (t, J = 7.9 Hz, 2H), 7.12 (t, J = 7.4 Hz, 1H), 4.11 (dd, J = 9.8, 7.6 Hz, 1H), 3.43 (t, J = 9.3 Hz, 1H), 3.26 (dd, J = 17.2, 3.4 Hz, 1H), 3.01 (dd, J = 17.2, 10.7 Hz, 1H), 2.81 – 2.74 (m, 1H), 2.43 (s, 3H), 1.30 (s, 3H), 1.16 (s, 3H).

^{13}C NMR (101 MHz, CDCl$_3$) δ 198.6, 178.3, 139.4, 138.6, 136.5, 134.2, 128.7, 128.6, 128.4, 125.2, 124.3, 119.6, 50.9, 44.3, 38.5, 37.3, 23.9, 21.3, 19.3.

HRMS (ESI-TOF): m/z: $[M+H]^+$ Calcd. For $C_{21}H_{24}NO_2$ 322.1802; Found 322.1804.

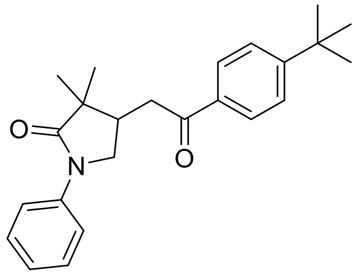

4-(2-(4-(*tert*-butyl)phenyl)-2-oxoethyl)-3,3-dimethyl-1-phenylpyrrolidin-2-one (3ae)

Upon completion the mixture was concentrated and purified via flash column chromatography (petroleum ether : ethyl acetate = 10 : 1, Rf = 0.4) to give the titled product **3ae** as a yellow solid (40.7 mg, 56%).

^{1}H NMR (400 MHz, CDCl$_3$) δ 7.92 (d, J = 8.4 Hz, 2H), 7.65 (d, J = 8.4 Hz, 2H), 7.51 (d, J = 8.4 Hz, 2H), 7.34 (t, J = 7.9 Hz, 2H), 7.12 (t, J = 7.4 Hz, 1H), 4.10 (dd, J = 9.7, 7.6 Hz, 1H), 3.44 (t, J = 9.3 Hz, 1H), 3.27 (dd, J = 16.9, 3.4 Hz, 1H), 2.99 (dd, J = 16.9, 10.7 Hz, 1H), 2.81 – 2.74 (m, 1H), 1.35 (s, 9H), 1.30 (s, 3H), 1.15 (s, 3H).

^{13}C NMR (101 MHz, CDCl$_3$) δ 198.1, 178.3, 157.3, 139.5, 134.0, 128.7, 128.0, 125.7, 124.3, 119.6, 50.9, 44.3, 38.6, 37.2, 35.1, 31.0, 23.9, 19.3.

M.p. 86.1 – 86.7 °C

HRMS (ESI-TOF): m/z: $[M+H]^+$ Calcd. For $C_{24}H_{30}NO_2$ 364.2271; Found 364.2275.

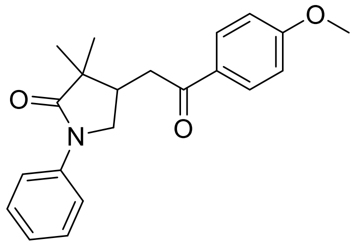

4-(2-(4-methoxyphenyl)-2-oxoethyl)-3,3-dimethyl-1-phenylpyrrolidin-2-one (3af)

Upon completion the mixture was concentrated and purified via flash column chromatography (petroleum ether : ethyl acetate = 10 : 1, Rf = 0.4) to give the titled product **3af** as a yellow solid (56.7 mg, 84%).

^{1}H NMR (400 MHz, CDCl$_3$) δ 7.96 (d, *J* = 8.8 Hz, 2H), 7.65 (d, *J* = 8.3 Hz, 2H), 7.34 (t, *J* = 7.9 Hz, 2H), 7.11 (t, *J* = 7.4 Hz, 1H), 6.96 (d, *J* = 8.8 Hz, 2H), 4.10 (dd, *J* = 9.8, 7.6 Hz, 1H), 3.88 (s, 3H), 3.43 (t, *J* = 9.3 Hz, 1H), 3.22 (dd, *J* = 16.8, 3.4 Hz, 1H), 2.96 (dd, *J* = 16.8, 10.7 Hz, 1H), 2.80 – 2.72 (m, 1H), 1.29 (s, 3H), 1.15 (s, 3H).

^{13}C NMR (101 MHz, CDCl$_3$) δ 196.9, 178.3, 163.7, 139.5, 130.3, 129.6, 128.7, 124.2, 119.5, 113.8, 55.5, 50.9, 44.3, 38.6, 36.9, 23.9, 19.2.

M.p. 98.1 – 98.5 °C

HRMS (ESI-TOF): m/z: $[M+H]^+$ Calcd. For $C_{21}H_{24}NO_3$ 338.1751; Found 338.1754.

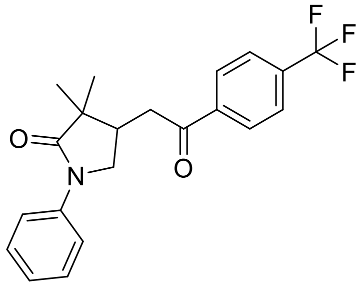

3,3-dimethyl-4-(2-oxo-2-(4-(trifluoromethoxy)phenyl)ethyl)-1-phenylpyrrolidin-2-one (3ag)

Upon completion the mixture was concentrated and purified via flash column chromatography (petroleum ether : ethyl acetate = 10 : 1, Rf = 0.5) to give the titled product **3ag** as a yellow solid (27.4 mg, 35%).

^{1}H NMR (400 MHz, CDCl$_3$) δ 8.04 (d, *J* = 8.8 Hz, 2H), 7.65 (d, *J* = 7.8 Hz, 2H), 7.34 (dd, *J* = 15.6, 7.4 Hz, 4H), 7.13 (t, *J* = 7.4 Hz, 1H), 4.13 (dd, *J* = 9.8, 7.5 Hz, 1H), 3.43

(t, 1H), 3.27 (dd, *J* = 17.3, 3.4 Hz, 1H), 3.01 (dd, *J* = 17.3, 10.6 Hz, 1H), 2.82 – 2.75 (m, 1H), 1.31 (s, 3H), 1.16 (s, 3H).

^{13}C NMR (101 MHz, CDCl$_3$) δ 196.8, 178.1, 152.9, 139.4, 134.7, 130.1, 128.8, 124.4, 120.5, 120.24 (d, *J* = 259.1 Hz).119.6, 50.9, 44.3, 38.5, 37.5, 24.0, 19.3.

^{19}F NMR (377 MHz, CDCl$_3$) δ -57.58 (s).

HRMS (ESI-TOF): m/z: $[M+H]^+$ Calcd. For $C_{21}H_{21}F_3NO_3$ 392.1468; Found 392.1471.

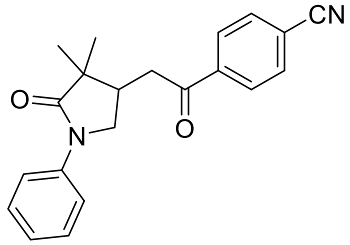

4-(2-(4,4-dimethyl-5-oxo-1-phenylpyrrolidin-3-yl)acetyl)benzonitrile (3ah)

Upon completion the mixture was concentrated and purified via flash column chromatography (petroleum ether : ethyl acetate = 10 : 1, Rf = 0.4) to give the titled product **3ah** as a yellow oil (26.6mg, 40%).

^{1}H NMR (400 MHz, CDCl$_3$) δ 8.08 (d, *J* = 8.2 Hz, 2H), 7.81 (d, *J* = 8.1 Hz, 2H), 7.64 (d, *J* = 8.3 Hz, 2H), 7.36 (t, *J* = 7.8 Hz, 2H), 7.14 (t, *J* = 7.2 Hz, 1H), 4.14 (dd, *J* = 9.6, 7.8 Hz, 1H), 3.43 (t, *J* = 9.2 Hz, 1H), 3.29 (dd, *J* = 17.7, 3.2 Hz, 1H), 3.04 (dd, *J* = 17.7, 10.6 Hz, 1H), 2.83 – 2.75 (m, 1H), 1.31 (s, 3H), 1.16 (s, 3H).

^{13}C NMR (101 MHz, CDCl$_3$) δ 197.0, 177.9, 139.3, 132.7, 128.8, 128.4, 124.5, 119.6, 117.7, 116.8, 50.8, 44.2, 38.3, 37.8, 24.0, 19.4.

HRMS (ESI-TOF): m/z: $[M+H]^+$ Calcd. For $C_{21}H_{21}N_2O_2$ 333.1598; Found 333.1601.

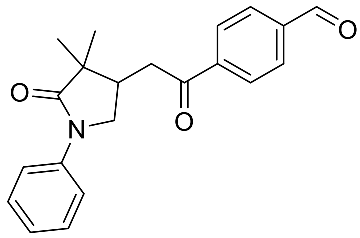

4-(2-(4,4-dimethyl-5-oxo-1-phenylpyrrolidin-3-yl)acetyl)benzaldehyde (3ai)

Upon completion the mixture was concentrated and purified via flash column chromatography (petroleum ether : ethyl acetate = 10 : 1, Rf = 0.4) to give the titled product **3ai** as a yellow oil (51.0mg, 76%).

^{1}H NMR (400 MHz, CDCl$_3$) δ 10.13 (s, 1H), 8.13 (d, J = 8.2 Hz, 2H), 8.01 (d, J = 8.2 Hz, 2H), 7.65 (d, J = 7.9 Hz, 2H), 7.36 (t, J = 8.0 Hz, 2H), 7.13 (t, J = 7.4 Hz, 1H), 4.15 (dd, J = 9.8, 7.6 Hz, 1H), 3.45 (t, J = 9.2 Hz, 1H), 3.32 (dd, J = 17.6, 3.4 Hz, 1H), 3.07 (dd, J = 17.6, 10.6 Hz, 1H), 2.84 – 2.77 (m, 1H), 1.31 (s, 3H), 1.17 (s, 3H).

^{13}C NMR (101 MHz, CDCl$_3$) δ 197.8, 191.4, 178.0, 140.6, 139.4, 139.3, 129.9, 128.8, 128.5, 124.4, 119.7, 50.9, 44.3, 38.4, 38.0, 24.0, 19.4.

HRMS (ESI-TOF): m/z: $[M+H]^+$ Calcd. For $C_{21}H_{22}NO_3$ 336.1594; Found 336.1597.

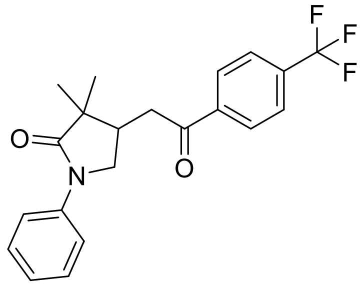

3,3-dimethyl-4-(2-oxo-2-(4-(trifluoromethyl)phenyl)ethyl)-1-phenylpyrrolidin-2-one (3aj)

Upon completion the mixture was concentrated and purified via flash column chromatography (petroleum ether : ethyl acetate = 10 : 1, Rf = 0.4) to give the titled product **3aj** as a yellow oil (45.1mg, 60%).

^{1}H NMR (400 MHz, CDCl$_3$) δ 8.09 (d, J = 8.0 Hz, 2H), 7.77 (d, J = 8.1 Hz, 2H), 7.65 (d, J = 8.4 Hz, 2H), 7.35 (t, J = 7.6 Hz, 2H), 7.13 (t, J = 7.4 Hz, 1H), 4.14 (t, 1H), 3.44 (t, J = 9.2 Hz, 1H), 3.31 (dd, J = 17.5, 3.3 Hz, 1H), 3.05 (dd, J = 17.5, 10.6 Hz, 1H), 2.83 – 2.76 (m, 1H), 1.31 (s, 3H), 1.16 (s, 3H).

^{13}C NMR (101 MHz, CDCl$_3$) δ 197.4, 178.0, 139.4, 139.1, 134.78 (d, J = 32.9 Hz), 128.8, 128.3, 127.5, 125.86 (d, J = 3.6 Hz), 124.4, 123.45 (d, J = 272.8 Hz), 119.6, 50.8, 44.3, 38.4, 37.7, 24.0, 19.3.

^{19}F NMR (377 MHz, CDCl$_3$) δ -63.12 (s).

HRMS (ESI-TOF): m/z: $[M+H]^+$ Calcd. For $C_{21}H_{21}F_3NO_2$ 376.1519; Found 376.1522.

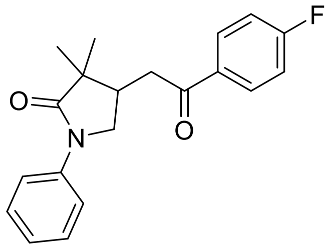

4-(2-(4-fluorophenyl)-2-oxoethyl)-3,3-dimethyl-1-phenylpyrrolidin-2-one (3ak)

Upon completion the mixture was concentrated and purified via flash column chromatography (petroleum ether : ethyl acetate = 10 : 1, Rf = 0.4) to give the titled product **3ak** as a yellow oil (56.7mg, 87%).

^{1}H NMR (400 MHz, CDCl$_3$) δ 7.84 (d, J = 8.6 Hz, 2H), 7.64 (d, J = 8.2 Hz, 4H), 7.35 (t, J = 8.0 Hz, 2H), 7.13 (t, J = 7.4 Hz, 1H), 4.12 (dd, J = 9.8, 7.5 Hz, 1H), 3.43 (t, 1H), 3.24 (dd, J = 17.3, 3.4 Hz, 1H), 2.99 (dd, J = 17.3, 10.6 Hz, 1H), 2.81 – 2.73 (m, 1H), 1.30 (s, 3H), 1.15 (s, 3H).

^{13}C NMR (101 MHz, CDCl$_3$) δ 196.7, 178.1, 165.90 (d, J = 255.6 Hz), 139.5, 132.98 (d, J = 2.8 Hz), 130.7, 130.6, 128.7, 124.3, 119.6, 115.88 (d, J = 21.9 Hz), 50.9, 44.3, 38.5, 37.3, 23.9, 19.3.

^{19}F NMR (377 MHz, CDCl$_3$) δ -104.24 (d, J = 4.7 Hz).

HRMS (ESI-TOF): m/z: $[M+H]^+$ Calcd. For $C_{20}H_{21}FNO_2$ 326.1551; Found 326.1553.

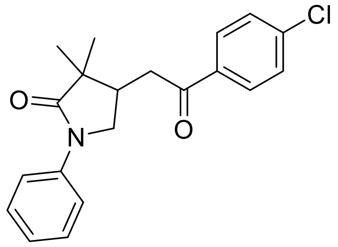

4-(2-(4-chlorophenyl)-2-oxoethyl)-3,3-dimethyl-1-phenylpyrrolidin-2-one (3al)

Upon completion the mixture was concentrated and purified via flash column chromatography (petroleum ether : ethyl acetate = 10 : 1, Rf = 0.4) to give the titled product **3al** as a yellow oil (17.1mg, 25%).

^{1}H NMR (400 MHz, CDCl$_3$) δ 7.93 (d, J = 8.6 Hz, 2H), 7.65 (d, J = 7.9 Hz, 2H), 7.47 (d, J = 8.5 Hz, 2H), 7.35 (t, J = 8.0 Hz, 2H), 7.13 (t, J = 7.4 Hz, 1H), 4.13 (dd, J = 9.8,

7.6 Hz, 1H), 3.43 (t, 1H), 3.25 (dd, J = 17.3, 3.4 Hz, 1H), 2.99 (dd, J = 17.3, 10.6 Hz, 1H), 2.81 – 2.74 (m, 1H), 1.30 (s, 3H), 1.15 (s, 3H).

^{13}C NMR (101 MHz, $CDCl_3$) δ 197.1, 178.1, 140.0, 139.5, 134.8, 129.4, 129.1, 128.8, 124.4, 119.6, 50.9, 44.3, 38.5, 37.4, 29.7, 24.0, 19.3.

HRMS (ESI-TOF): m/z: $[M+H]^+$ Calcd. For $C_{20}H_{21}ClNO_2$ 342.1255; Found 342.1258.

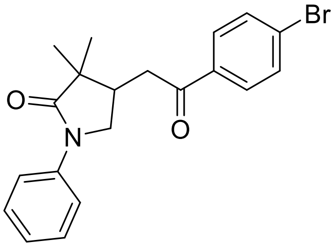

4-(2-(4-bromophenyl)-2-oxoethyl)-3,3-dimethyl-1-phenylpyrrolidin-2-one (3am)

Upon completion the mixture was concentrated and purified via flash column chromatography (petroleum ether : ethyl acetate = 10 : 1, Rf = 0.4) to give the titled product **3am** as a yellow oil (27.1mg, 35%).

^{1}H NMR (400 MHz, $CDCl_3$) δ 7.84 (d, J = 8.6 Hz, 2H), 7.64 (d, J = 8.2 Hz, 4H), 7.35 (t, J = 8.0 Hz, 2H), 7.13 (t, J = 7.4 Hz, 1H), 4.12 (dd, J = 9.8, 7.5 Hz, 1H), 3.43 (t, 1H), 3.24 (dd, J = 17.3, 3.4 Hz, 1H), 2.99 (dd, J = 17.3, 10.6 Hz, 1H), 2.81 – 2.73 (m, 1H), 1.30 (s, 3H), 1.15 (s, 3H).

^{13}C NMR (101 MHz, $CDCl_3$) δ 197.3, 178.1, 139.4, 135.2, 132.1, 129.5, 128.8, 124.4, 119.6, 50.9, 44.3, 38.5, 37.4, 24.0, 19.3.

HRMS (ESI-TOF): m/z: $[M+H]^+$ Calcd. For $C_{20}H_{21}BrNO_2$ 386.0750; Found 386.0753.

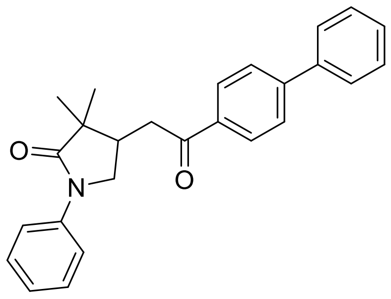

5-(2-([1,1'-biphenyl]-4-yl)-2-oxoethyl)-3,3-dimethyl-1-phenylpyrrolidin-2-one (3an)

Upon completion the mixture was concentrated and purified via flash column chromatography (petroleum ether : ethyl acetate = 10 : 1, Rf = 0.4) to give the titled product **3an** as a yellow solid (49.1mg, 64%).

^{1}H NMR (400 MHz, $CDCl_3$) δ 8.07 (d, *J* = 8.3 Hz, 2H), 7.73 (d, *J* = 8.3 Hz, 2H), 7.66 (t, *J* = 8.5 Hz, 4H), 7.49 (t, *J* = 7.4 Hz, 2H), 7.42 (t, *J* = 7.3 Hz, 1H), 7.35 (d, *J* = 8.2 Hz, 2H), 7.13 (t, *J* = 7.4 Hz, 1H), 4.15 (dd, *J* = 9.7, 7.7 Hz, 1H), 3.47 (t, *J* = 9.3 Hz, 1H), 3.32 (dd, *J* = 17.1, 3.4 Hz, 1H), 3.06 (dd, *J* = 17.1, 10.7 Hz, 1H), 2.85 – 2.78 (m, 1H), 1.33 (s, 3H), 1.18 (s, 3H).

^{13}C NMR (101 MHz, $CDCl_3$) δ 198.0, 178.2, 146.2, 139.6, 139.5, 135.2, 129.0, 128.8, 128.6, 128.4, 127.4, 127.2, 124.3, 119.6, 50.9, 44.3, 38.6, 37.4, 24.0, 19.3.

M.p. 77.0 – 77.6 ℃

HRMS (ESI-TOF): m/z: $[M+H]^+$ Calcd. For $C_{26}H_{26}NO_2$ 384.1958; Found 384.1963.

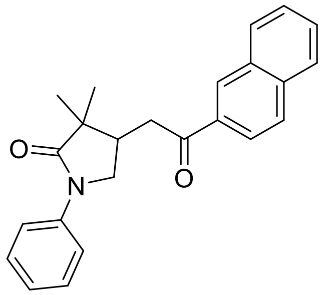

3,3-dimethyl-4-(2-(naphthalen-2-yl)-2-oxoethyl)-1-phenylpyrrolidin-2-one (3ao)

Upon completion the mixture was concentrated and purified via flash column chromatography (petroleum ether : ethyl acetate = 10 : 1, Rf = 0.4) to give the titled product **3ao** as a yellow solid (58.7 mg, 82%).

^{1}H NMR (400 MHz, CDCl$_3$) δ 8.50 (s, 1H), 8.02 (dd, J = 22.4, 8.3 Hz, 2H), 7.92 (dd, J = 12.0, 8.6 Hz, 2H), 7.68 – 7.57 (m, 4H), 7.35 (t, J = 7.6 Hz, 2H), 7.13 (t, J = 7.4 Hz, 1H), 4.17 (t, J = 8.6 Hz, 1H), 3.49 (t, J = 9.3 Hz, 1H), 3.42 (dd, J = 17.2, 2.6 Hz, 1H), 3.17 (dd, J = 17.1, 10.7 Hz, 1H), 2.89 – 2.82 (m, 1H), 1.34 (s, 3H), 1.21 (s, 3H).

^{13}C NMR (101 MHz, CDCl$_3$) δ 198.3, 178.2, 139.5, 135.7, 133.8, 132.4, 129.7, 129.5, 128.7, 128.7, 127.8, 127.0, 124.3, 123.5, 119.6, 51.0, 44.4, 38.6, 37.4, 24.0, 19.4.

M.p. 103.4 – 104.0 °C

HRMS (ESI-TOF): m/z: $[M+H]^+$ Calcd. For $C_{24}H_{24}NO_2$ 358.1802; Found 358.1806.

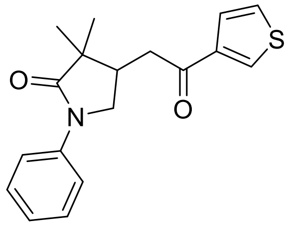

3,3-dimethyl-4-(2-oxo-2-(thiophen-3-yl)ethyl)-1-phenylpyrrolidin-2-one (3ap)

Upon completion the mixture was concentrated and purified via flash column chromatography (petroleum ether : ethyl acetate = 10 : 1, Rf = 0.4) to give the titled product **3ap** as a colourless oil (28.9 mg, 46%).

^{1}H NMR (400 MHz, CDCl$_3$) δ 8.10 (t, 1H), 7.64 (d, J = 8.5 Hz, 2H), 7.57 (d, J = 5.1 Hz, 1H), 7.37 – 7.33 (m, 3H), 7.12 (t, J = 7.4 Hz, 1H), 4.10 (dd, J = 9.8, 7.6 Hz, 1H), 3.45 (t, J = 9.3 Hz, 1H), 3.18 (dd, J = 16.8, 3.4 Hz, 1H), 2.93 (dd, J = 16.8, 10.7 Hz, 1H), 2.80 – 2.72 (m, 1H), 1.30 (s, 3H), 1.14 (s, 3H).

^{13}C NMR (101 MHz, CDCl$_3$) δ 192.7, 178.2, 141.9, 139.5, 132.2, 128.8, 126.8, 126.7, 124.3, 119.6, 50.9, 44.3, 38.6, 38.5, 23.9, 19.3.

HRMS (ESI-TOF): m/z: $[M+H]^+$ Calcd. For $C_{18}H_{20}NO_2S$ 314.1209; Found 314.1216.

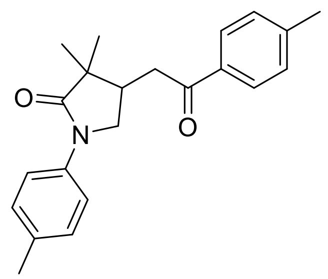

3,3-dimethyl-4-(2-oxo-2-(*p*-tolyl)ethyl)-1-(*p*-tolyl)pyrrolidin-2-one (3bb)

Upon completion the mixture was concentrated and purified via flash column chromatography (petroleum ether : ethyl acetate = 10 : 1, Rf = 0.4) to give the titled product **3bb** as a yellow oil (57.7 mg, 86%).

^{1}H NMR (400 MHz, $CDCl_3$) δ 7.88 (d, J = 8.1 Hz, 2H), 7.52 (d, J = 8.4 Hz, 2H), 7.28 (d, J = 8.1 Hz, 2H), 7.14 (d, J = 8.4 Hz, 2H), 4.07 (dd, J = 9.7, 7.6 Hz, 1H), 3.41 (t, J = 9.3 Hz, 1H), 3.24 (dd, J = 17.0, 3.4 Hz, 1H), 2.98 (dd, J = 17.0, 10.7 Hz, 1H), 2.79 – 2.72 (m, 1H), 2.42 (s, 3H), 2.31 (s, 3H), 1.29 (s, 3H), 1.14 (s, 3H).

^{13}C NMR (101 MHz, $CDCl_3$) δ 198.0, 178.0, 144.3, 137.0, 134.0, 133.8, 129.4, 129.2, 128.0, 119.6, 51.0, 44.2, 38.6, 37.2, 23.9, 21.6, 20.7, 19.2.

HRMS (ESI-TOF): m/z: $[M+H]^+$ Calcd. For $C_{22}H_{26}NO_2$ 336.1958; Found 336.1961.

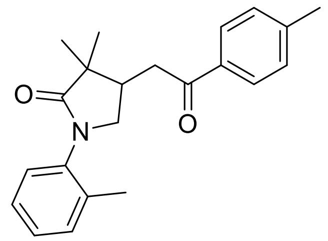

3,3-dimethyl-4-(2-oxo-2-phenylethyl)-1-(*o*-tolyl)pyrrolidin-2-one (3bc)

Upon completion the mixture was concentrated and purified via flash column chromatography (petroleum ether : ethyl acetate = 10 : 1, Rf = 0.4) to give the titled product **3bc** as a yellow oil (54.3 mg, 81%).

^{1}H NMR (400 MHz, $CDCl_3$) δ 7.87 (d, J = 8.1 Hz, 2H), 7.28 (d, J = 8.0 Hz, 2H), 7.23 (t, 1H), 7.19 (t, 2H), 7.10 (t, 1H), 3.90 (dd, J = 10.0, 7.5 Hz, 1H), 3.34 (t, J = 9.4 Hz, 1H), 3.25 (dd, J = 16.9, 3.4 Hz, 1H), 3.01 (dd, J = 16.8, 10.6 Hz, 1H), 2.90 – 2.82 (m, 1H), 2.42 (s, 3H), 2.21 (s, 3H), 1.30 (s, 3H), 1.21 (s, 3H).

^{13}C NMR (101 MHz, $CDCl_3$) δ 198.0, 178.0, 144.3, 137.3, 135.4, 134.1, 131.0, 129.4, 128.0, 127.6, 126.7, 126.6, 53.1, 43.1, 40.0, 37.1, 23.8, 21.6, 19.2, 17.9.

HRMS (ESI-TOF): m/z: $[M+H]^+$ Calcd. For $C_{22}H_{26}NO_2$ 336.1958; Found 336.1961.

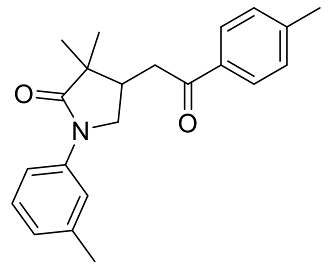

3,3-dimethyl-4-(2-oxo-2-(*p*-tolyl)ethyl)-1-(*m*-tolyl)pyrrolidin-2-one (3bd)

Upon completion the mixture was concentrated and purified via flash column chromatography (petroleum ether : ethyl acetate = 10 : 1, Rf = 0.4) to give the titled product **3bd** as a yellow oil (53.0 mg, 79%).

^{1}H NMR (400 MHz, CDCl$_3$) δ 7.88 (d, *J* = 8.2 Hz, 2H), 7.51 (s, 1H), 7.43 (d, *J* = 8.3 Hz, 1H), 7.29 (d, *J* = 8.0 Hz, 2H), 7.22 (t, *J* = 7.8 Hz, 1H), 6.94 (d, *J* = 7.5 Hz, 1H), 4.10 (dd, *J* = 9.8, 7.6 Hz, 1H), 3.43 (t, *J* = 9.3 Hz, 1H), 3.25 (dd, *J* = 17.0, 3.4 Hz, 1H), 2.99 (dd, *J* = 17.0, 10.7 Hz, 1H), 2.80 – 2.72 (m, 1H), 2.43 (s, 3H), 2.34 (s, 3H), 1.30 (s, 3H), 1.15 (s, 3H).

^{13}C NMR (101 MHz, CDCl$_3$) δ 198.1, 178.2, 144.4, 139.4, 138.5, 134.0, 129.4, 128.5, 128.1, 125.1, 120.3, 116.7, 51.0, 44.3, 38.6, 37.2, 23.9, 21.6, 21.5, 19.2.

HRMS (ESI-TOF): m/z: $[M+H]^+$ Calcd. For $C_{22}H_{26}NO_2$ 336.1958; Found 336.1961.

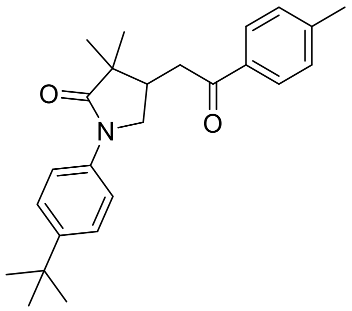

1-(4-*(tert*-butyl)phenyl)-3,3-dimethyl-4-(2-oxo-2-(*p*-tolyl)ethyl)pyrrolidin-2-one (3be)

Upon completion the mixture was concentrated and purified via flash column chromatography (petroleum ether : ethyl acetate = 10 : 1, Rf = 0.4) to give the titled product **3be** as a yellow solid (69.5 mg, 92%).

^{1}H NMR (400 MHz, CDCl$_3$) δ 7.88 (d, J = 8.2 Hz, 2H), 7.58 (d, J = 8.8 Hz, 2H), 7.36 (d, J = 8.8 Hz, 2H), 7.29 (d, J = 8.1 Hz, 2H), 4.10 (dd, J = 9.8, 7.5 Hz, 1H), 3.42 (t, 1H), 3.25 (dd, J = 17.0, 3.5 Hz, 1H), 2.99 (dd, J = 17.0, 10.7 Hz, 1H), 2.80 – 2.73 (m, 1H), 2.43 (s, 3H), 1.30 (s, 9H), 1.29 (s, 3H), 1.14 (s, 3H).

^{13}C NMR (101 MHz, CDCl$_3$) δ 198.1, 178.1, 147.1, 144.3, 136.9, 134.1, 129.4, 128.1, 125.5, 119.2, 50.9, 44.2, 38.6, 37.2, 34.3, 31.3, 23.9, 21.6, 19.3.

HRMS (ESI-TOF): m/z: $[M+H]^+$ Calcd. For $C_{25}H_{32}NO_2$ 378.2428; Found 378.2432.

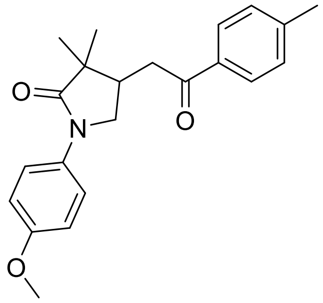

1-(4-methoxyphenyl)-3,3-dimethyl-4-(2-oxo-2-(*p*-tolyl)ethyl)pyrrolidin-2-one (3bf)

Upon completion the mixture was concentrated and purified via flash column chromatography (petroleum ether : ethyl acetate = 10 : 1, Rf = 0.4) to give the titled product **3bf** as a yellow solid (54.2 mg, 77%).

^{1}H NMR (400 MHz, CDCl$_3$) δ 7.87 (d, J = 8.1 Hz, 2H), 7.53 (d, J = 9.0 Hz, 2H), 7.28 (d, J = 8.1 Hz, 2H), 6.86 (d, J = 9.0 Hz, 2H), 4.04 (dd, J = 9.6, 7.7 Hz, 1H), 3.77 (s, 3H), 3.39 (t, J = 9.3 Hz, 1H), 3.24 (dd, J = 17.0, 3.4 Hz, 1H), 2.97 (dd, J = 17.0, 10.7 Hz, 1H), 2.78 – 2.71 (m, 1H), 2.42 (s, 3H), 1.28 (s, 3H), 1.13 (s, 3H).

^{13}C NMR (101 MHz, CDCl$_3$) δ 198.1, 177.8, 156.3, 144.3, 134.0, 132.7, 129.4, 128.0, 121.2, 113.9, 55.4, 51.3, 44.0, 38.7, 37.1, 23.9, 21.6, 19.2.

HRMS (ESI-TOF): m/z: $[M+H]^+$ Calcd. For $C_{22}H_{26}NO_3$ 352.1907; Found 352.1911.

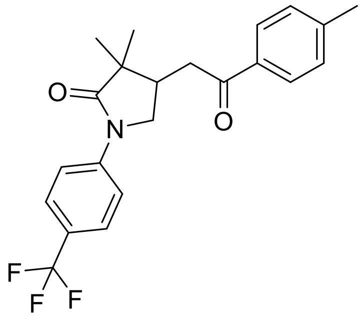

3,3-dimethyl-4-(2-oxo-2-(*p*-tolyl)ethyl)-1-(4-(trifluoromethyl)phenyl)pyrrolidin-2-one (3bg)

Upon completion the mixture was concentrated and purified via flash column chromatography (petroleum ether : ethyl acetate = 10 : 1, Rf = 0.4) to give the titled product **3bg** as a yellow oil (42.9 mg, 55%).

^{1}H NMR (400 MHz, CDCl$_3$) δ 7.89 (d, *J* = 8.1 Hz, 2H), 7.80 (d, *J* = 8.6 Hz, 2H), 7.60 (d, *J* = 8.7 Hz, 2H), 7.30 (d, *J* = 8.1 Hz, 2H), 4.16 (dd, *J* = 9.7, 7.7 Hz, 1H), 3.44 (t, *J* = 9.4 Hz, 1H), 3.28 (dd, *J* = 17.1, 3.2 Hz, 1H), 3.00 (dd, *J* = 17.2, 10.8 Hz, 1H), 2.83 – 2.75 (m, 1H), 2.43 (s, 3H), 1.31 (s, 3H), 1.16 (s, 3H).

^{13}C NMR (101 MHz, CDCl$_3$) δ 197.9, 178.8, 144.6, 142.4, 134.0, 129.5, 128.1, 125.93 (q, *J* = 3.7 Hz), 124.09 (q, *J* = 271.5 Hz), 119.0, 50.7, 44.5, 38.4, 37.1, 23.8, 21.7, 19.4.

^{19}F NMR (377 MHz, CDCl$_3$) δ -62.07 (s).

HRMS (ESI-TOF): m/z: $[M+H]^+$ Calcd. For $C_{22}H_{23}F_3NO_2$ 390.1675; Found 390.1679.

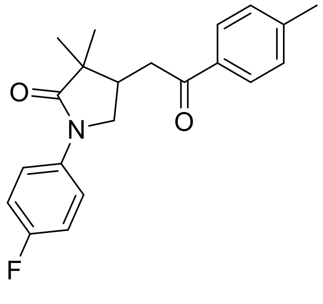

1-(4-fluorophenyl)-3,3-dimethyl-4-(2-oxo-2-(*p*-tolyl)ethyl)pyrrolidin-2-one (3bh)

Upon completion the mixture was concentrated and purified via flash column chromatography (petroleum ether : ethyl acetate = 10 : 1, Rf = 0.4) to give the titled product **3bh** as a yellow oil (54.3 mg, 80%).

¹H NMR (400 MHz, CDCl₃) *δ* 7.87 (d, *J* = 8.2 Hz, 2H), 7.58 (dd, *J* = 9.1, 4.8 Hz, 2H), 7.27 (d, *J* = 8.1 Hz, 2H), 7.00 (t, *J* = 8.7 Hz, 2H), 4.06 (dd, *J* = 9.7, 7.6 Hz, 1H), 3.40 (t, *J* = 9.3 Hz, 1H), 3.24 (dd, *J* = 17.1, 3.4 Hz, 1H), 2.97 (dd, *J* = 17.1, 10.8 Hz, 1H), 2.78 – 2.71 (m, 1H), 2.41 (s, 3H), 1.28 (s, 3H), 1.13 (s, 3H).

¹³C NMR (101 MHz, CDCl₃) *δ* 197.9, 178.1, 159.20 (d, *J* = 243.8 Hz),, 144.4, 135.6, 135.5, 134.0, 129.4, 128.0, 121.23 (d, *J* = 7.9 Hz), 115.28 (d, *J* = 22.3 Hz), 51.1, 44.1, 38.5, 37.0, 23.8, 21.6, 19.2.

¹⁹F NMR (377 MHz, CDCl₃) *δ* -118.02 (d, *J* = 4.5 Hz).

HRMS (ESI-TOF): m/z: $[M+H]^+$ Calcd. For $C_{21}H_{23}FNO_2$ 340.1707; Found 340.1710.

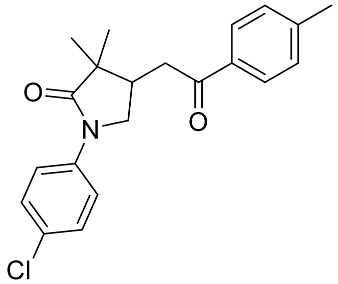

1-(4-chlorophenyl)-3,3-dimethyl-4-(2-oxo-2-(*p*-tolyl)ethyl)pyrrolidin-2-one (3bi)

Upon completion the mixture was concentrated and purified via flash column chromatography (petroleum ether : ethyl acetate = 10 : 1, Rf = 0.4) to give the titled product **3bi** as a yellow oil (43.5 mg, 61%).

¹H NMR (400 MHz, CDCl₃) *δ* 7.88 (d, *J* = 8.1 Hz, 2H), 7.61 (d, *J* = 8.9 Hz, 2H), 7.29 (d, *J* = 9.0 Hz, 4H), 4.09 (dd, *J* = 9.5, 7.8 Hz, 1H), 3.40 (t, *J* = 9.3 Hz, 1H), 3.26 (dd, *J* = 17.1, 3.2 Hz, 1H), 2.98 (dd, *J* = 17.1, 10.8 Hz, 1H), 2.80 – 2.72 (m, 1H), 2.43 (s, 3H), 1.29 (s, 3H), 1.14 (s, 3H).

¹³C NMR (101 MHz, CDCl₃) *δ* 197.9, 178.3, 144.5, 138.1, 134.0, 129.4, 129.2, 128.7, 128.1, 120.6, 50.9, 44.3, 38.5, 37.1, 23.9, 21.7, 19.3.

HRMS (ESI-TOF): m/z: $[M+H]^+$ Calcd. For $C_{21}H_{23}ClNO_2$ 356.1412; Found 356.1415.

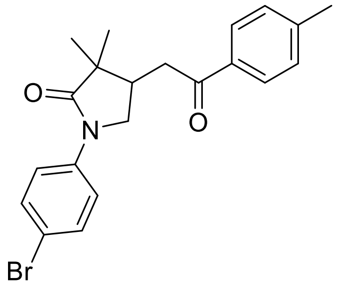

1-(4-bromophenyl)-3,3-dimethyl-4-(2-oxo-2-(*p*-tolyl)ethyl)pyrrolidin-2-one (3bj)

Upon completion the mixture was concentrated and purified via flash column chromatography (petroleum ether : ethyl acetate = 10 : 1, Rf = 0.4) to give the titled product **3bj** as a yellow oil (56.1 mg, 70%).

^{1}H NMR (400 MHz, CDCl$_3$) δ 7.87 (d, *J* = 8.2 Hz, 2H), 7.56 (d, *J* = 9.0 Hz, 2H), 7.43 (d, *J* = 9.0 Hz, 2H), 7.29 (d, *J* = 8.0 Hz, 2H), 4.08 (dd, *J* = 9.7, 7.6 Hz, 1H), 3.39 (t, *J* = 9.3 Hz, 1H), 3.25 (dd, *J* = 17.1, 3.3 Hz, 1H), 2.98 (dd, *J* = 17.1, 10.8 Hz, 1H), 2.79 – 2.73 (m, 1H), 2.43 (s, 3H), 1.29 (s, 3H), 1.14 (s, 3H).

^{13}C NMR (101 MHz, CDCl$_3$) δ 197.9, 178.4, 144.5, 138.5, 134.0, 131.6, 129.4, 128.1, 120.9, 116.9, 50.8, 44.3, 38.4, 37.1, 23.8, 21.7, 19.3.

HRMS (ESI-TOF): m/z: $[M+H]^+$ Calcd. For $C_{21}H_{23}BrNO_2$ 400.0907; Found 400.0910.

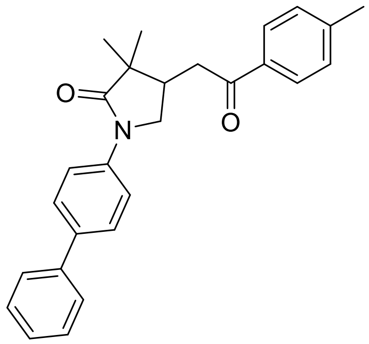

1-([1,1'-biphenyl]-4-yl)-3,3-dimethyl-4-(2-oxo-2-(*p*-tolyl)ethyl)pyrrolidin-2-one (3bk)

Upon completion the mixture was concentrated and purified via flash column chromatography (petroleum ether : ethyl acetate = 10 : 1, Rf = 0.4) to give the titled product **3bk** as a White solid (43.8 mg, 55%).

^{1}H NMR (400 MHz, CDCl$_3$) δ 7.90 (d, *J* = 8.1 Hz, 2H), 7.74 (d, *J* = 8.7 Hz, 2H), 7.58 (dd, *J* = 8.0, 4.0 Hz, 4H), 7.43 (t, *J* = 7.6 Hz, 2H), 7.32 (dd, *J* = 13.6, 7.6 Hz, 3H), 4.17

(dd, *J* = 9.7, 7.6 Hz, 1H), 3.47 (t, *J* = 9.3 Hz, 1H), 3.27 (dd, *J* = 17.0, 3.4 Hz, 1H), 3.01 (dd, *J* = 17.0, 10.7 Hz, 1H), 2.84 – 2.76 (m, 1H), 2.44 (s, 3H), 1.32 (s, 3H), 1.17 (s, 3H).

^{13}C NMR (101 MHz, CDCl$_3$) δ 198.1, 178.4, 144.5, 140.5, 138.8, 137.0, 134.1, 129.5, 128.7, 128.1, 127.4, 127.1, 126.9, 119.8, 51.0, 44.4, 38.6, 37.2, 29.7, 24.0, 21.7, 19.4.

M.p. 127.3 – 127.8 °C

HRMS (ESI-TOF): m/z: [M+H]$^+$ Calcd. For $C_{27}H_{28}NO_2$ 398.2115; Found 398.2120.

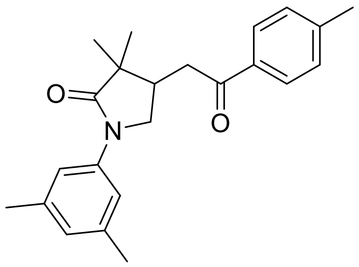

1-(3,5-dimethylphenyl)-3,3-dimethyl-4-(2-oxo-2-(*p*-tolyl)ethyl)pyrrolidin-2-one (3bl)

Upon completion the mixture was concentrated and purified via flash column chromatography (petroleum ether : ethyl acetate = 10 : 1, Rf = 0.4) to give the titled product **3bl** as a yellow oil (54.5 mg, 78%).

^{1}H NMR (400 MHz, CDCl$_3$) δ 7.88 (d, *J* = 8.1 Hz, 2H), 7.29 (d, *J* = 4.6 Hz, 4H), 6.77 (s, 1H), 4.09 (dd, *J* = 9.8, 7.6 Hz, 1H), 3.42 (t, *J* = 9.3 Hz, 1H), 3.25 (dd, *J* = 17.0, 3.4 Hz, 1H), 2.98 (dd, *J* = 17.0, 10.7 Hz, 1H), 2.79 – 2.71 (m, 1H), 2.43 (s, 3H), 2.30 (s, 6H), 1.29 (s, 3H), 1.14 (s, 3H).

^{13}C NMR (101 MHz, CDCl$_3$) δ 198.1, 178.2, 144.4, 139.4, 138.3, 134.1, 129.4, 128.1, 126.0, 117.4, 51.1, 44.3, 38.6, 37.2, 23.9, 21.6, 21.4, 19.3.

HRMS (ESI-TOF): m/z: [M+H]$^+$ Calcd. For $C_{23}H_{28}NO_2$ 350.2115; Found 350.2118.

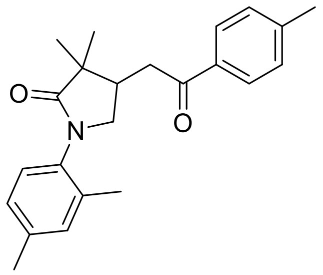

1-(2,4-dimethylphenyl)-3,3-dimethyl-4-(2-oxo-2-(*p*-tolyl)ethyl)pyrrolidin-2-one (3bm)

Upon completion the mixture was concentrated and purified via flash column chromatography (petroleum ether : ethyl acetate = 10 : 1, Rf = 0.4) to give the titled product **3bm** as a yellow oil (59.5 mg, 85%).

^{1}H NMR (400 MHz, CDCl$_3$) δ 7.88 (d, J = 8.2 Hz, 2H), 7.28 (d, J = 8.0 Hz, 2H), 7.11 (d, J = 7.8 Hz, 1H), 7.00 (d, J = 7.7 Hz, 1H), 6.93 (s, 1H), 3.88 (dd, J = 10.0, 7.5 Hz, 1H), 3.33 (t, 1H), 3.24 (dd, J = 16.8, 3.5 Hz, 1H), 3.01 (dd, J = 16.8, 10.6 Hz, 1H), 2.89 – 2.81 (m, 1H), 2.42 (s, 3H), 2.28 (s, 3H), 2.16 (s, 3H), 1.30 (s, 3H), 1.21 (s, 3H).

^{13}C NMR (101 MHz, CDCl$_3$) δ 198.1, 178.0, 144.3, 137.0, 136.4, 134.1, 132.1, 130.8, 129.4, 128.5, 128.0, 127.1, 53.1, 43.1, 40.1, 37.2, 23.8, 21.6, 20.7, 19.2, 17.4.

HRMS (ESI-TOF): m/z: $[M+H]^+$ Calcd. For $C_{23}H_{28}NO_2$ 350.2115; Found 350.2118.

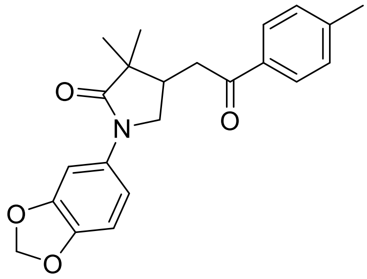

1-(benzo[*d*][1,3]dioxol-5-yl)-3,3-dimethyl-4-(2-oxo-2-(*p*-tolyl)ethyl)pyrrolidin-2-one (3bn)

Upon completion the mixture was concentrated and purified via flash column chromatography (petroleum ether : ethyl acetate = 10 : 1, Rf = 0.4) to give the titled product **3bn** as a yellow oil (54.8 mg, 75%).

^{1}H NMR (400 MHz, CDCl$_3$) δ 7.87 (d, J = 8.2 Hz, 2H), 7.37 (d, J = 2.1 Hz, 1H), 7.27 (d, J = 9.2 Hz, 2H), 6.86 (dd, J = 8.5, 2.2 Hz, 1H), 6.74 (d, J = 8.4 Hz, 1H), 5.91 (s, 2H), 4.02 (dd, J = 9.8, 7.6 Hz, 1H), 3.37 (t, J = 9.3 Hz, 1H), 3.23 (dd, J = 17.0, 3.4 Hz,

1H), 2.96 (dd, J = 17.0, 10.7 Hz, 1H), 2.77 – 2.69 (m, 1H), 2.41 (s, 3H), 1.27 (s, 3H), 1.13 (s, 3H).

^{13}C NMR (101 MHz, CDCl$_3$) δ 198.0, 177.9, 147.6, 144.3, 144.2, 134.0, 133.9, 129.4, 128.0, 112.6, 107.7, 102.5, 101.1, 51.6, 44.1, 38.6, 37.1, 23.8, 21.6, 19.2.

HRMS (ESI-TOF): m/z: [M+H]$^+$ Calcd. For $C_{22}H_{24}NO_4$ 366.1700; Found 366.1707.

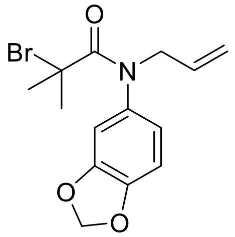

***N*-allyl-*N*-(benzo[*d*][1,3]dioxol-5-yl)-2-bromo-2-methylpropanamide**

Upon completion the mixture was concentrated and purified via flash column chromatography (petroleum ether : ethyl acetate = 20 : 1, Rf = 0.3) to give the titled product as a yellow oil .

^{1}H NMR (400 MHz, CDCl$_3$) δ 6.77 (dd, J = 14.3, 7.2 Hz, 3H), 5.99 (s, 2H), 5.92 – 5.82 (m, 1H), 5.09 (dd, J = 22.9, 13.6 Hz, 2H), 4.21 (s, 2H), 1.73 (s, 6H).

^{13}C NMR (101 MHz, CDCl$_3$) δ 169.8, 147.6, 147.2, 136.0, 132.3, 123.1, 118.2, 110.3, 107.7, 101.7, 58.1, 56.4, 33.3.

HRMS (ESI-TOF): m/z: [M+H]$^+$ Calcd. For $C_{14}H_{16}BrNO_3$ 326.0386; Found 326.0392.

4. Reference

(1) Li, M.; L, Y.; Jia, W. -Y.; Sun, G. -Q.; Gao, F.; Zhao, G. -X.; Qiu, Y. -F.; Wang, X. -C.; Liang, Y. -M.; Quan, Z. -J. Directed Copper-Catalyzed Tandem Radical Cyclization Reaction of Alkyl Bromides and Unactivated Olefins. *Org. Lett.* **2022**, *24*, 2738-2743.

5. Copy of ^{1}H and ^{13}C NMR Spectra of Products

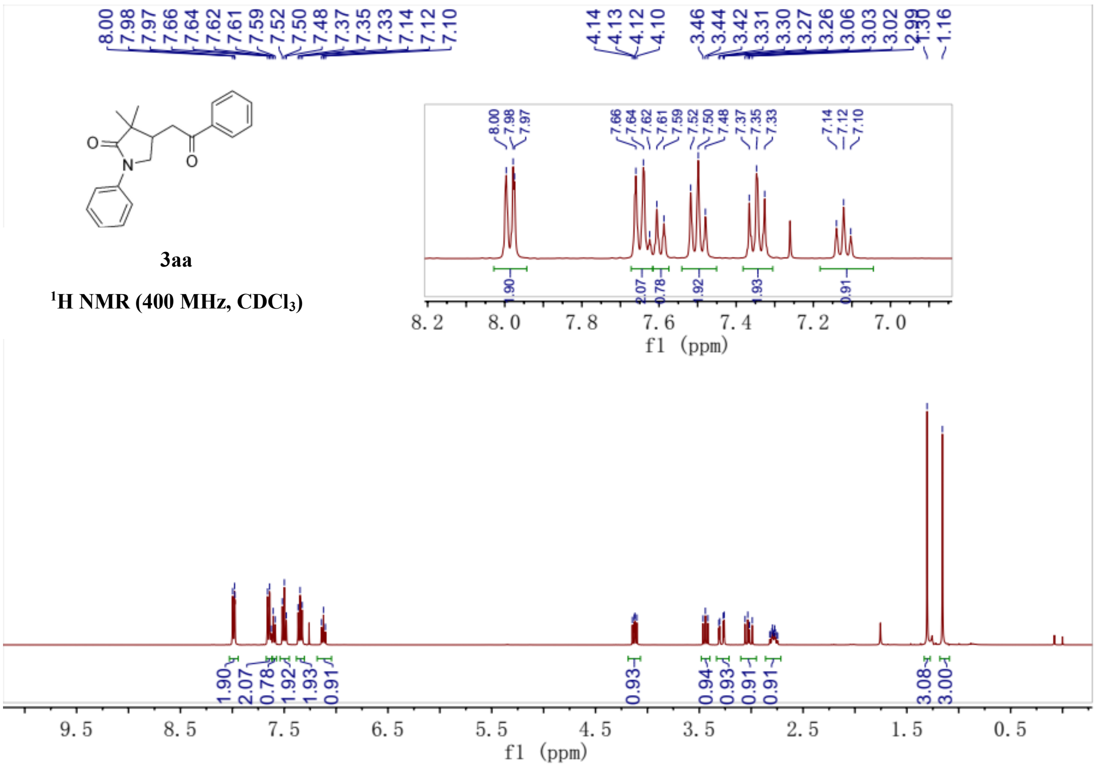

3aa

^{1}H NMR (400 MHz, $CDCl_3$)

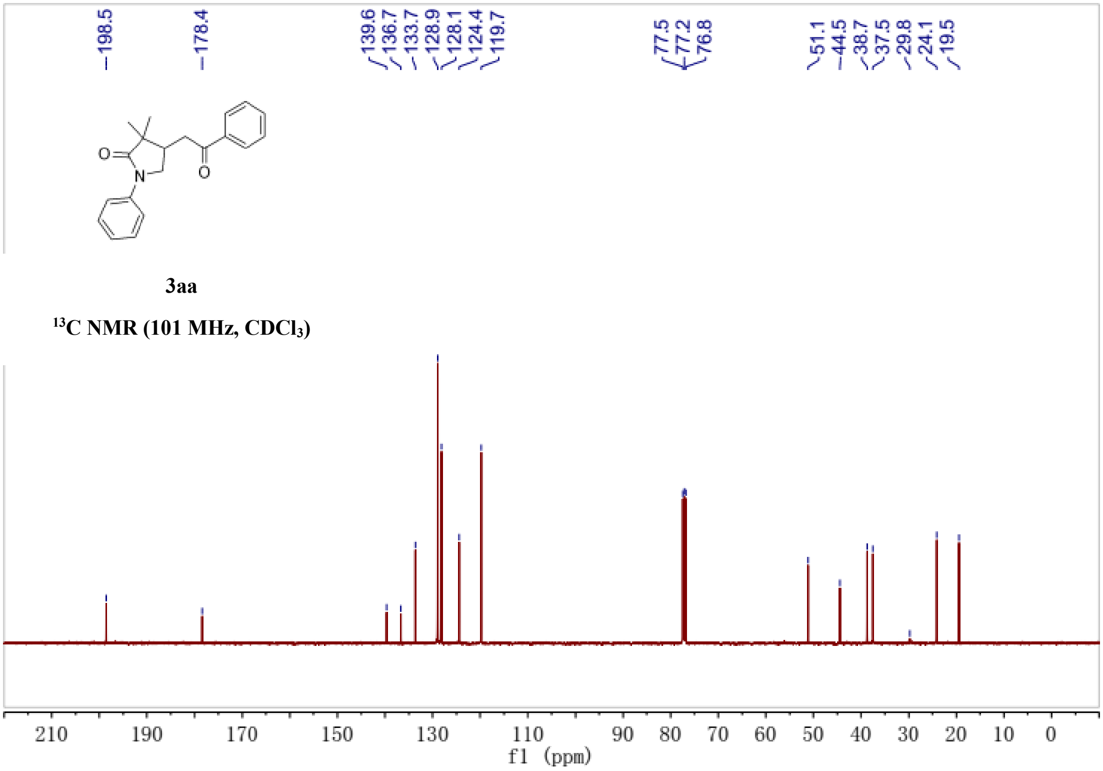

3aa

^{13}C NMR (101 MHz, $CDCl_3$)

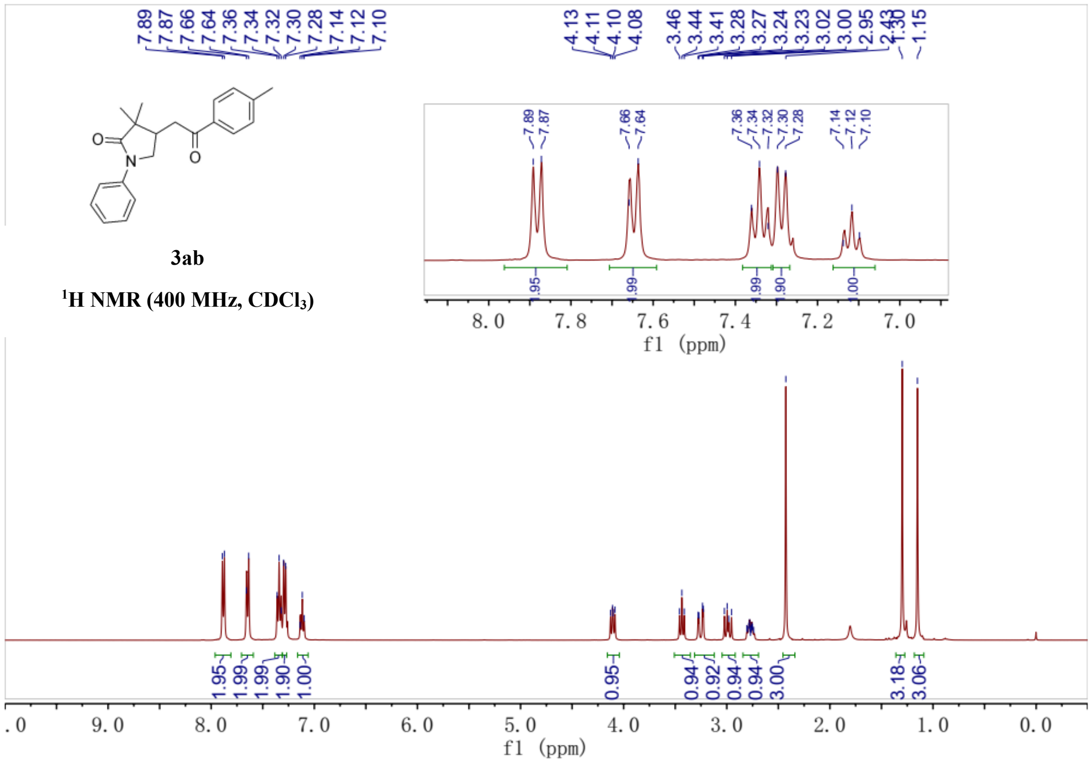

3ab

^{1}H NMR (400 MHz, $CDCl_3$)

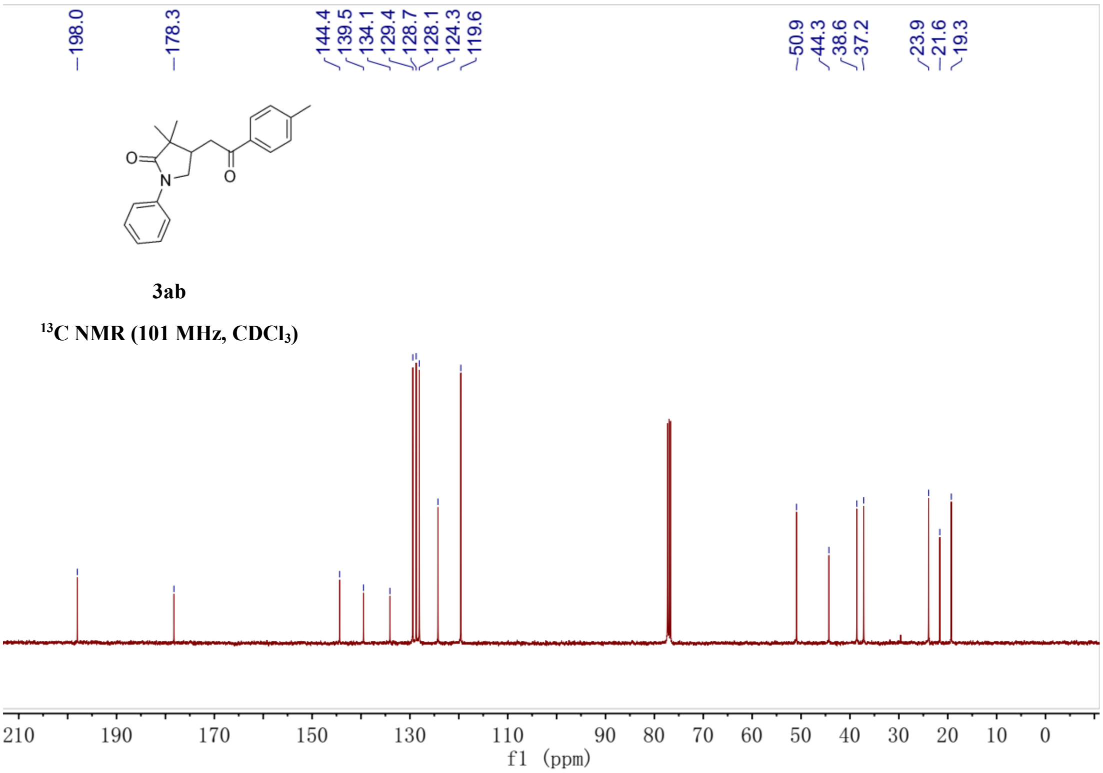

3ab

^{13}C NMR (101 MHz, $CDCl_3$)

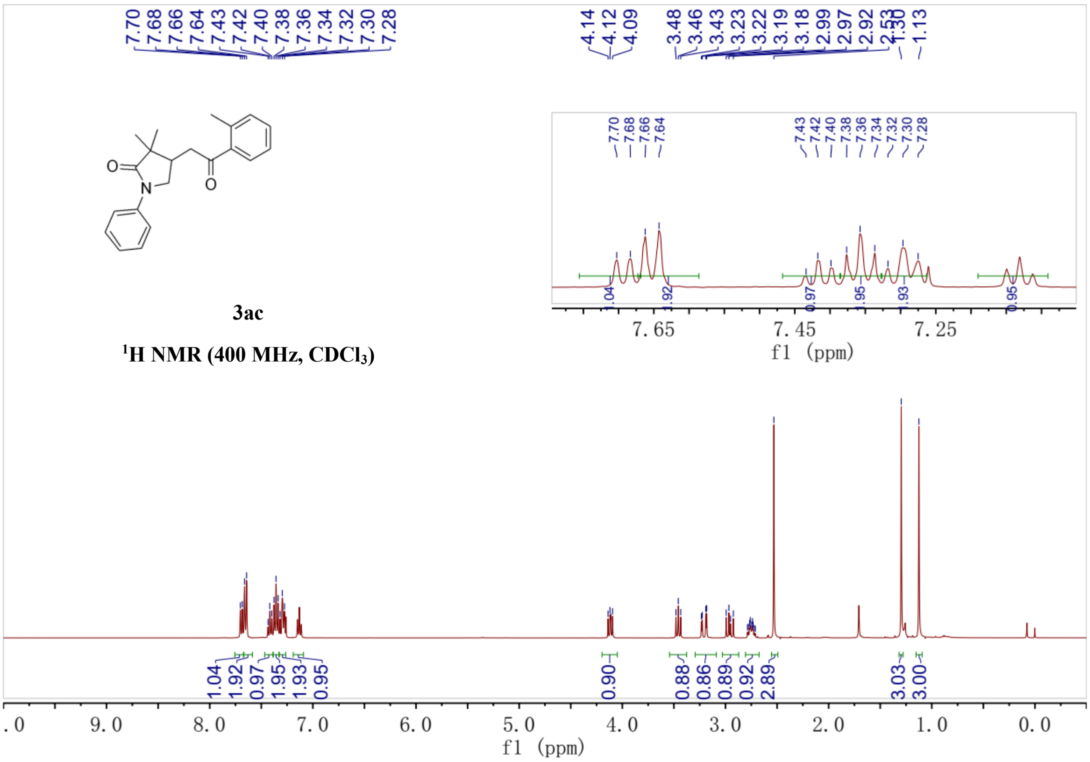
7.70 7.68 7.66 7.64 7.43 7.42 7.40 7.38 7.36 7.34 7.32 7.30 7.28
4.14 4.12 4.09 3.48 3.46 3.43 3.23 3.22 3.19 3.18 2.99 2.97 2.92 2.53 1.30 1.13
3ac
1H NMR (400 MHz, CDCl3)
1.04 1.92 0.97 1.95 1.93 0.95 0.90 0.88 0.86 0.89 0.92 2.89 3.03 3.00
f1 (ppm)

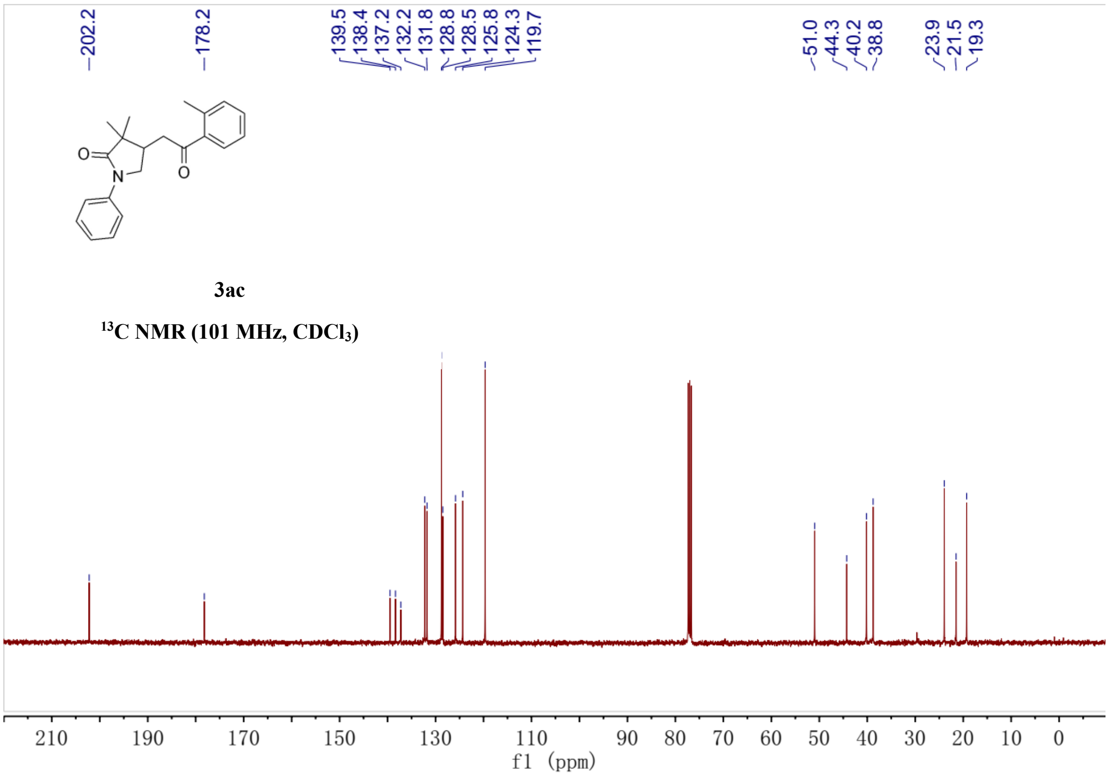
202.2 178.2 139.5 138.4 137.2 132.2 131.8 128.8 128.5 125.8 124.3 119.7 51.0 44.3 40.2 38.8 23.9 21.5 19.3
3ac
13C NMR (101 MHz, CDCl3)
f1 (ppm)

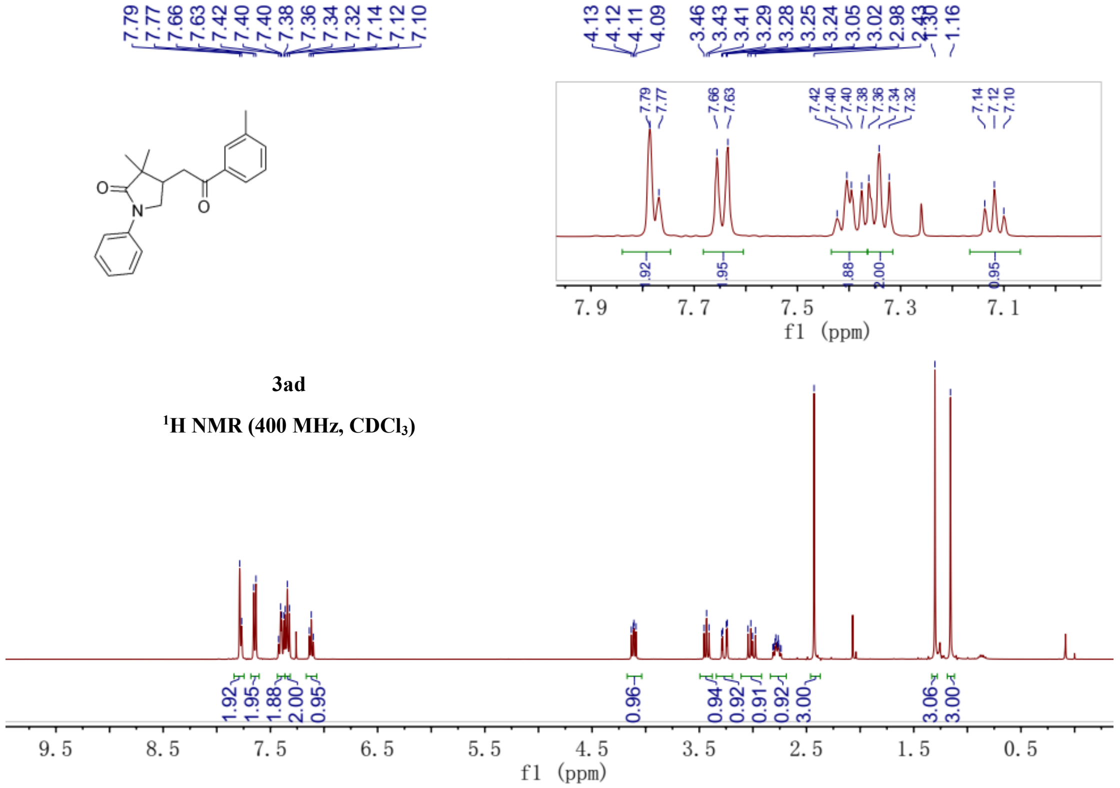

3ad

1H NMR (400 MHz, CDCl$_3$)

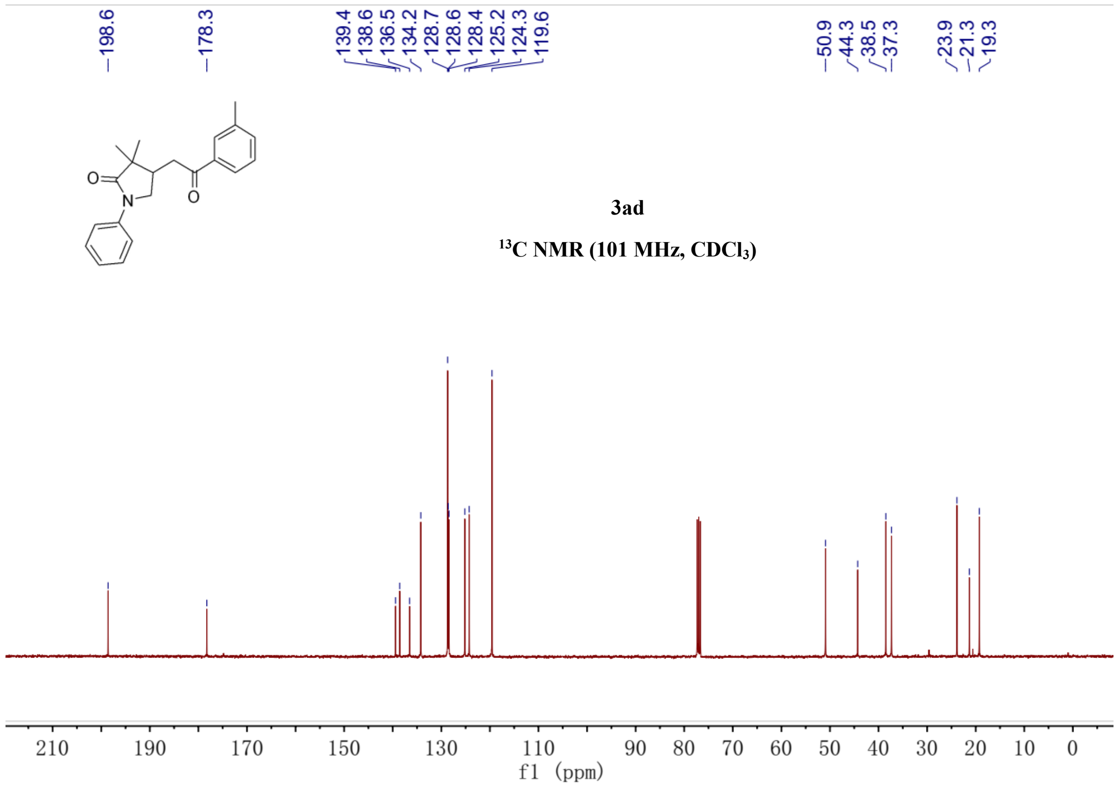

3ad

13C NMR (101 MHz, CDCl$_3$)

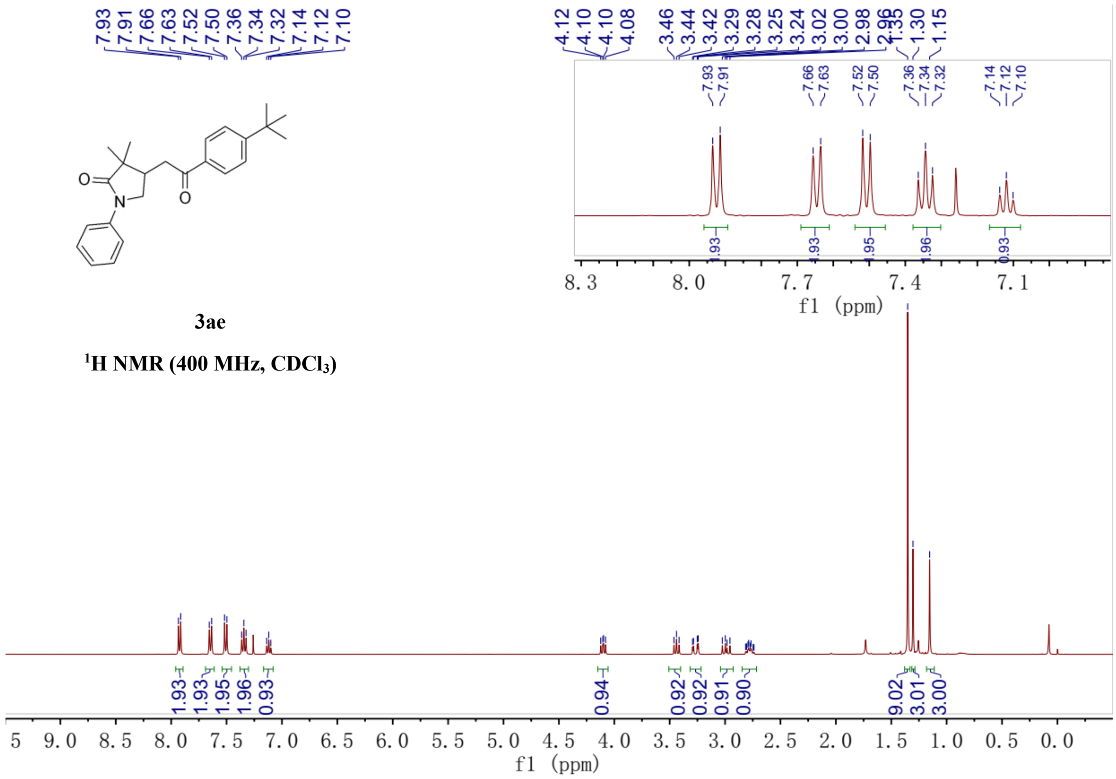

3ae

^{1}H NMR (400 MHz, CDCl$_3$)

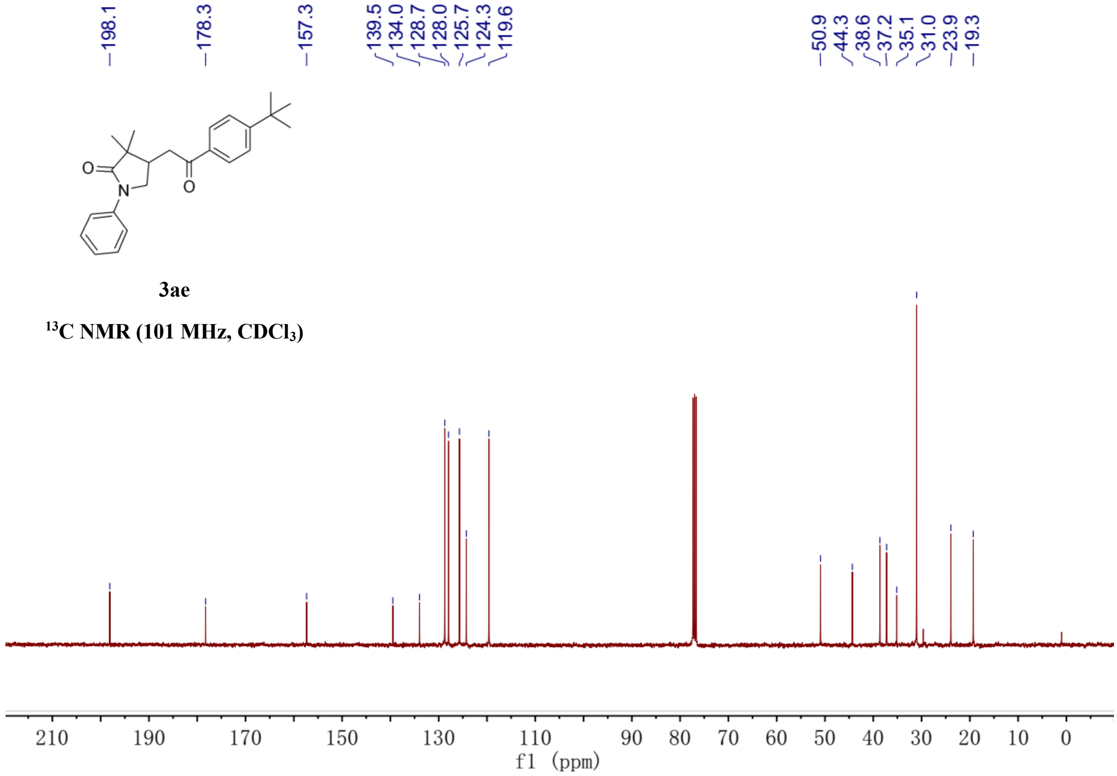

3ae

^{13}C NMR (101 MHz, CDCl$_3$)

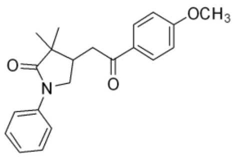

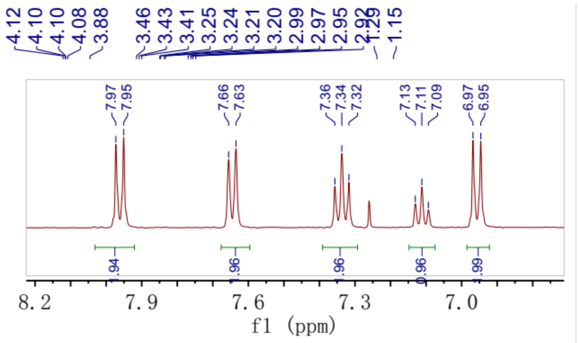

3af

^{1}H NMR (400 MHz, CDCl$_3$)

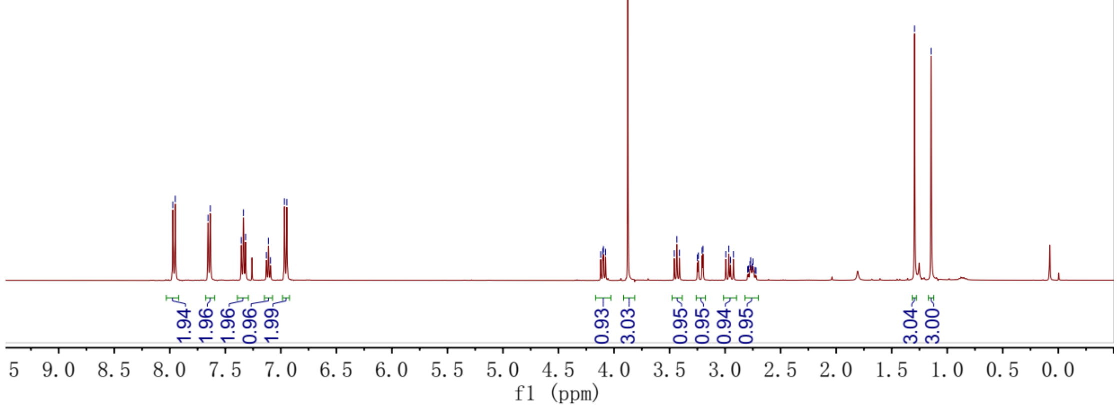

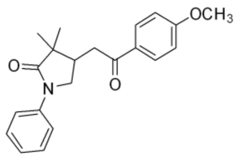

3af

^{13}C NMR (101 MHz, CDCl$_3$)

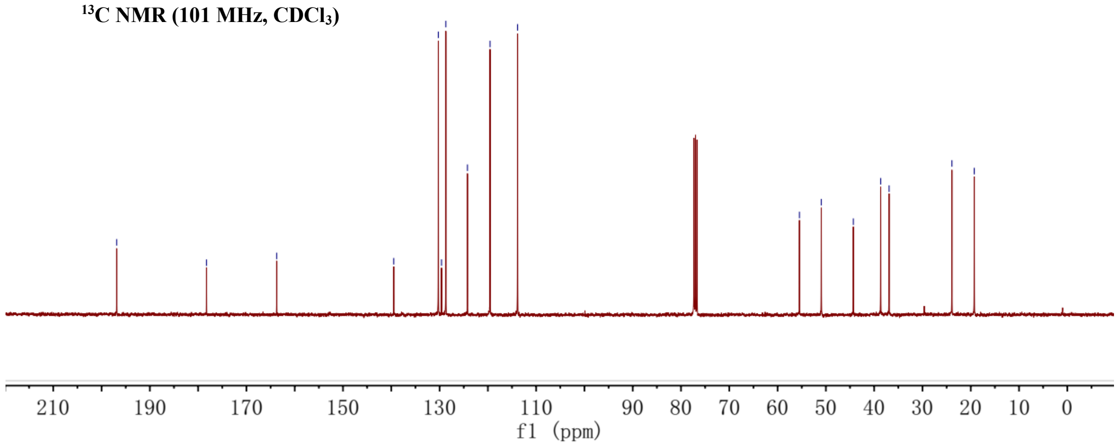

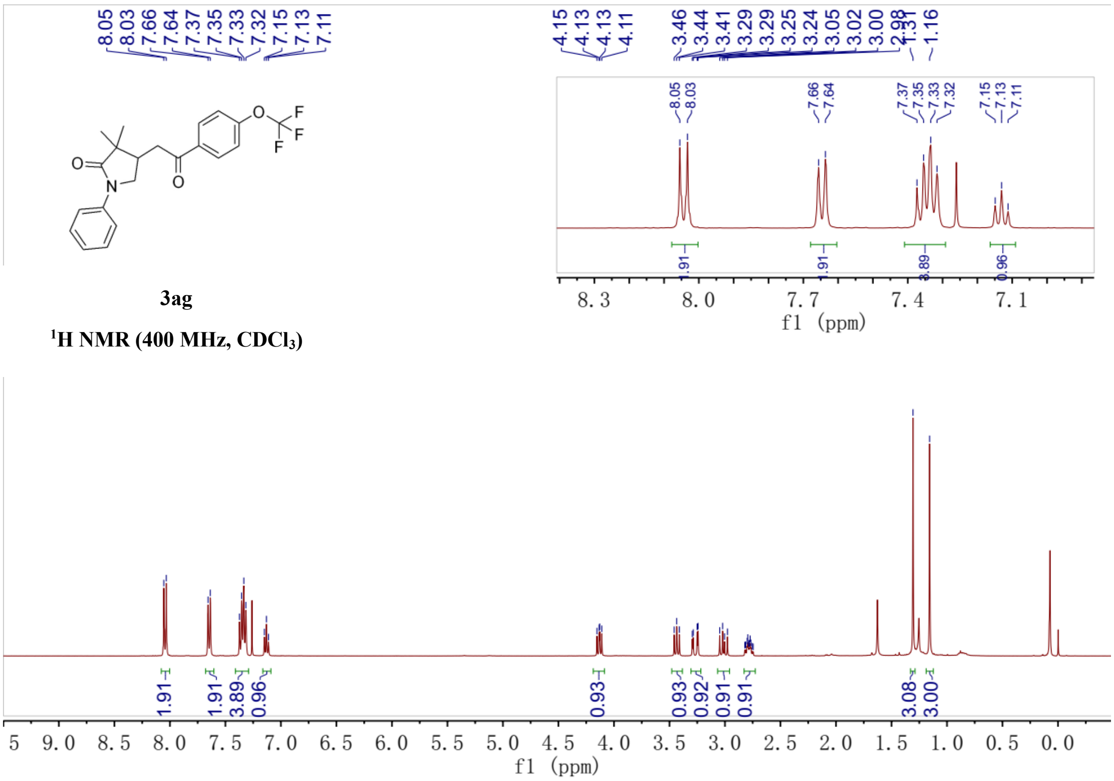

3ag

^{1}H NMR (400 MHz, CDCl$_3$)

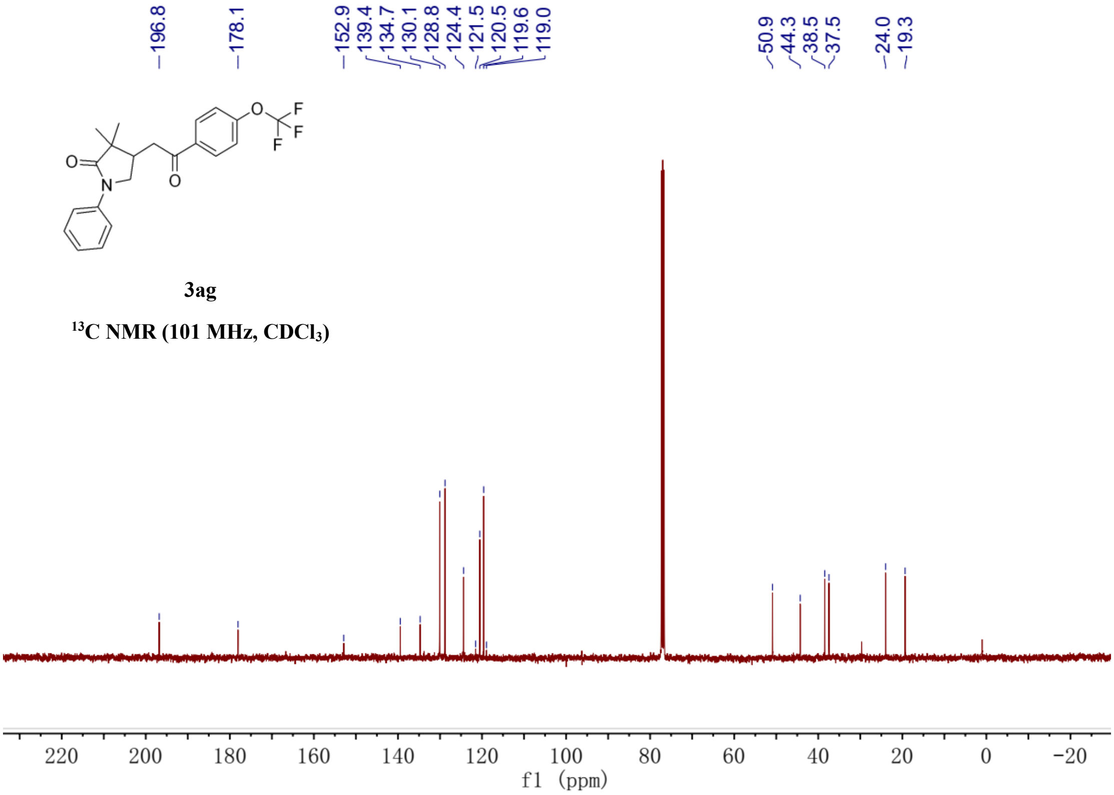

3ag

^{13}C NMR (101 MHz, CDCl$_3$)

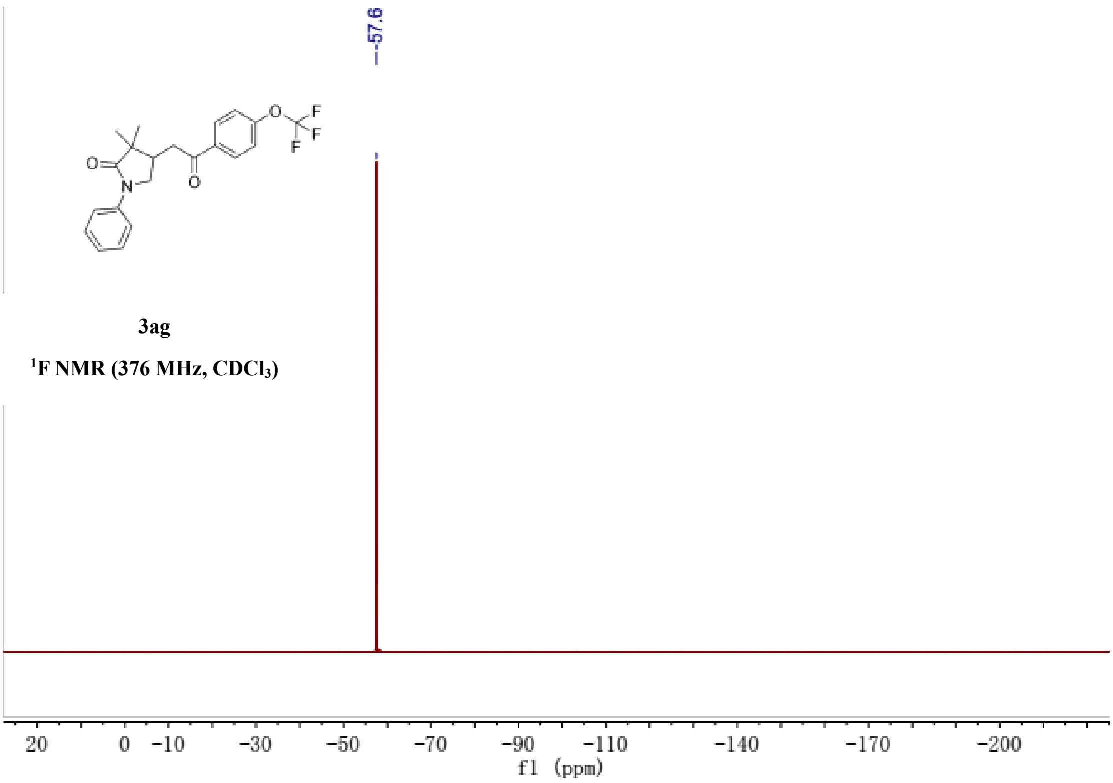
-57.6
3ag
1F NMR (376 MHz, CDCl3)
20
0
-10
-30
-50
-70
-90
-110
-140
-170
-200
f1 (ppm)

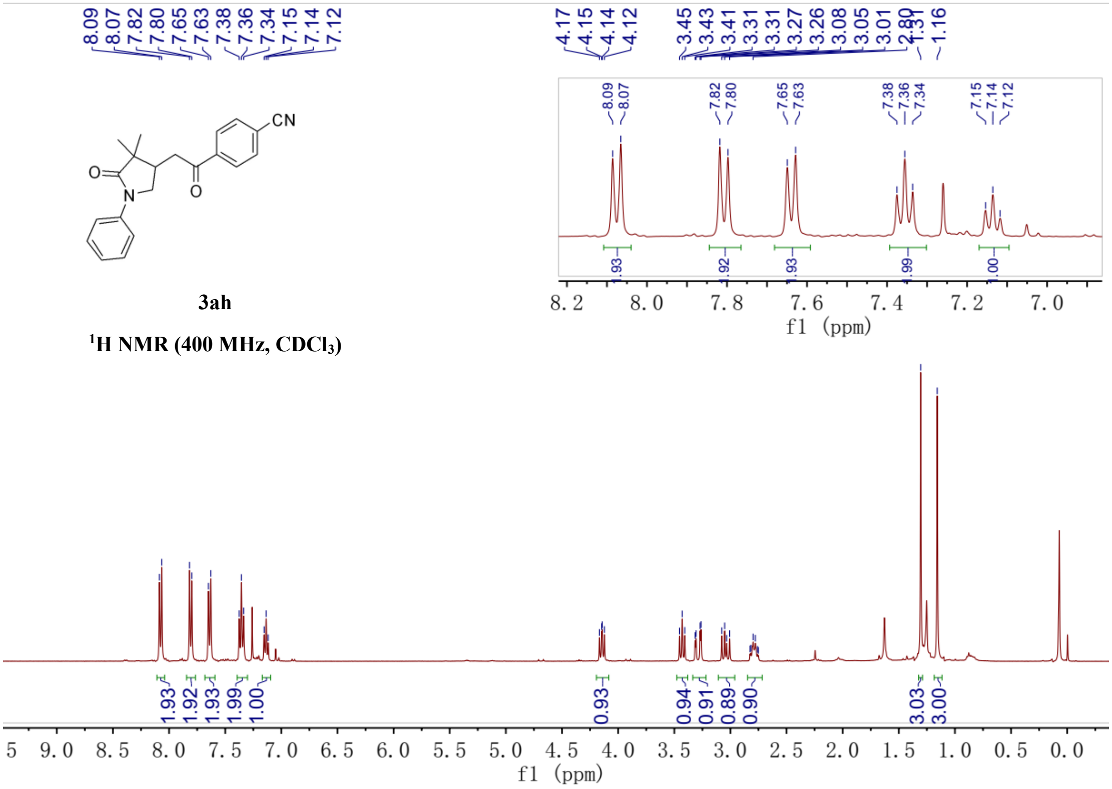

3ah

^{1}H NMR (400 MHz, $CDCl_3$)

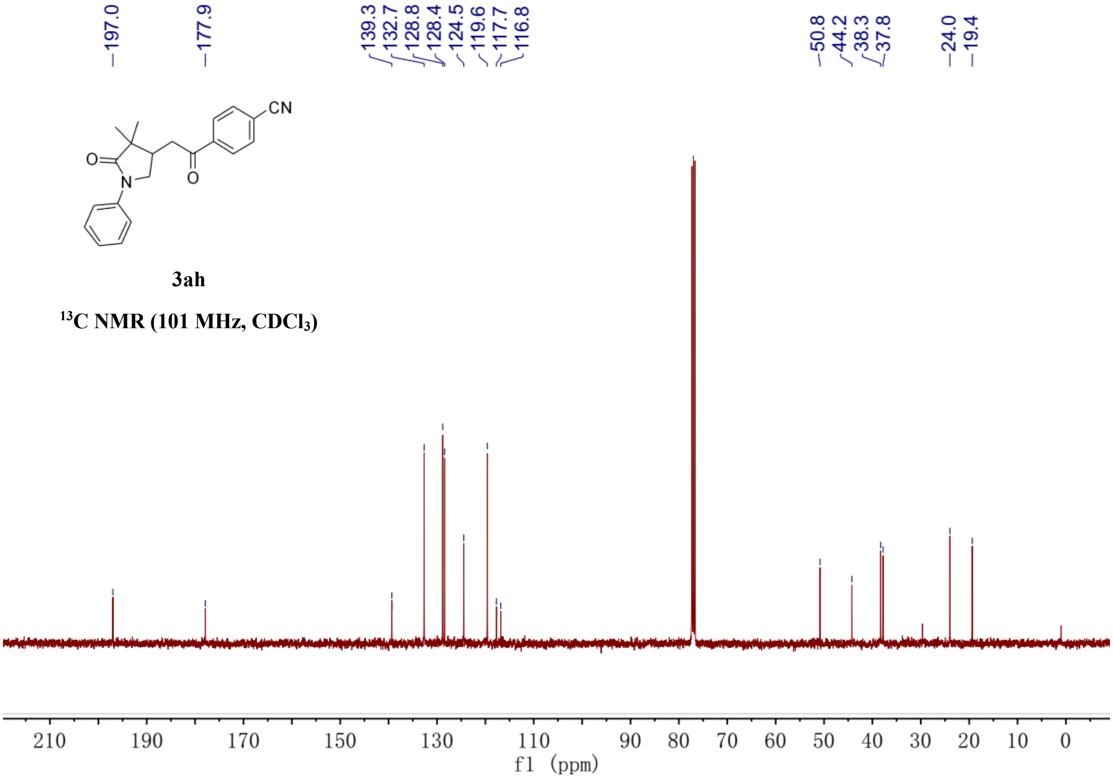

3ah

^{13}C NMR (101 MHz, $CDCl_3$)

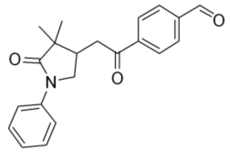

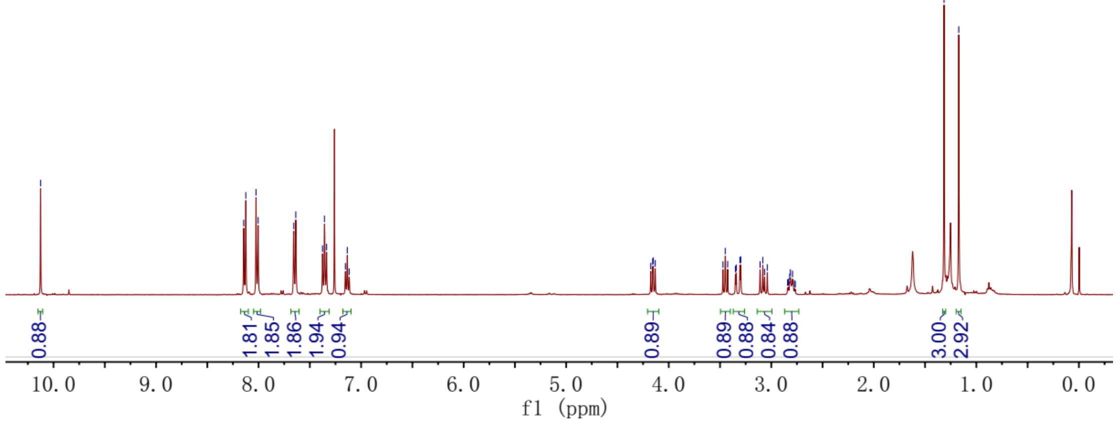

3ai

^{1}H NMR (400 MHz, CDCl$_3$)

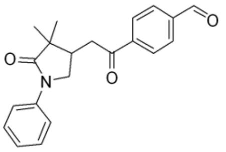

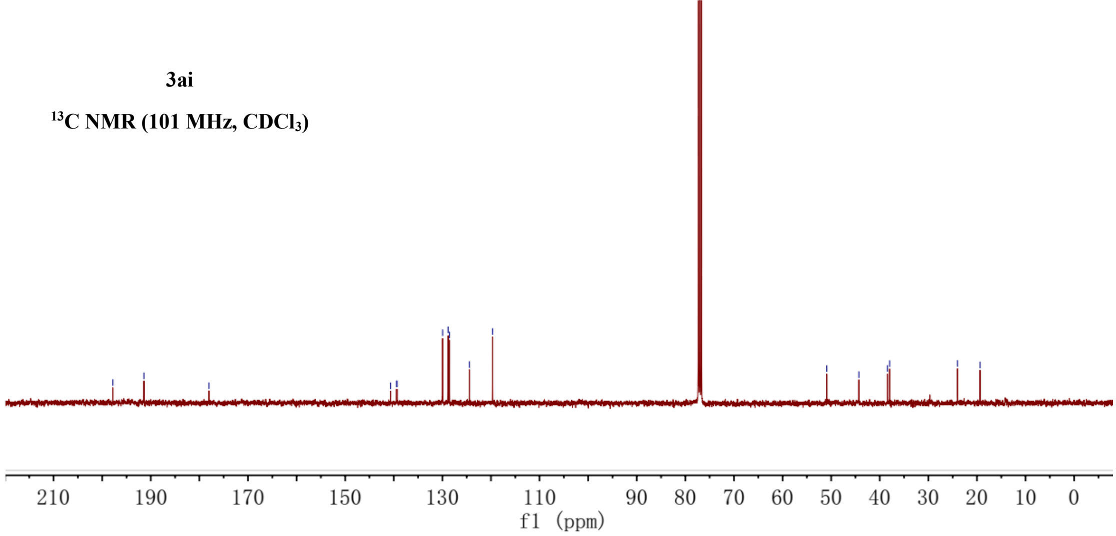

3ai

^{13}C NMR (101 MHz, CDCl$_3$)

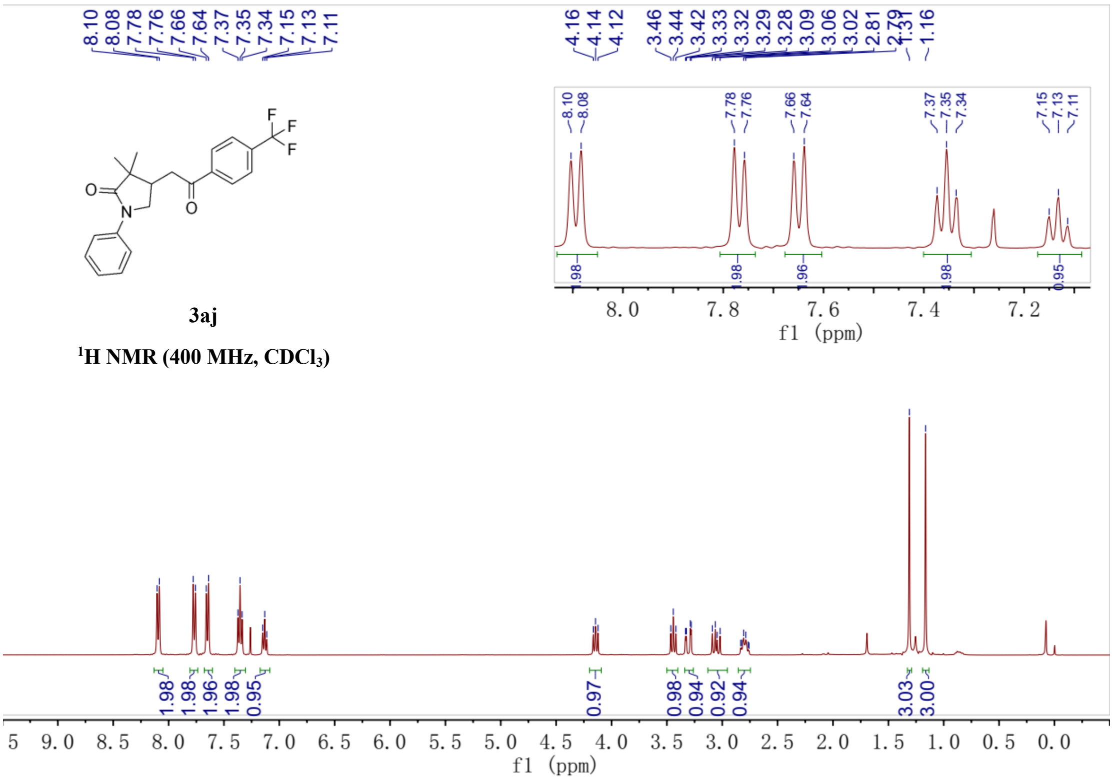

3aj

^{1}H NMR (400 MHz, CDCl$_3$)

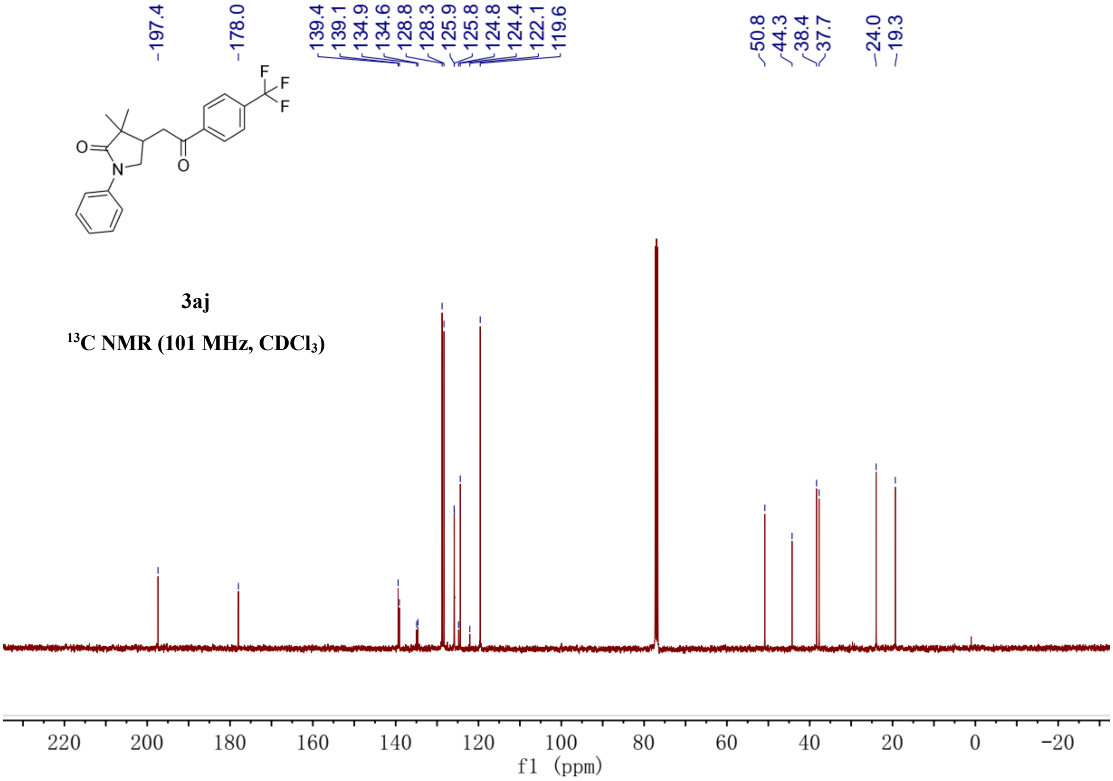

3aj

^{13}C NMR (101 MHz, CDCl$_3$)

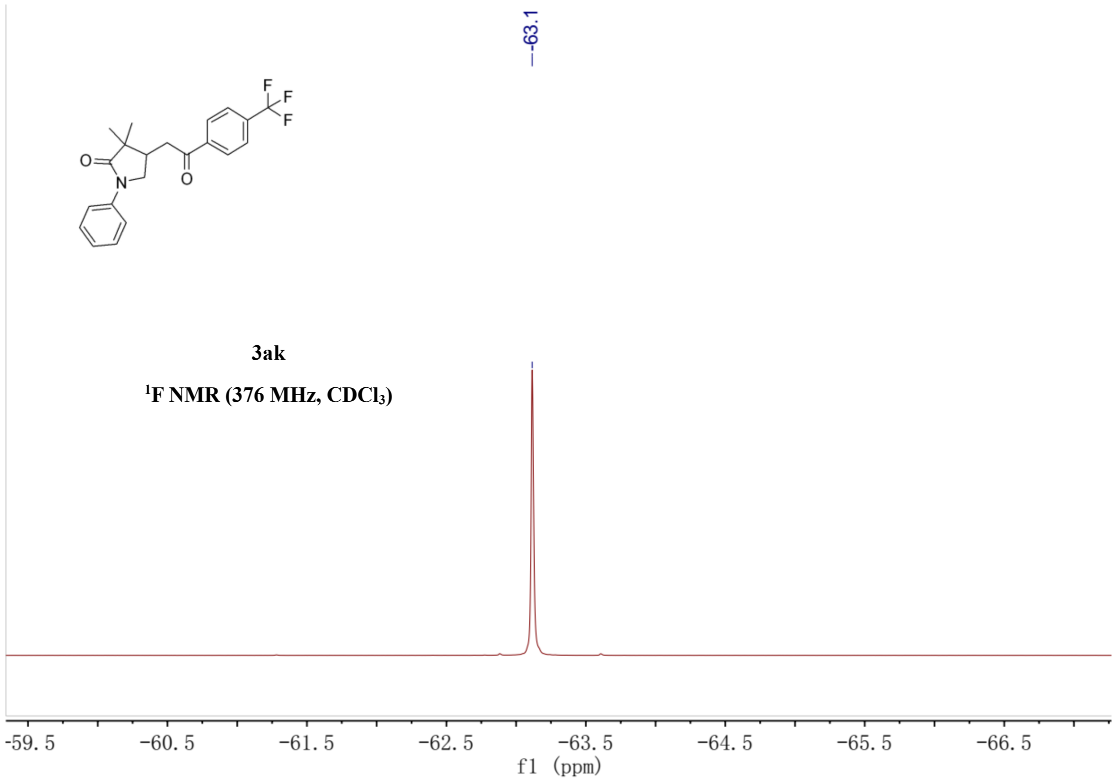
-63.1
3ak
^{1}F NMR (376 MHz, $CDCl_3$)
-59.5
-60.5
-61.5
-62.5
-63.5
-64.5
-65.5
-66.5
f1 (ppm)

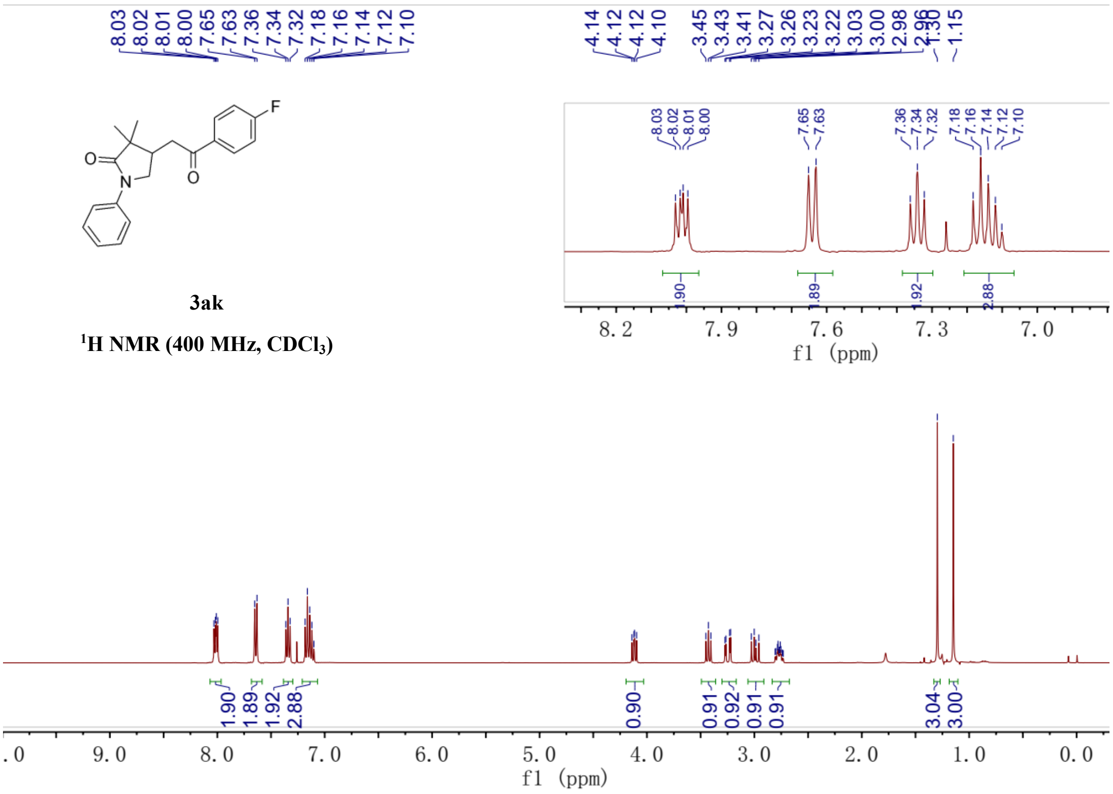

3ak

^{1}H NMR (400 MHz, CDCl$_3$)

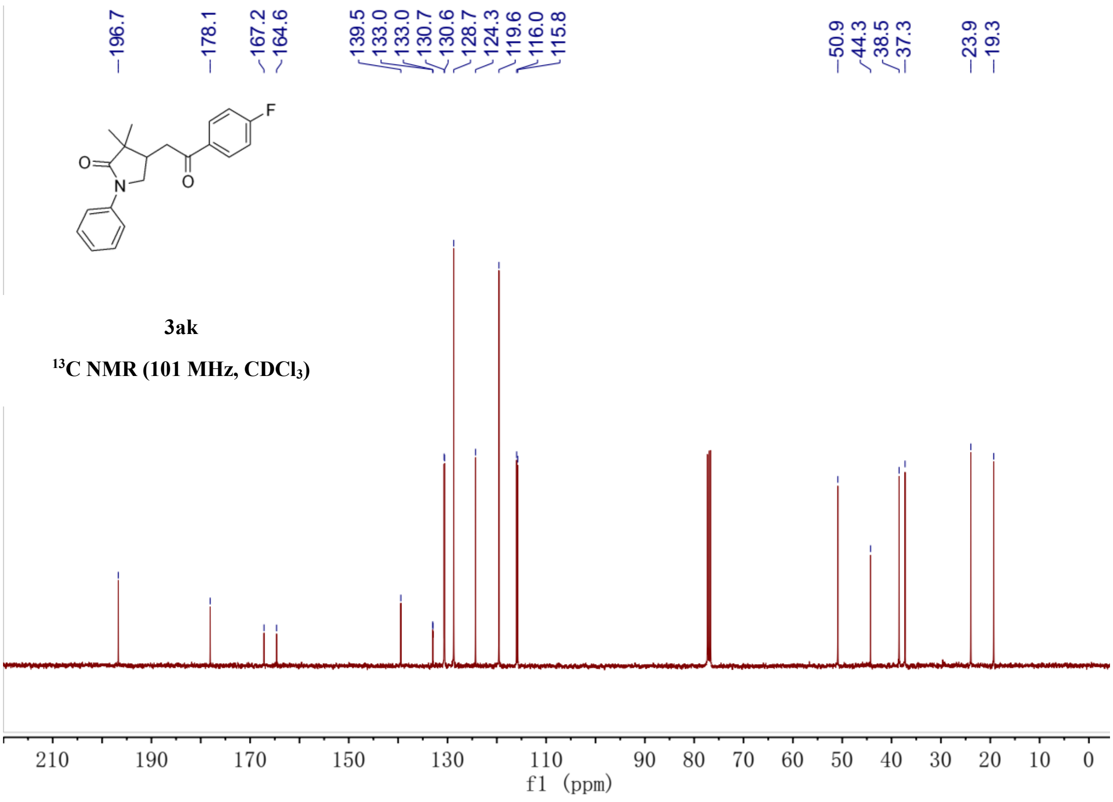

3ak

^{13}C NMR (101 MHz, CDCl$_3$)

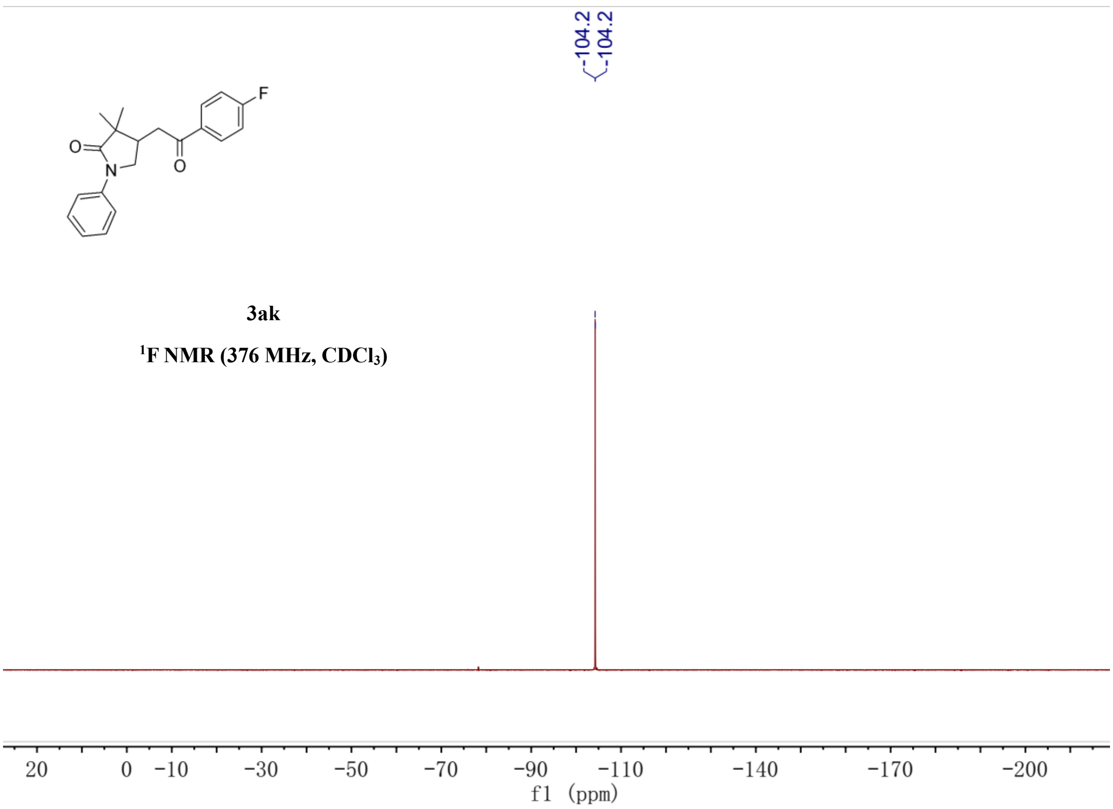

3ak

[1]F NMR (376 MHz, $CDCl_3$)

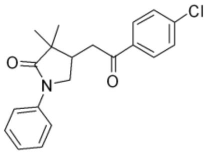

3al

^{1}H NMR (400 MHz, CDCl$_3$)

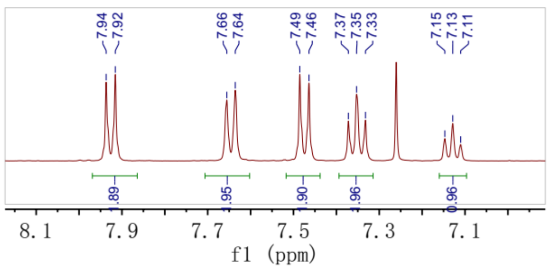

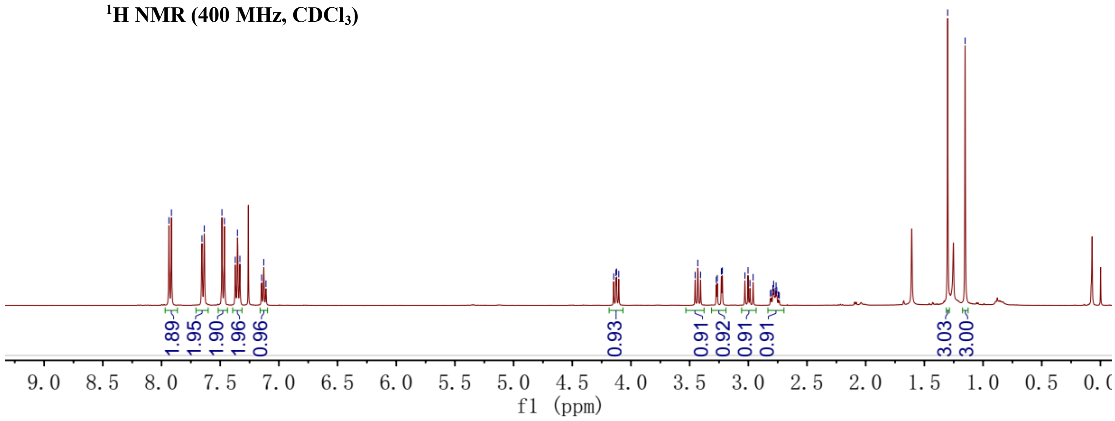

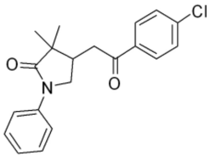

3al

^{13}C NMR (101 MHz, CDCl$_3$)

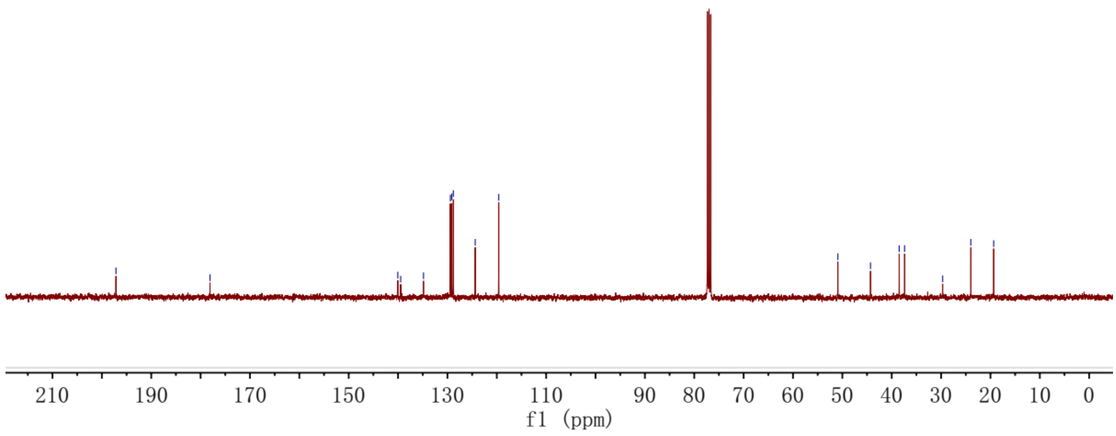

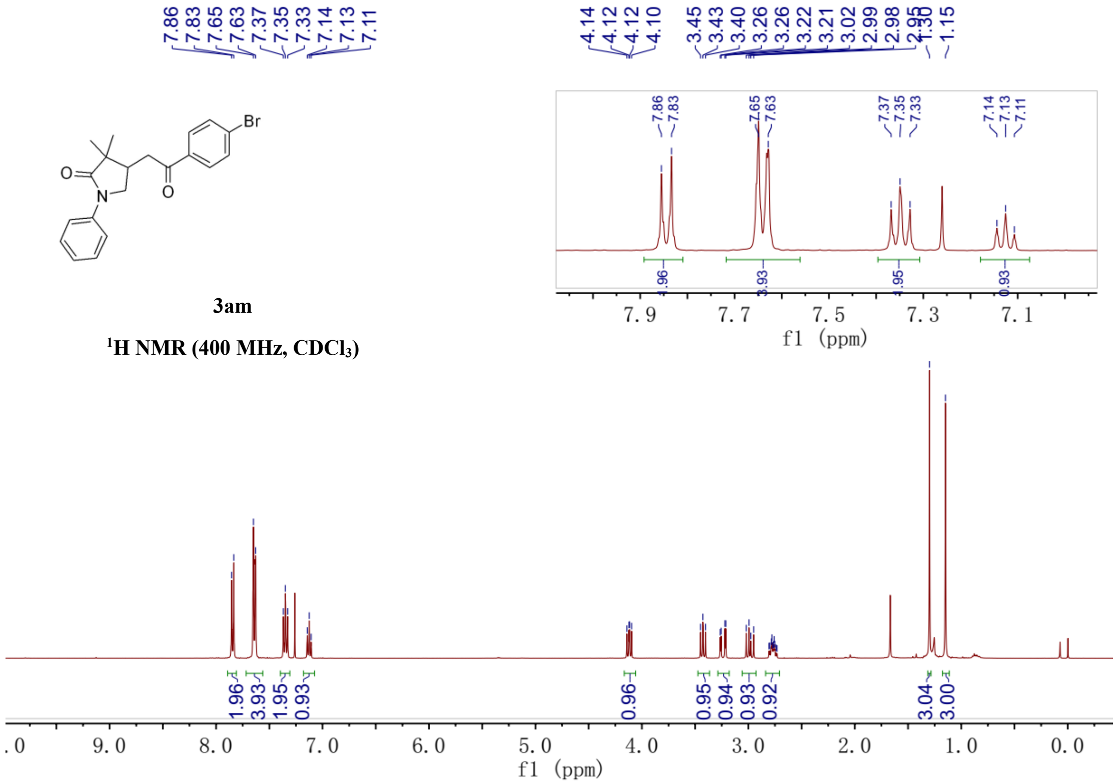

3am

^{1}H NMR (400 MHz, $CDCl_3$)

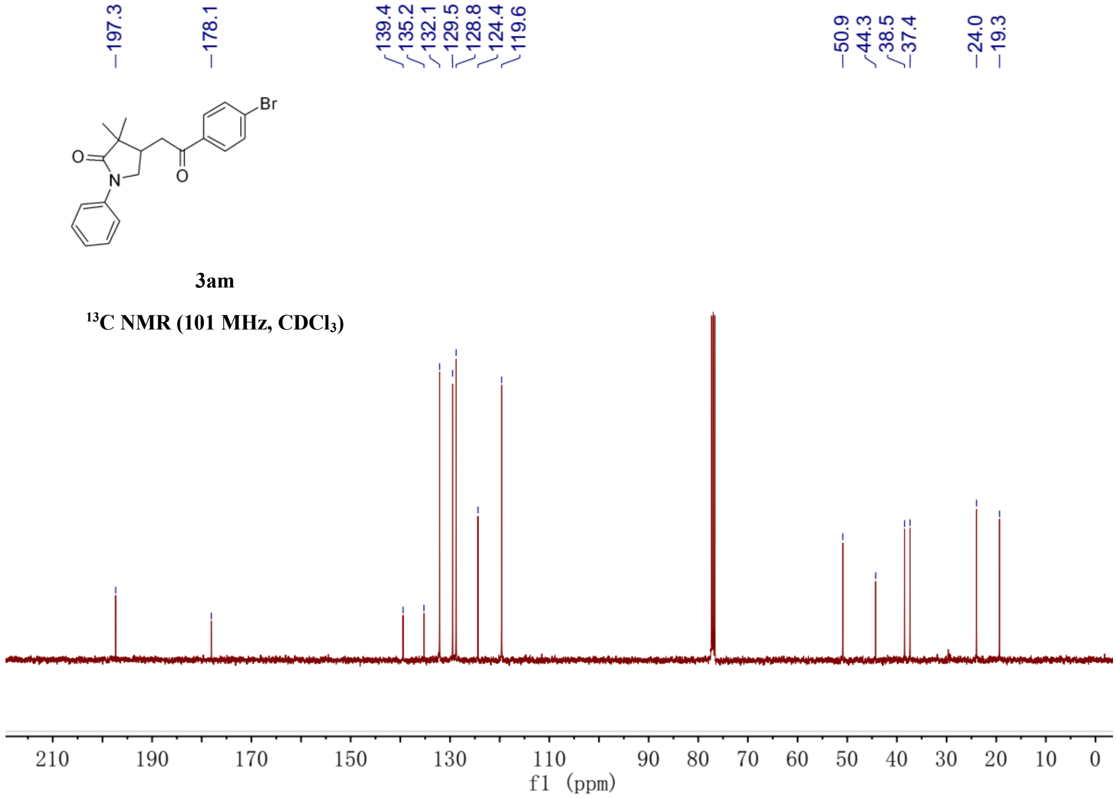

3am

^{13}C NMR (101 MHz, $CDCl_3$)

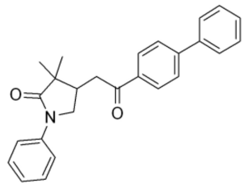

3an

^{1}H NMR (400 MHz, CDCl$_3$)

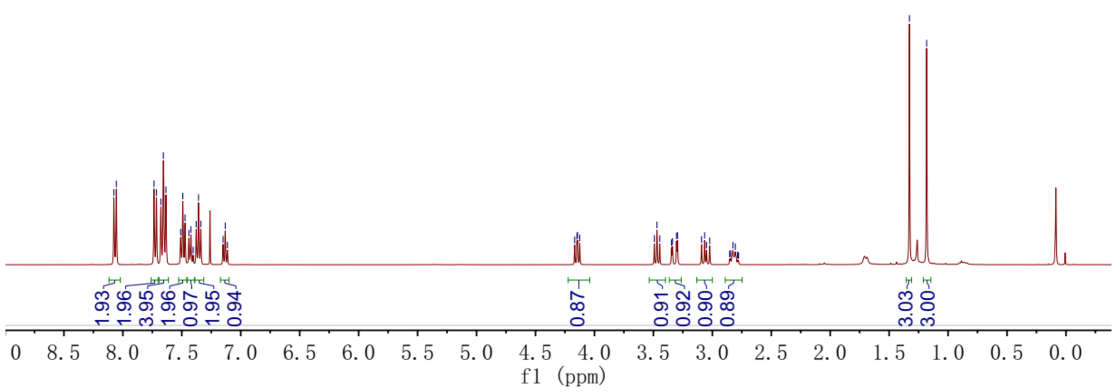

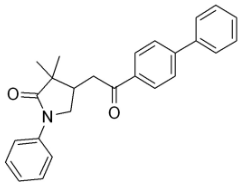

3an

^{13}C NMR (101 MHz, CDCl$_3$)

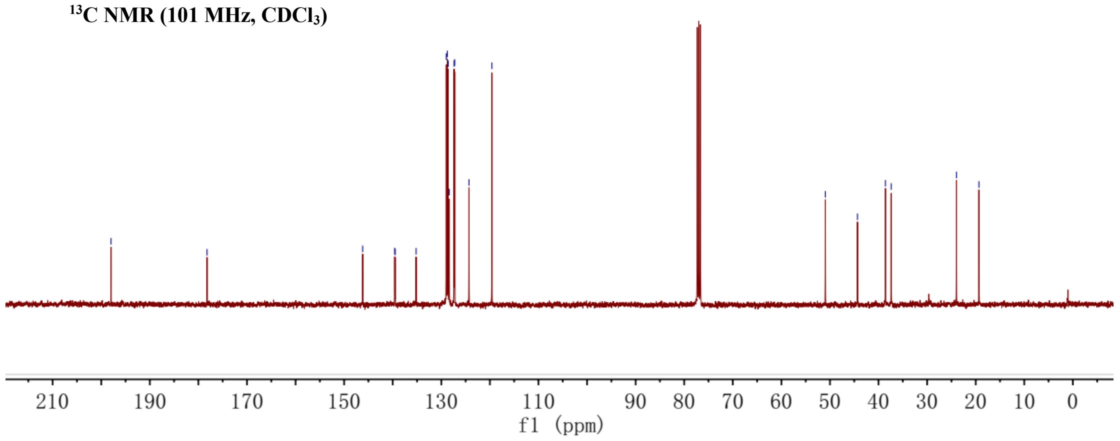

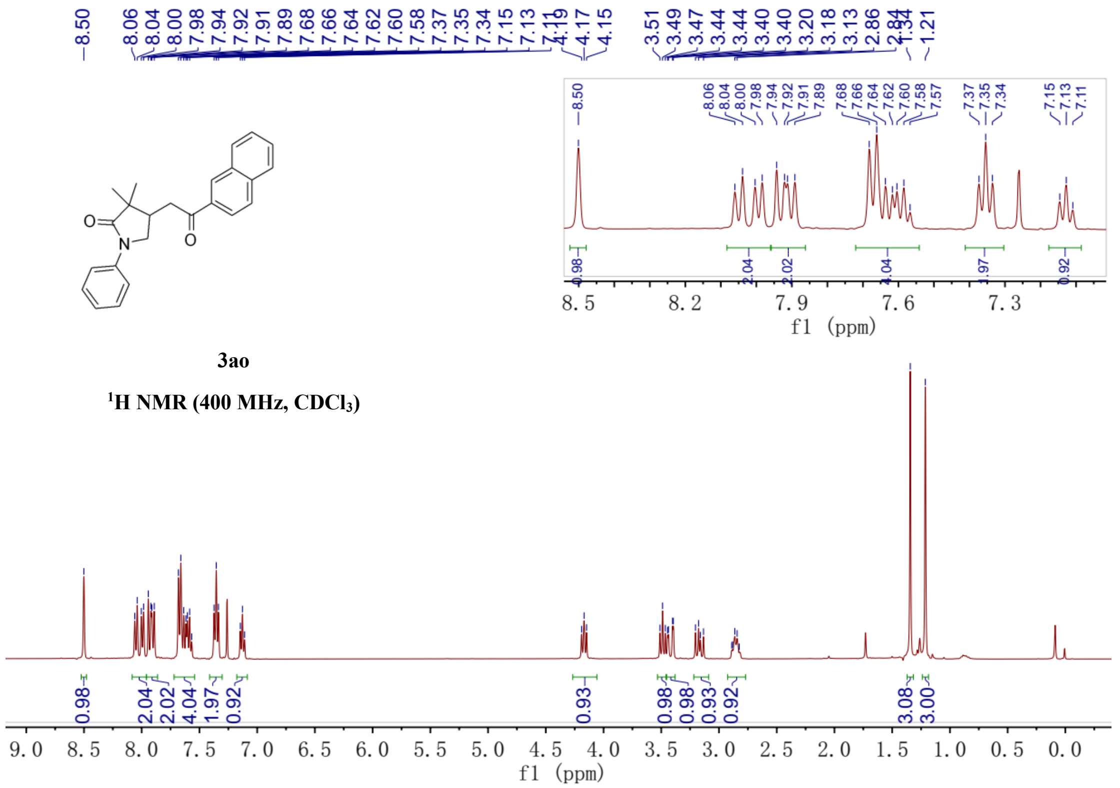

3ao

^{1}H NMR (400 MHz, $CDCl_3$)

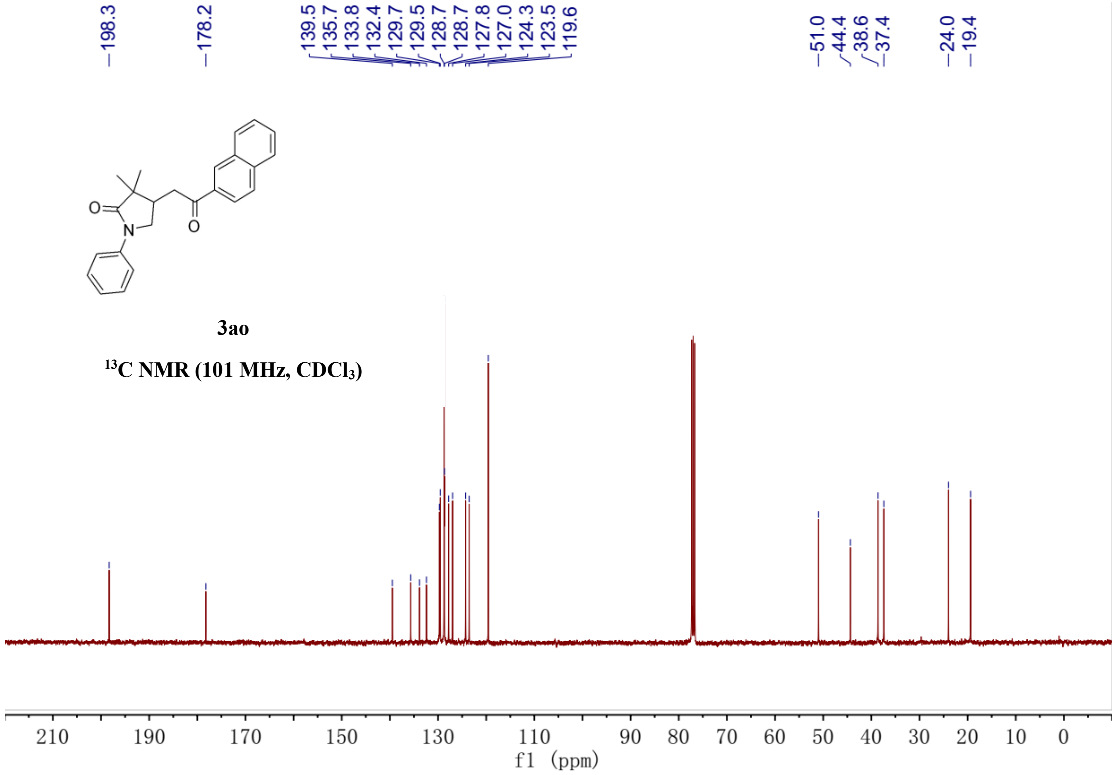

3ao

^{13}C NMR (101 MHz, $CDCl_3$)

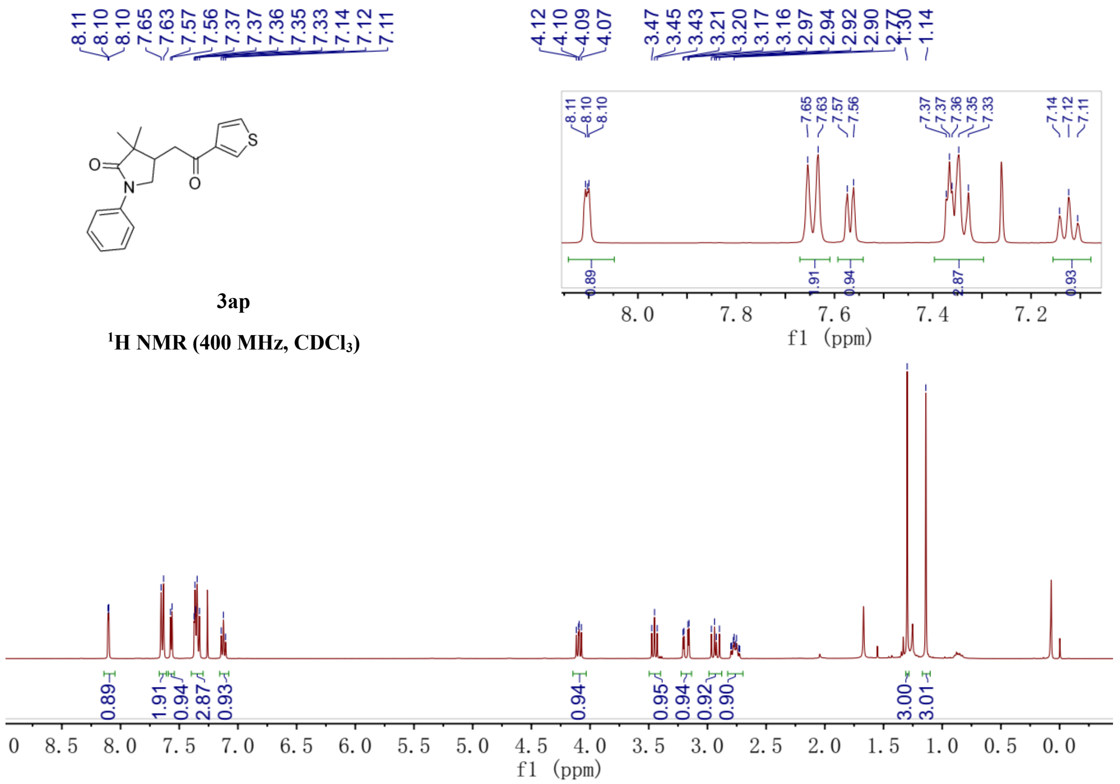

3ap

^{1}H NMR (400 MHz, CDCl$_3$)

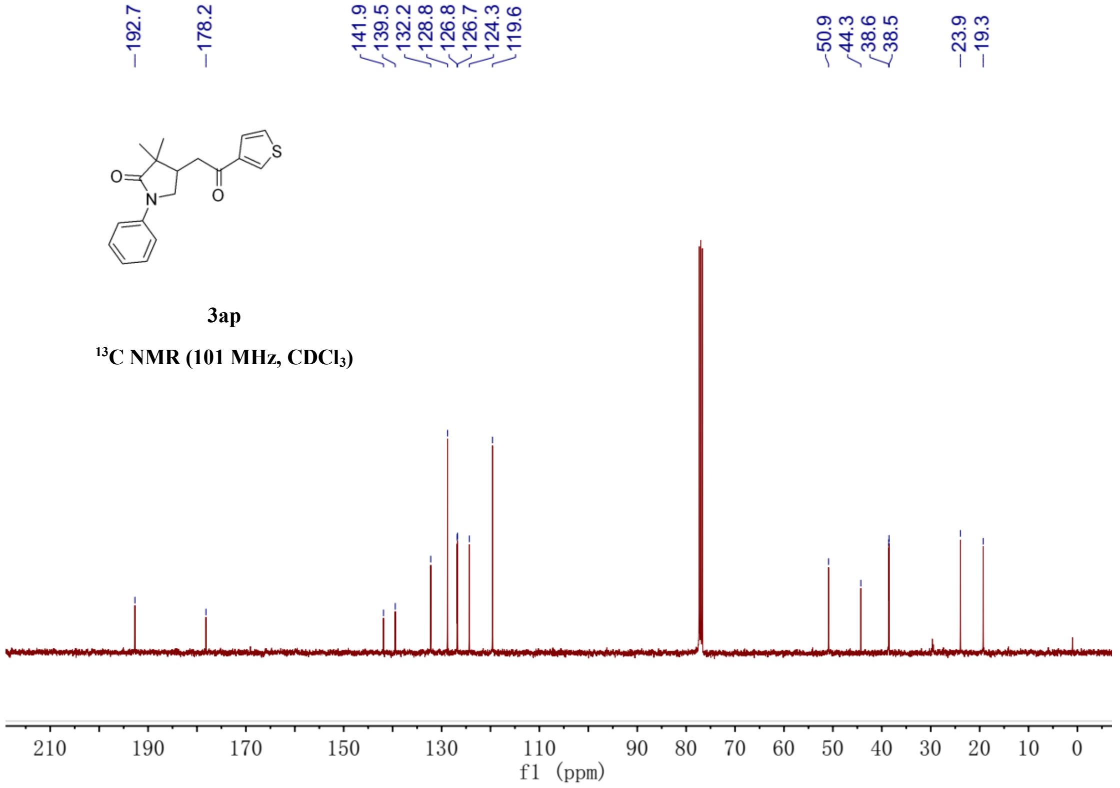

3ap

^{13}C NMR (101 MHz, CDCl$_3$)

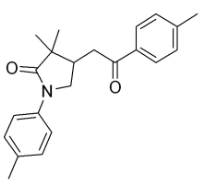

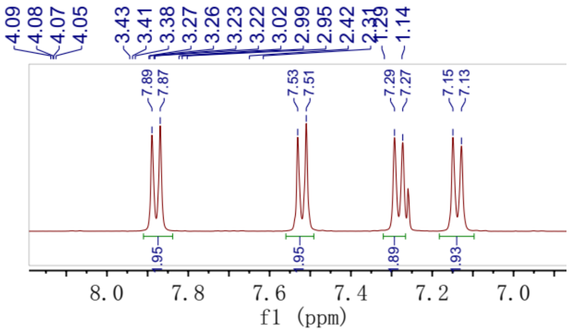

3bb

^{1}H NMR (400 MHz, CDCl$_3$)

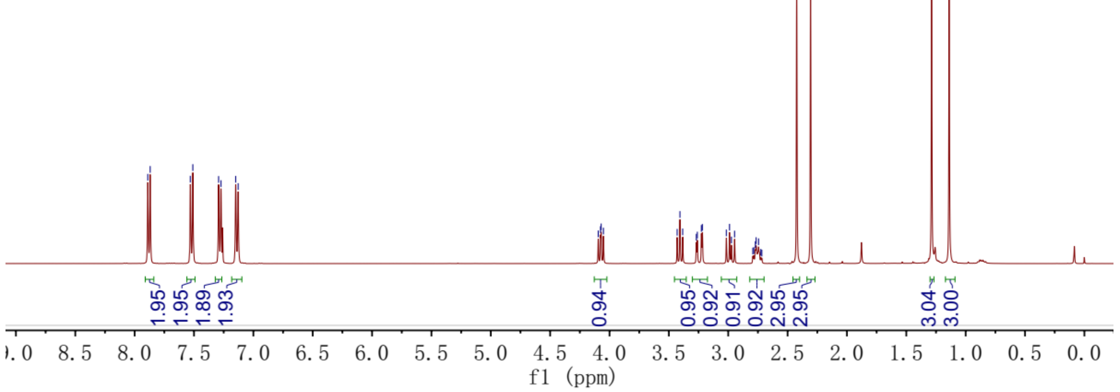

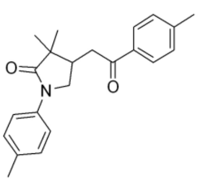

3bb

^{13}C NMR (101 MHz, CDCl$_3$)

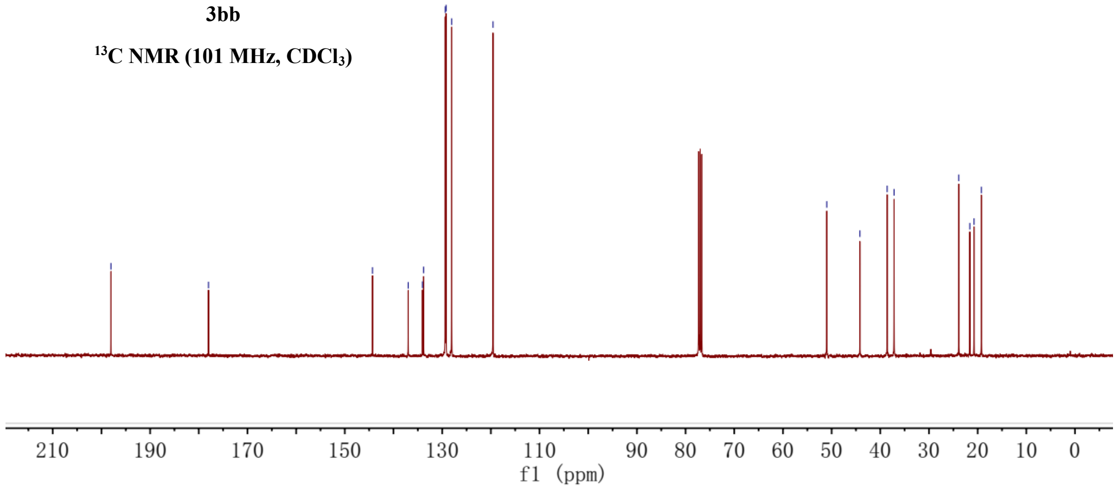

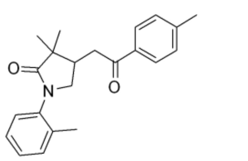

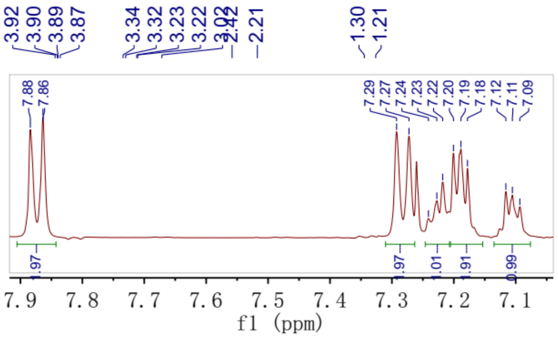

3bc

^{1}H NMR (400 MHz, CDCl$_3$)

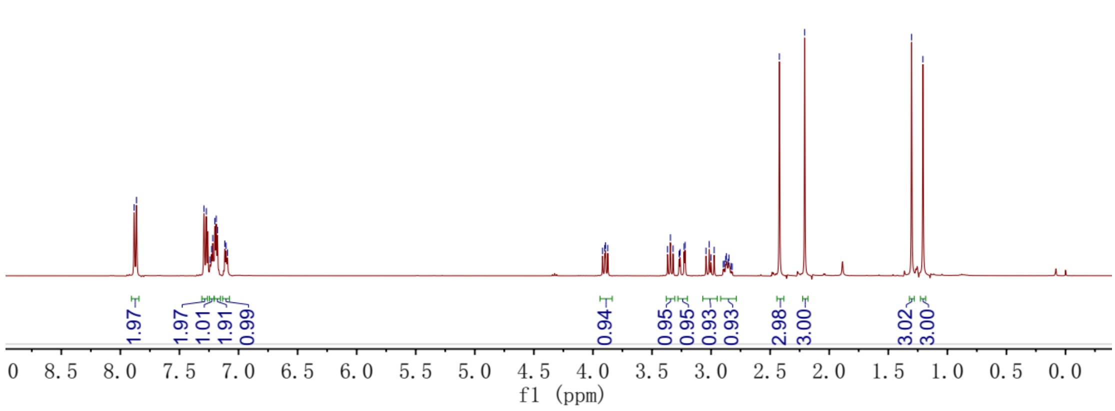

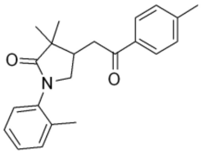

3bc

^{13}C NMR (101 MHz, CDCl$_3$)

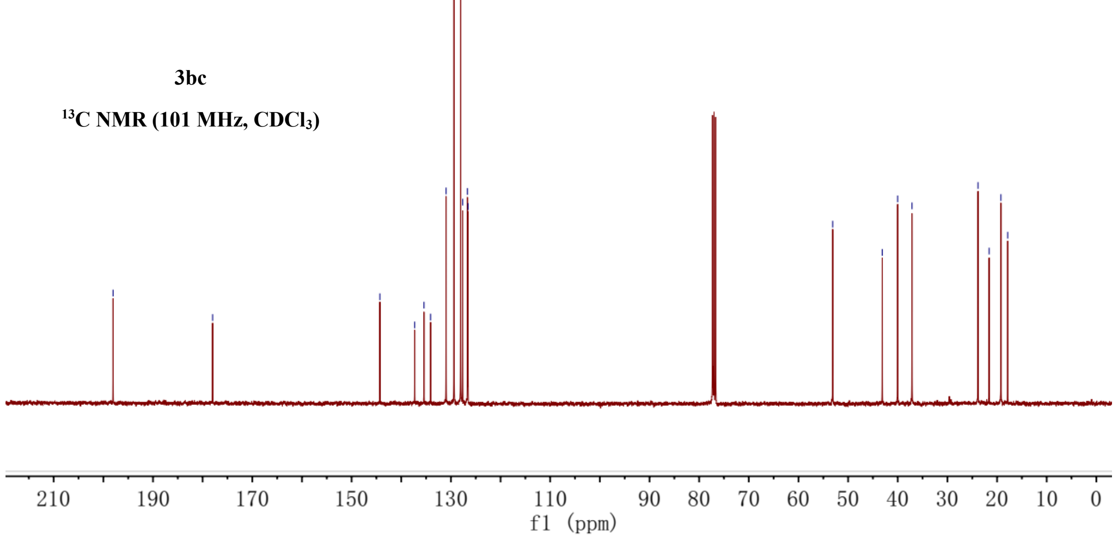

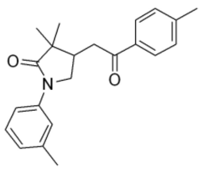

3bd

^{1}H NMR (400 MHz, CDCl$_3$)

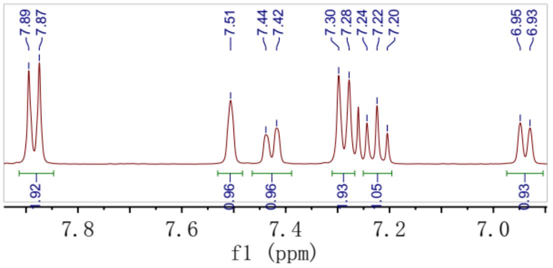

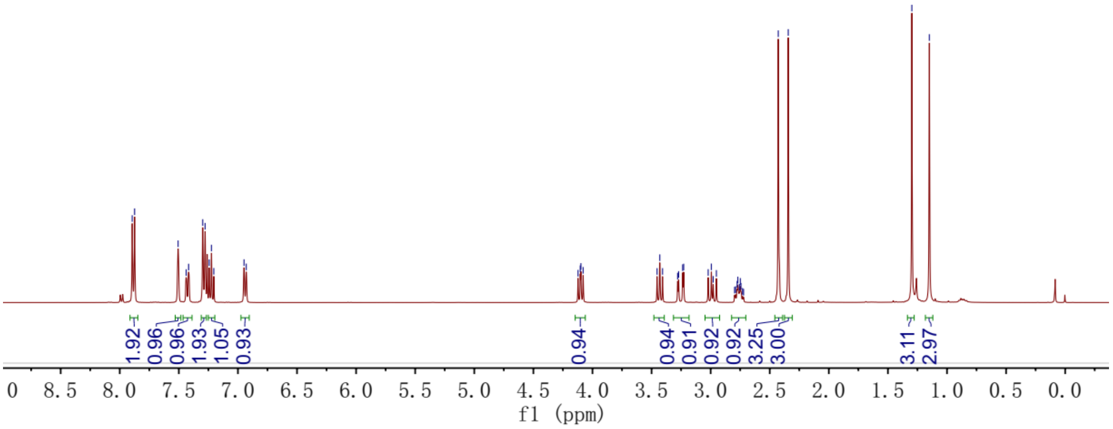

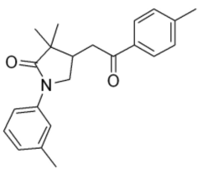

3bd

^{13}C NMR (101 MHz, CDCl$_3$)

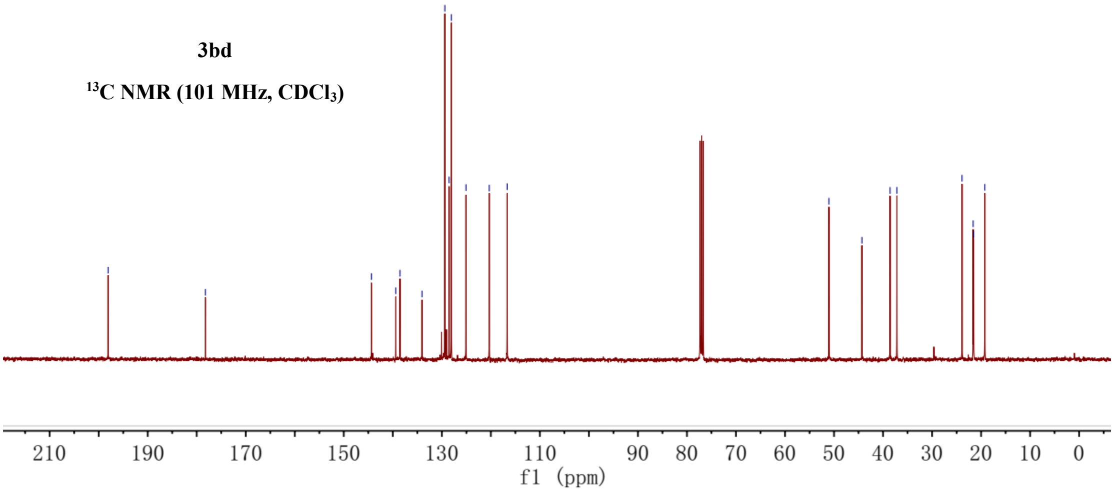

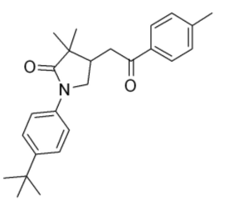

3be

^{1}H NMR (400 MHz, CDCl$_3$)

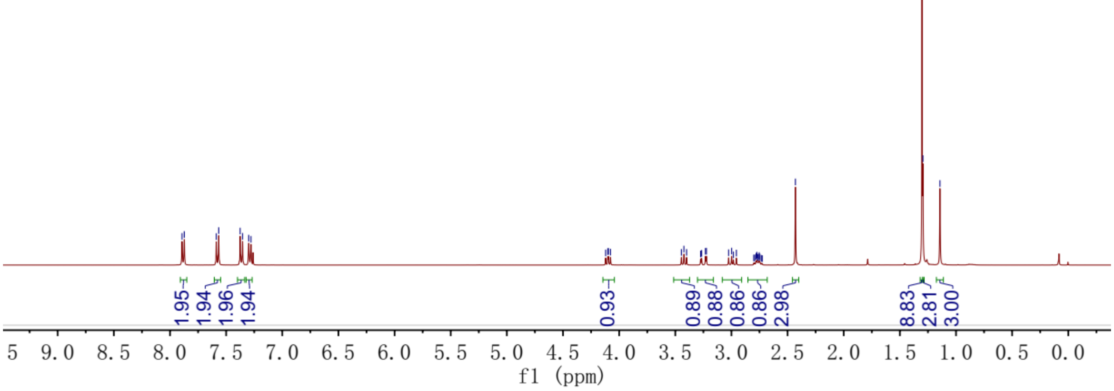

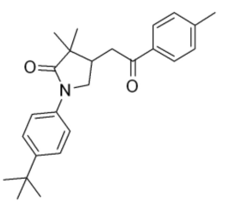

3be

^{13}C NMR (101 MHz, CDCl$_3$)

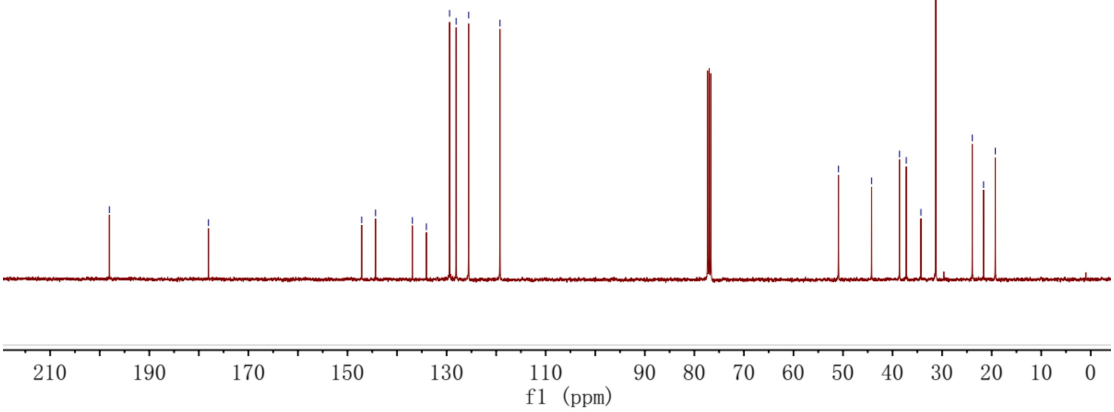

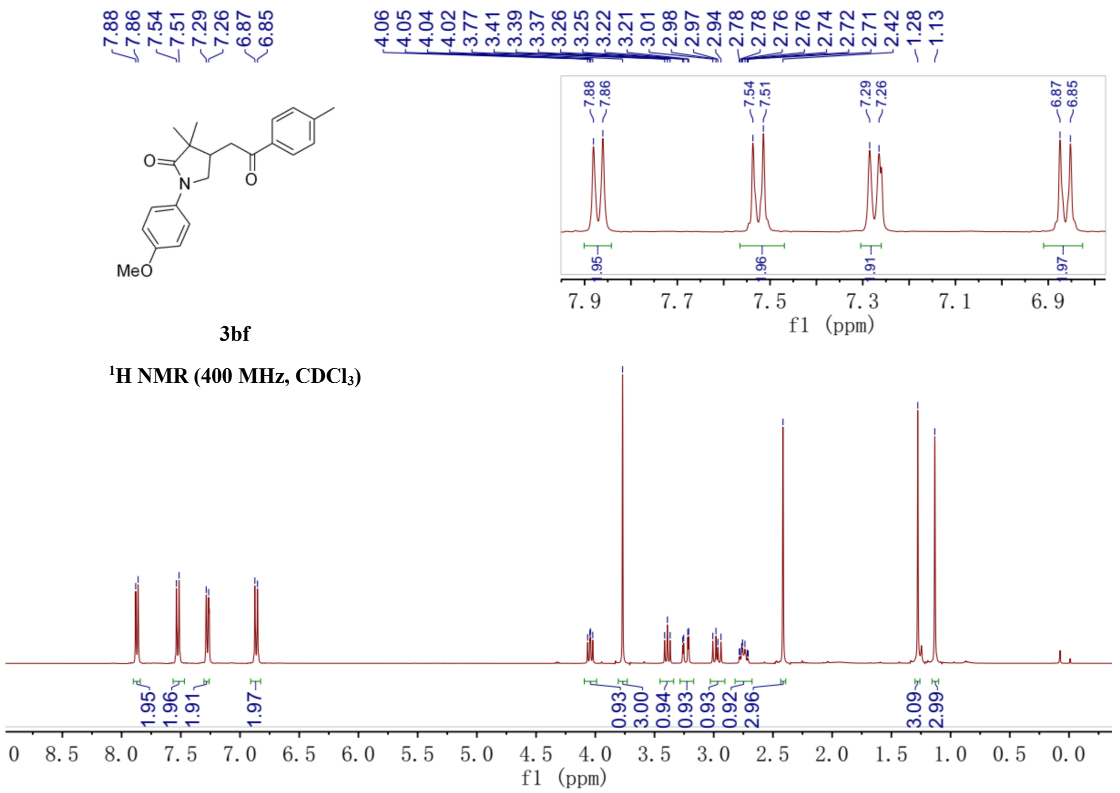

3bf

^{1}H NMR (400 MHz, $CDCl_3$)

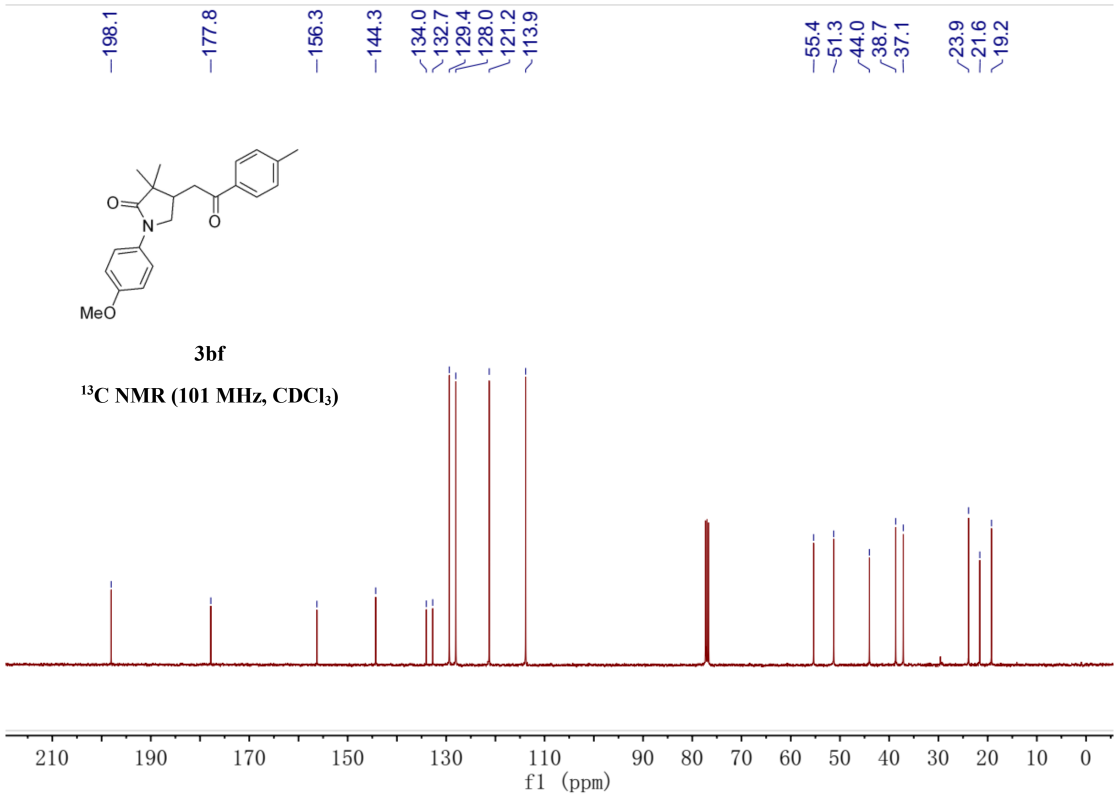

3bf

^{13}C NMR (101 MHz, $CDCl_3$)

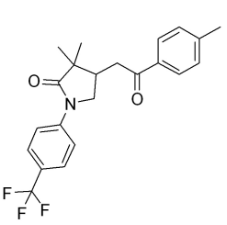

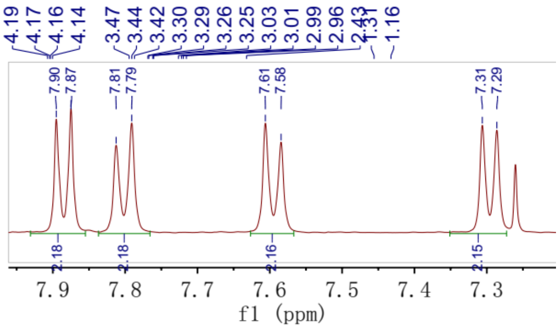

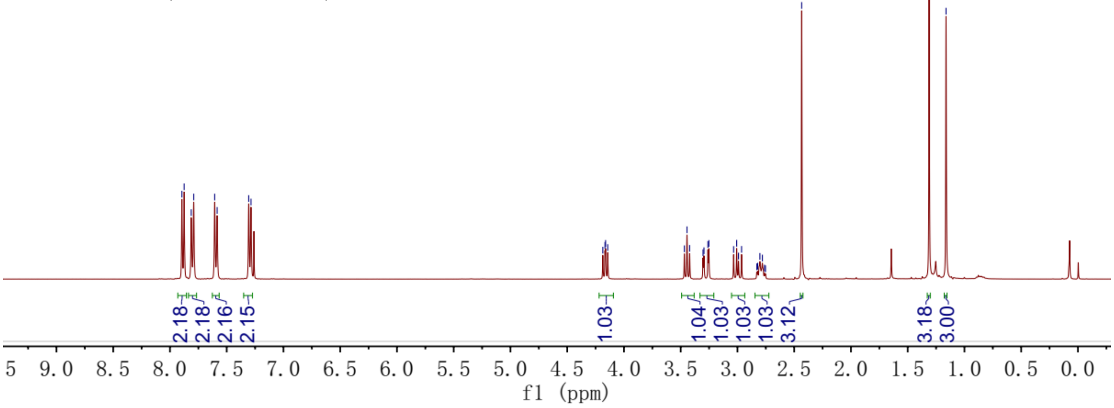

3bg

^{1}H NMR (400 MHz, CDCl$_3$)

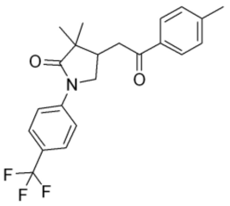

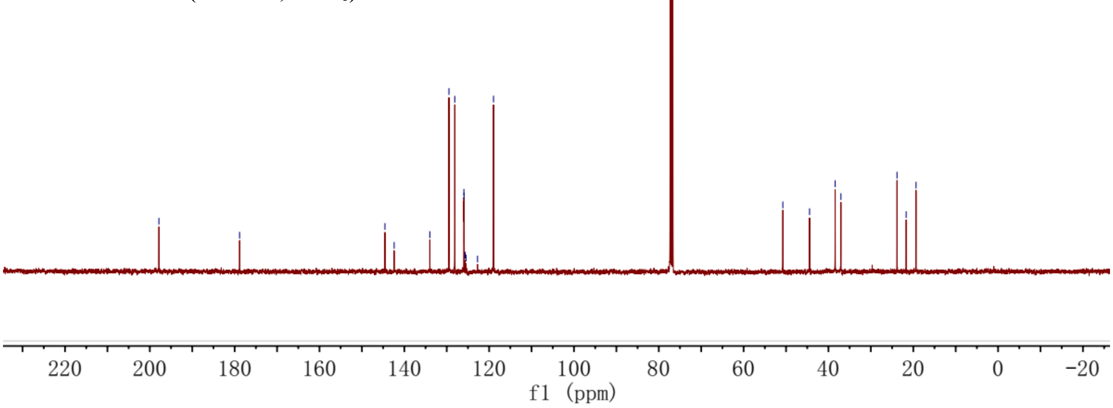

3bg

^{13}C NMR (101 MHz, CDCl$_3$)

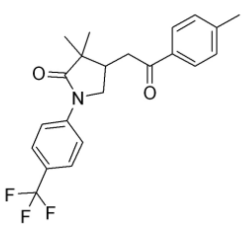

3bg

^{1}F NMR (376 MHz, $CDCl_3$)

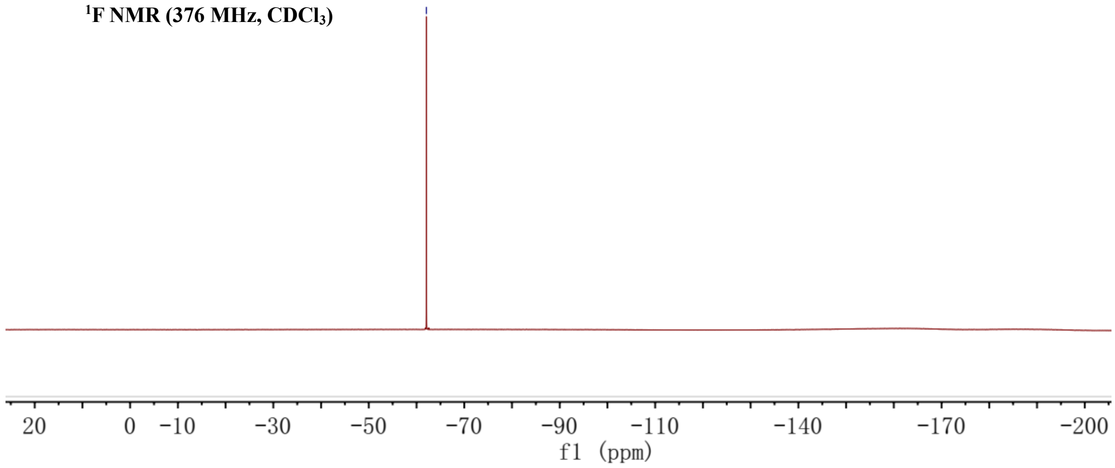

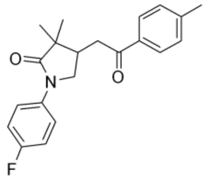

3bh

^{1}H NMR (400 MHz, CDCl$_3$)

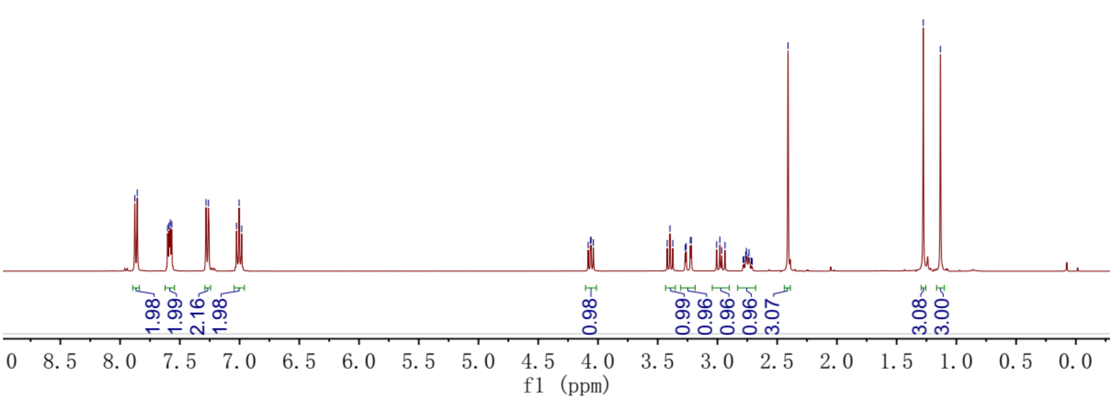

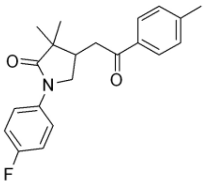

3bh

^{13}C NMR (101 MHz, CDCl$_3$)

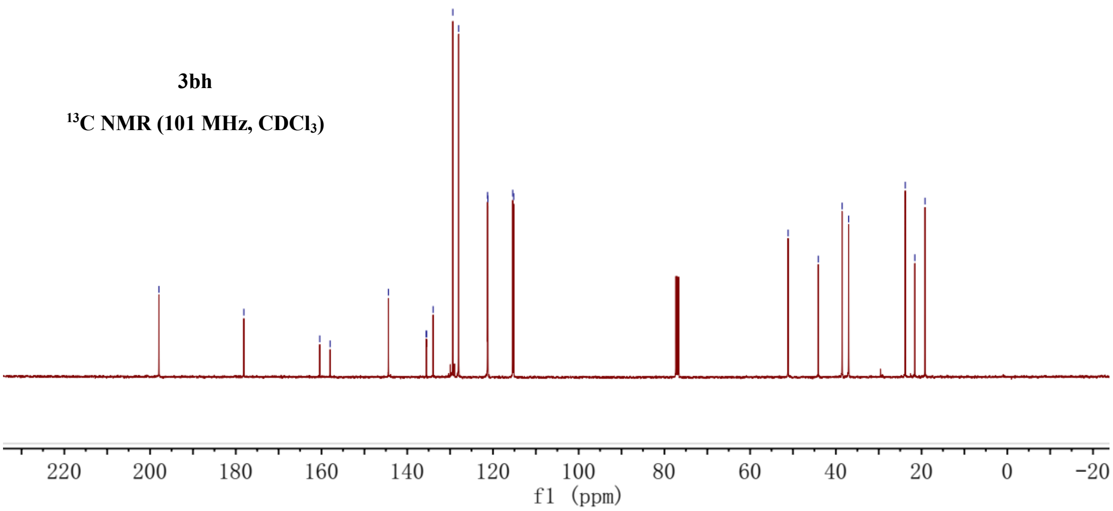

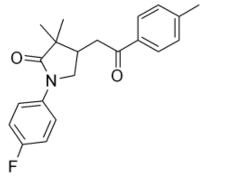

3bh

[1]F NMR (376 MHz, $CDCl_3$)

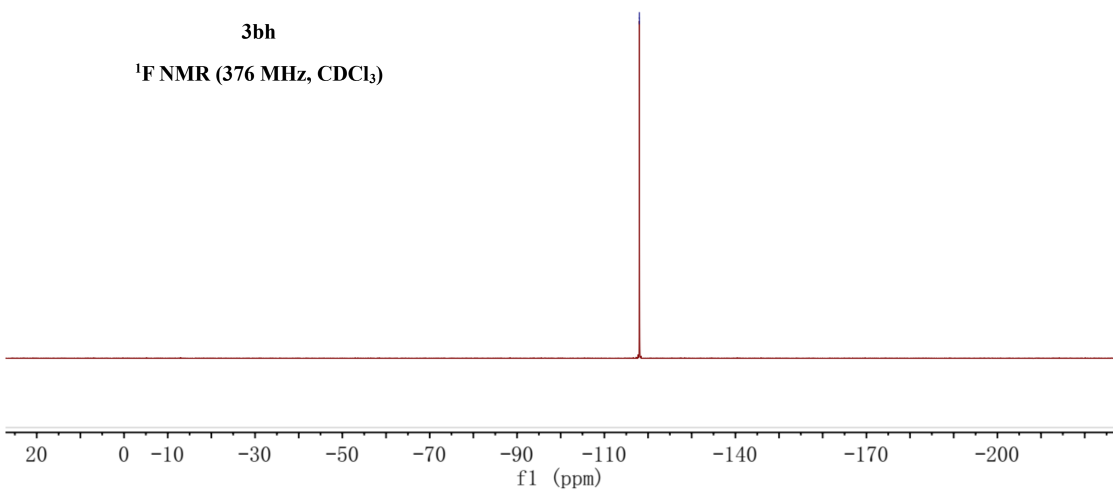

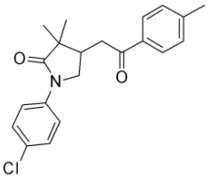

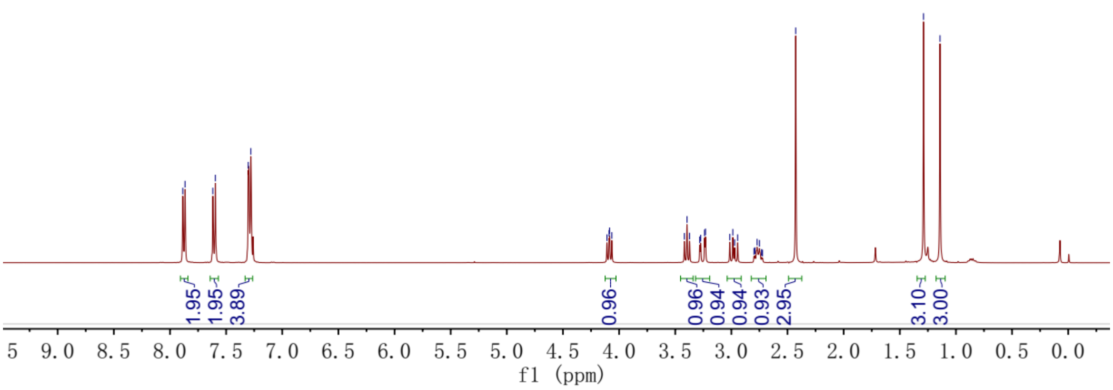

3bi

^{1}H NMR (400 MHz, CDCl$_3$)

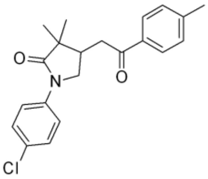

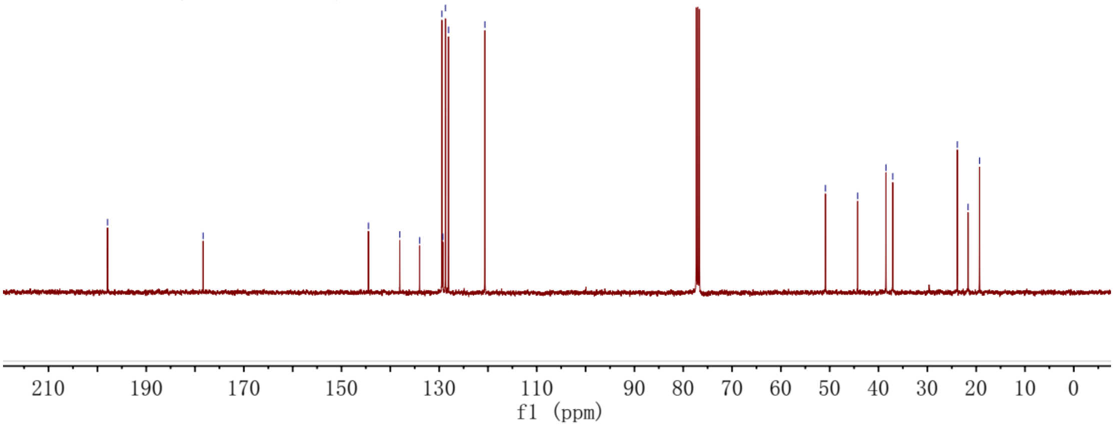

3bi

^{13}C NMR (101 MHz, CDCl$_3$)

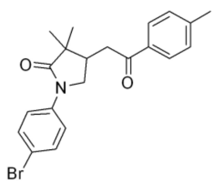

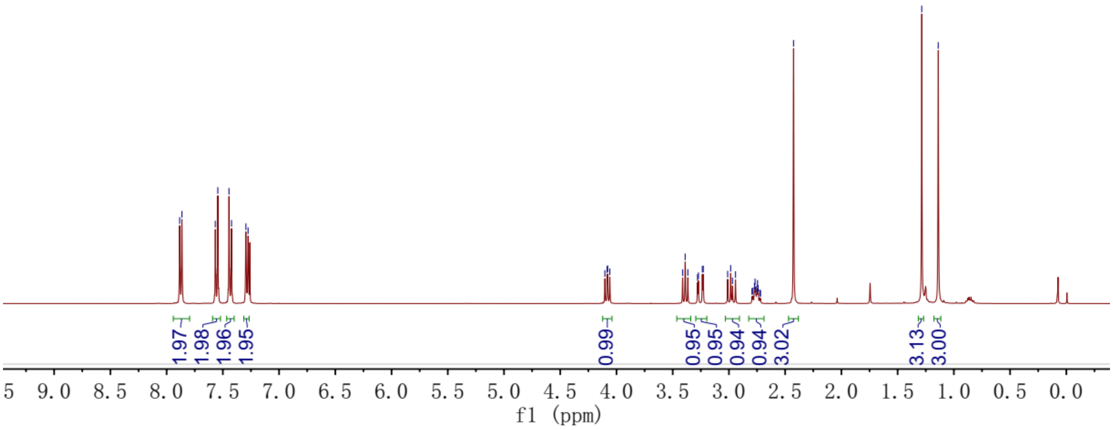

3bj

^{1}H NMR (400 MHz, CDCl$_3$)

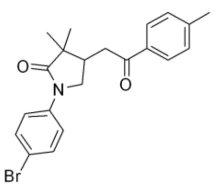

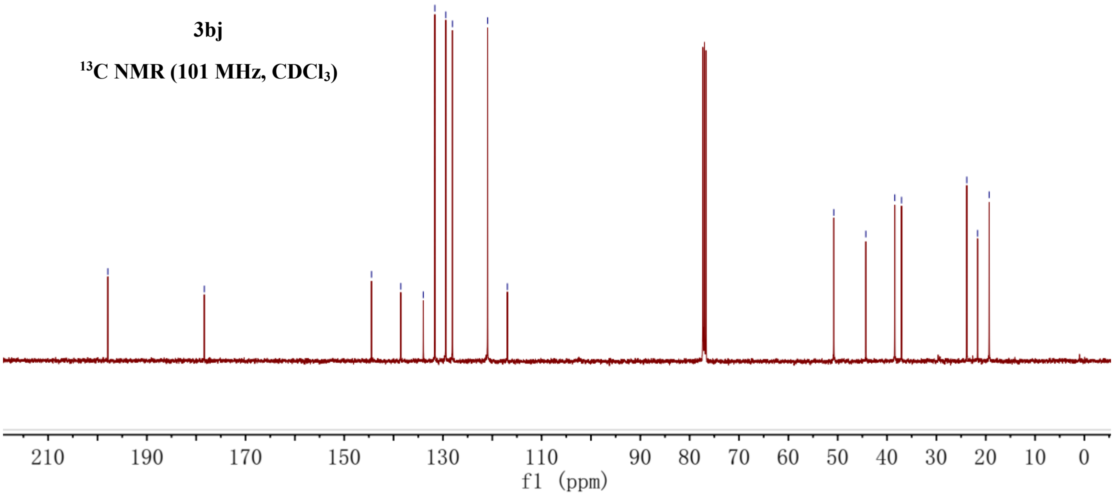

3bj

^{13}C NMR (101 MHz, CDCl$_3$)

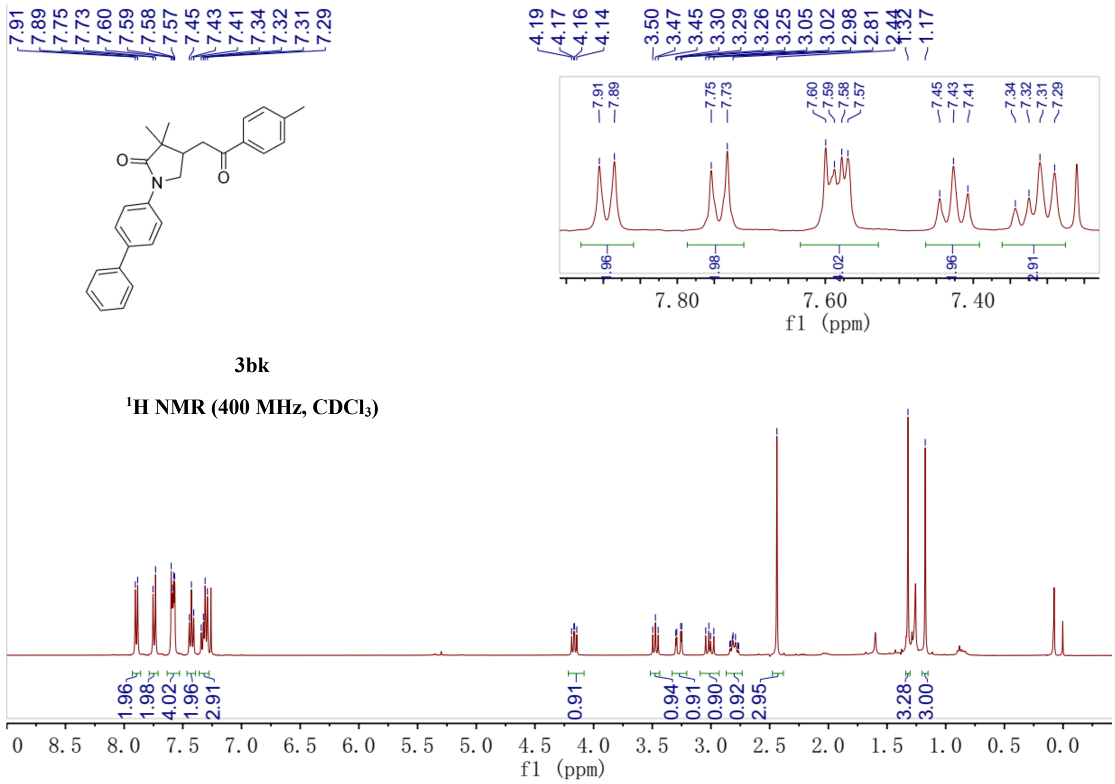

3bk

^{1}H NMR (400 MHz, $CDCl_3$)

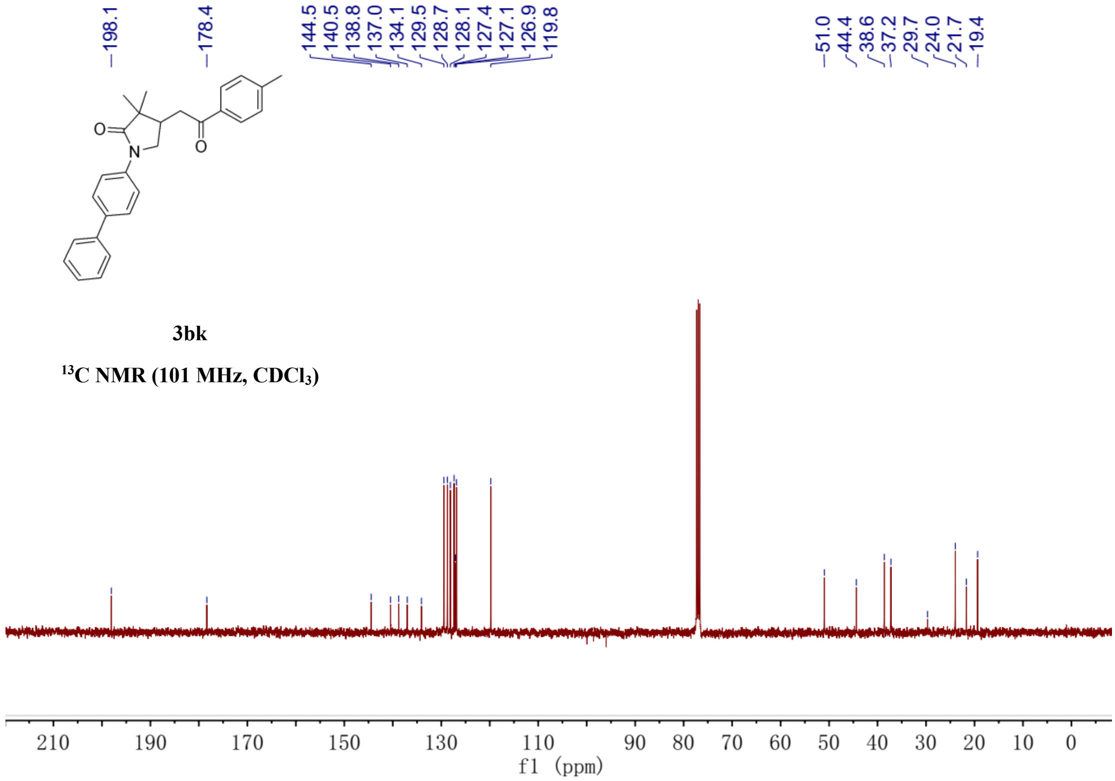

3bk

^{13}C NMR (101 MHz, $CDCl_3$)

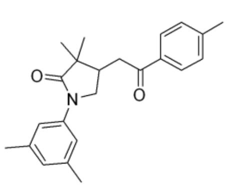

3bl

^{1}H NMR (400 MHz, $CDCl_3$)

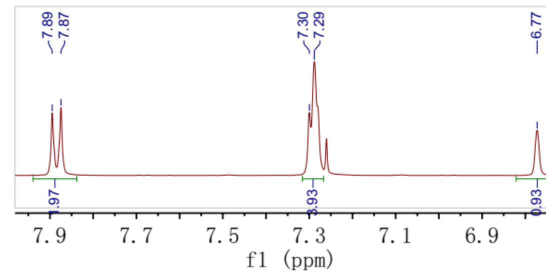

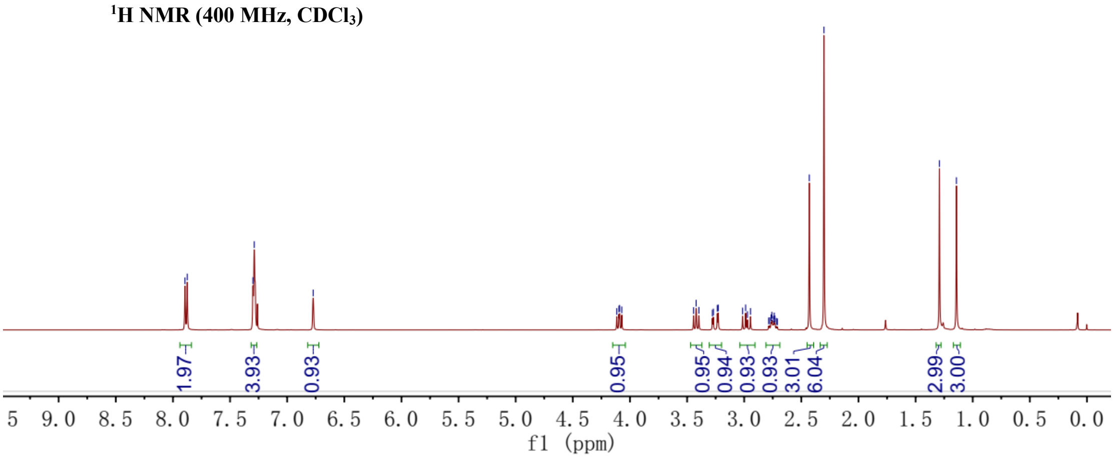

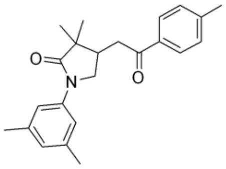

3bl

^{13}C NMR (101 MHz, $CDCl_3$)

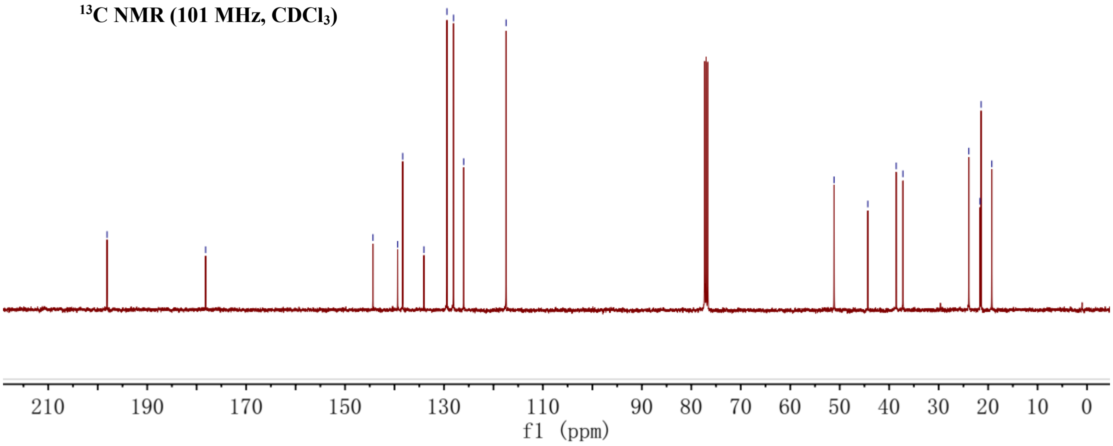

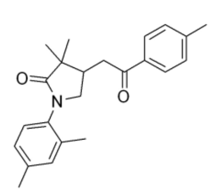

3bm

^{1}H NMR (400 MHz, CDCl$_3$)

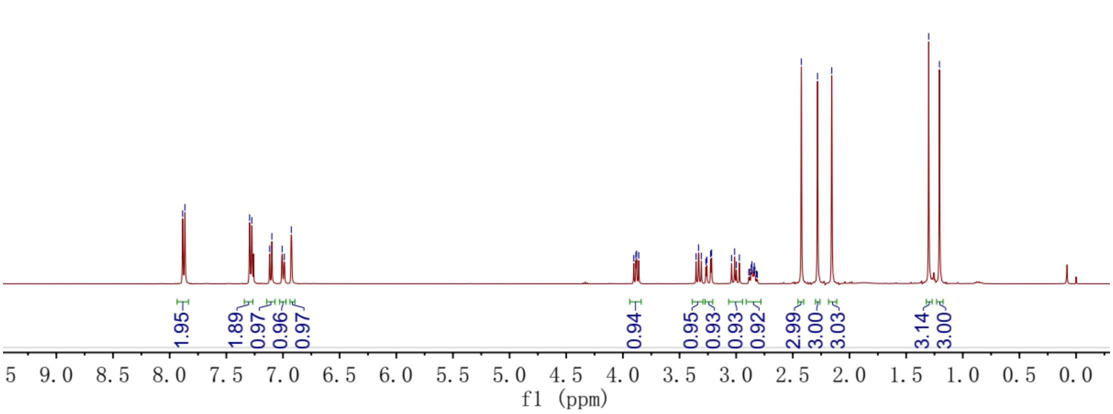

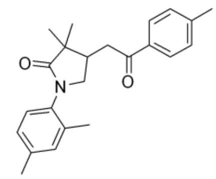

3bm

^{13}C NMR (101 MHz, CDCl$_3$)

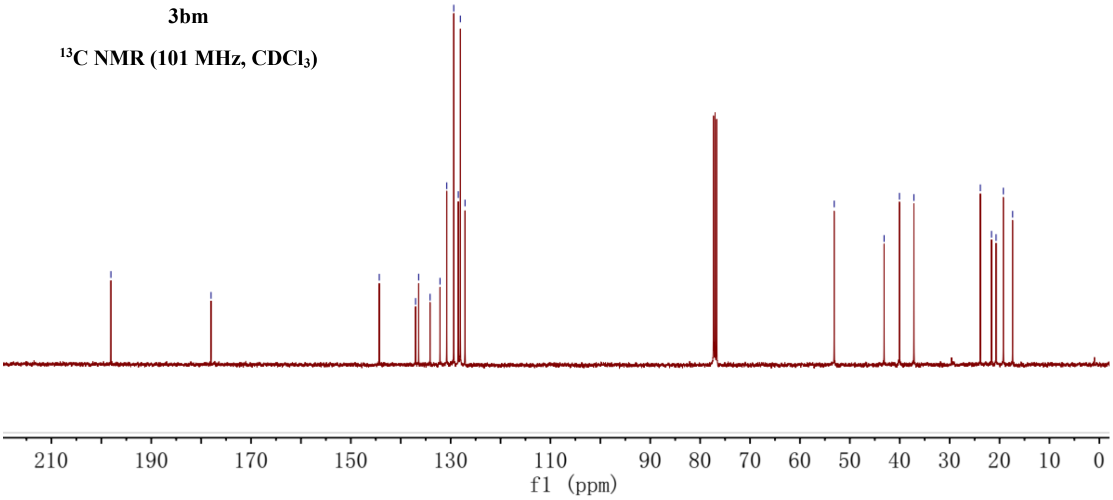

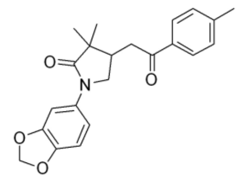

3bn

^{1}H NMR (400 MHz, CDCl$_3$)

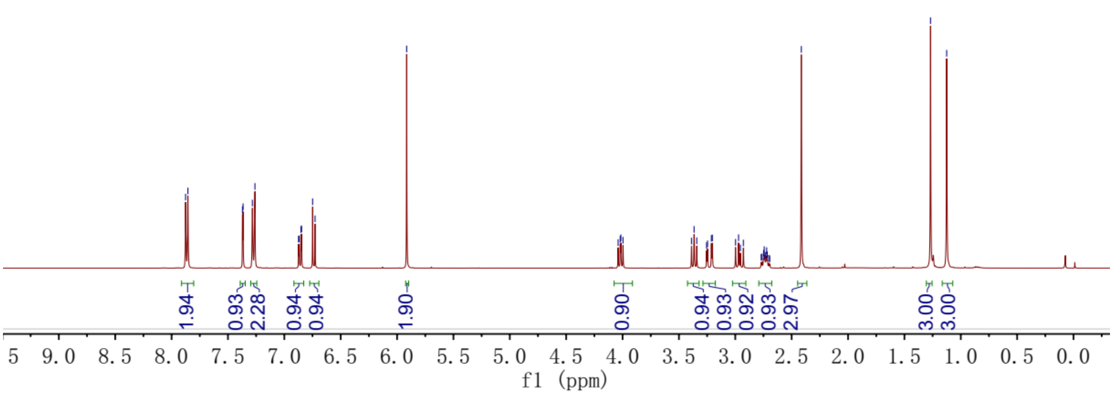

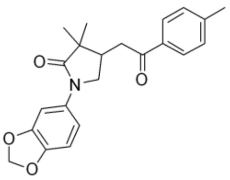

3bn

^{13}C NMR (101 MHz, CDCl$_3$)

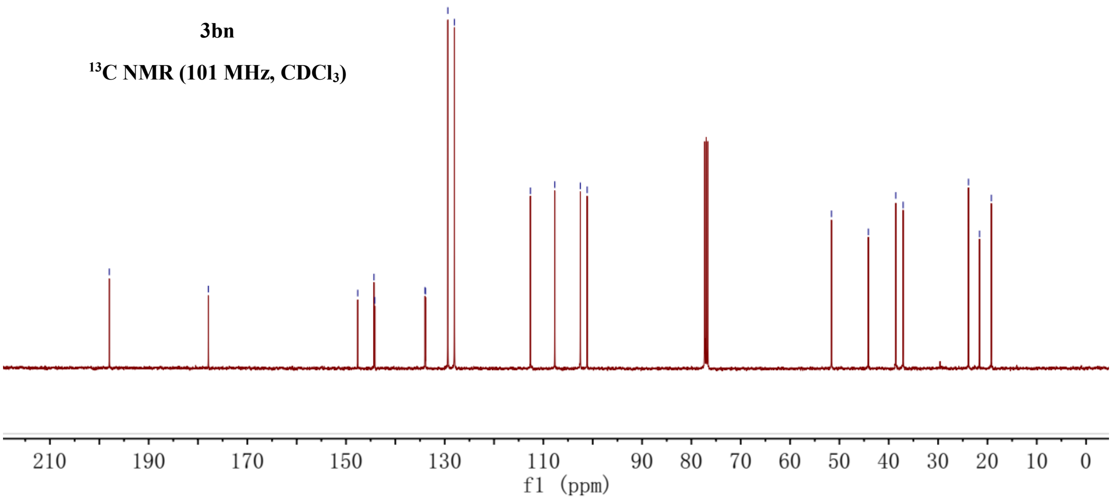

Supplement: Supplementary file 1 — ol5c00003_si_001.pdf [file ol5c00003_si_001.pdf]
